# Human iPSC-derived fallopian tube organoids with *BRCA1* mutation recapitulate early-stage carcinogenesis

**DOI:** 10.1016/j.celrep.2021.110146

**Published:** 2021-12-28

**Authors:** Nur Yucer, Rodney Ahdoot, Michael J. Workman, Alexander H. Laperle, Maria S. Recouvreux, Kathleen Kurowski, Diana J. Naboulsi, Victoria Liang, Ying Qu, Jasmine T. Plummer, Simon A. Gayther, Sandra Orsulic, Beth Y. Karlan, Clive N. Svendsen

**Affiliations:** 1 Board of Governors Regenerative Medicine Institute, Cedars-Sinai Medical Center, Los Angeles, CA 90048, USA; 2 Women’s Cancer Program at the Samuel Oschin Comprehensive Cancer Institute, Cedars-Sinai Medical Center, Los Angeles, CA, USA; 3 Department of Surgery, Samuel Oschin Comprehensive Cancer Institute, Cedars-Sinai Medical Center, Los Angeles, CA 90048, USA; 4 Department of Biomedical Sciences, Cedars-Sinai Medical Center, Los Angeles, CA 90048, USA; 5 Center for Bioinformatics and Functional Genomics, Department of Biomedical Sciences, Cedars-Sinai Medical Center, Los Angeles, CA 90048, USA; 6 Present address: Department of Obstetrics and Gynecology, David Geffen School of Medicine, University of California Los Angeles, Los Angeles, CA 90095, USA; 7 Present address: Jonsson Comprehensive Cancer Center, University of California Los Angeles, Los Angeles, CA 90095, USA; 8 Present address: VA Greater Los Angeles Healthcare System, Los Angeles, CA, USA; 9 Lead contact

## Abstract

Germline pathogenic mutations in *BR*east *CA*ncer (*BRCA1*) genes are thought to drive normal fallopian tube epithelial (FTE) cell transformation to high-grade serous ovarian cancer. No human models capture the sequence of events for disease initiation and progression. Here, we generate induced pluripotent stem cells (iPSCs) from healthy individuals and young ovarian cancer patients with germline pathogenic *BRCA1* mutations (*BRCA1*^*mut*^). Following differentiation into FTE organoids, *BRCA1*^*mut*^ lines exhibit cellular abnormalities consistent with neoplastic transformation compared to controls. *BRCA1*^*mut*^ organoids show an increased production of cancer-specific proteins and survival following transplantation into mice. Organoids from women with the most aggressive ovarian cancer show the greatest pathology, indicating the potential value to predict clinical severity prior to disease onset. These human FTE organoids from *BRCA1*^*mut*^ carriers provide a faithful physiological *in vitro* model of FTE lesion generation and early carcinogenesis. This platform can be used for personalized mechanistic and drug screening studies.

## In brief

Yucer et al. generate a human *BRCA1* mutant iPSC-derived fallopian tube organoid model, which recapitulates *BRCA1* mutant ovarian carcinogenesis *in vitro* and shows tumors *in vivo*. This model provides a biologically relevant platform to validate drugs and a basis for personalized early detection and preventative strategies for women carrying *BRCA1* mutations.

## Graphical abstract

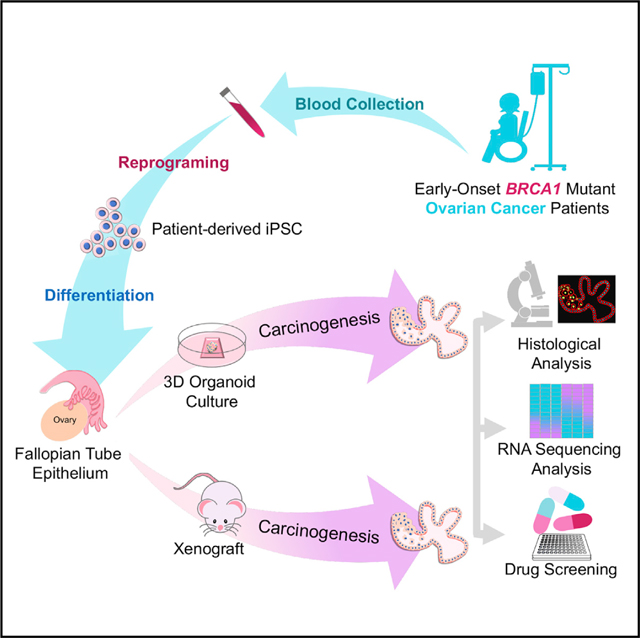

## INTRODUCTION

Ovarian cancer is the leading cause of gynecologic cancer death worldwide, with only slight improvements in overall survival rates over the last 40 years. Epithelial ovarian cancer represents a heterogeneous group of diseases with different cells of origin, histology, risk factors, and biologic behaviors. High-grade serous cancer (HGSC) is the most common and lethal subtype of ovarian cancer, with approximately 70% of newly diagnosed ovarian cancer cases classified as HGSC (Koshiyama et al., 2014, 2017; Kurman, 2013). Patients are typically diagnosed with late-stage (III/IV) clinical disease and present with wide-spread peritoneal metastases. The strongest genetic risk factor for developing HGSC is carrying a germline mutation in the *BR*east *Ca*ncer (*BRCA1*) gene (*BRCA1*^*mut*^). The risk of ovarian cancer in the general population is approximately 1.37% (Pearce et al., 2015), which jumps over 30-fold to 40%–59% among individuals that carry *BRCA1*^*mut*^ (Kuchenbaecker et al., 2017). *BRCA1* functions in the DNA break repair pathway through deficiencies in homologous recombination (HR). Nearly one-half of all HGSCs are HR deficient, most commonly from the loss of *BRCA* function due to germline or somatic mutations in the *BRCA* genes or loss of *BRCA1* expression due to promotor methylation (Press et al., 2008).

Historically, ovarian cancer was believed to originate from the ovarian surface epithelium. Yet, recent studies have indicated that fallopian tube epithelial (FTE) cells are a common cell of origin for HGSC particularly in germline *BRCA1*^*mut*^ carriers (Kurman, 2013; Labidi-Galy et al., 2017). The discovery that *BRCA1*^*mut*^ patients undergoing risk-reducing surgery harbor serous tubal intraepithelial carcinoma (STIC) lesions in the FTE further supports the hypothesis that FTE is the predominant cell of origin of HGSC (Jones and Drapkin, 2013). As part of the STIC sequence, most pre-neoplastic lesions initially demonstrate nuclear accumulation of Tumor Protein p53 (TP53) and develop a “p53 signature,” prior to STIC and invasive serous carcinoma (Kuhn et al., 2012). Additional pathologic studies have supported STIC lesions as the precursor for HGSC in high-risk women carrying germline *BRCA1* or *BRCA2* mutations and in up to 60% of sporadic HGSC patients (Bolton et al., 2012; Labidi-Galy et al., 2017).

Immortalized cell lines and murine models have been used extensively to model the early-stage development of HGSCs from FTE cells, which includes the Brca;Tp53;Pten or Tp53(R172H) mutant with a Dicer/Pten double knockout mouse model that shows FTE transformation and HGSC initiation (Kim et al., 2015; Perets et al., 2013). But, few human-based *in vitro* models of FTE cells accurately recapitulate early-stage development of HGSC. The recent use of three-dimensional (3D) “organoid” models derived from human induced pluripotent stem cells (iPSCs) provides a powerful tool that can replicate physiologically relevant processes of disease *in vitro* (Liu et al., 2018; Sharma et al., 2020). This model system can self-renew and differentiate into multiple lineages and intrinsically self-organize to form 3D tissue architecture (Yamanaka, 2012). Importantly, iPSCs derived from patients with known genetic mutations will harbor not only the disease mutation but also the patient’s whole genetic background, which can help to elucidate how known and unknown mutations can together contribute to HGSC susceptibility, initiation, and progression (Curry et al., 2015). Several iPSC-derived disease models have been used to reproduce inherited high-risk cancers, such as an osteosarcoma model for Li-Fraumeni syndrome that recapitulated cancer pathology. (Crespo et al., 2017; Curry et al., 2015; Lee et al., 2015). BRCA1 patient-derived iPSCs have also been recently generated and provide an ideal model to study breast cancer events (Portier et al., 2021; Silva et al., 2021a, 2021b; Soyombo et al., 2013).

This study investigated early genomic alterations and carcinogenesis in patients with a *BRCA1*^*mut*^ by using an iPSC-based disease model of FTE. Three different *BRCA1*^*mut*^-iPSC lines from patients with early-onset cancer were derived and 3D human *BRCA1*^*mut*^-FTE organoids were generated. Immunocytochemical staining of secretory and ciliated cellular components demonstrated that these organoid structures model human FTE. The *BRCA1*^*mut*^-FTE organoids showed structural abnormalities, including cellular crowding, loss of polarity, and severe atypia of the nuclei, which are signatures of the STIC. This finding indicates that *BRCA1*^*mut*^-FTE organoids recapitulate cancer-initiating events and create an *in vitro* system that can be used to model *BRCA1*^*mut*^-based ovarian cancer at early stages. Molecular signatures of this initial event in *BRCA1*^*mut*^-FTE organoids and in long-term cultures were characterized. Finally, the neoplastic transformation capacity and cancerous features of *BRCA1*^*mut*^-FTE organoids were explored using a mouse model. This human-derived FTE organoid model can be used to identify the critical sequence of genetic alterations involved in the carcinogenesis of HGSC and specific biomarkers of early FTE cell transformation.

## RESULTS

### *BRCA1^mut^*-iPSCs generated from early-onset ovarian cancer patients can be differentiated into FTE organoids

**Figure 1.**
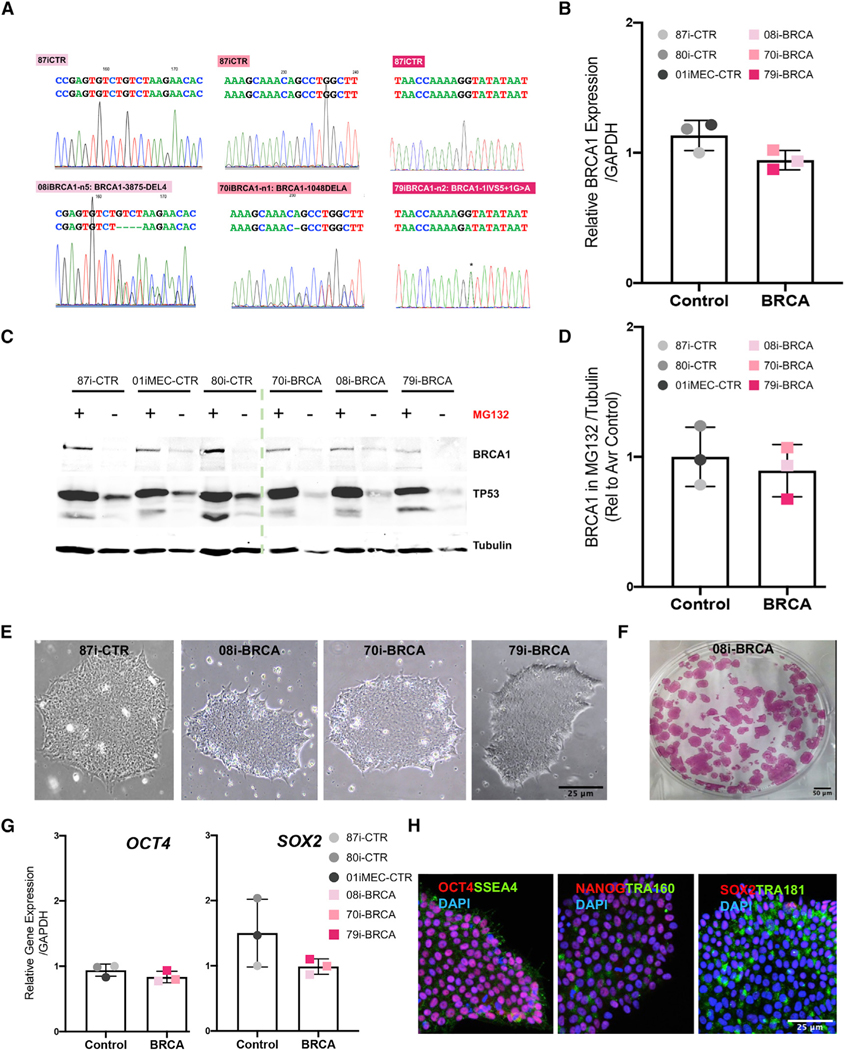
Characterization of *BRCA1*^*mut*^ and control iPSC lines Lines 87i-CTR-n3, 80i-CTR-Tn3, 01iMEC-CTR-n4, 08i-BRCA-n5 and -n8, 70i-BRCA-n1 and -n2, and 79i-BRCA-n2 were characterized. (A) Sequence analysis of heterozygous *BRCA1*^*mut*^ in iPSC lines 79i-BRCA (IVGS5+1G > A located at the junction between exon 5 and intron 6), 70i-BRCA (1048delA), and 08i-BRCA (3875del4 located in exon 11). Heterozygous positions are indicated in comparison to 87i-CTR. (B) *BRCA1* gene expression in all controls and *BRCA1*^*mut*^-iPSC lines. (C) Western blot for BRCA1, TP53, and Tubulin protein in iPSC lines after a 24-h treatment with the MG132 proteasomal inhibitor. (D) BRCA1 protein normalized to Tubulin and quantified from multiple iPSC lines, with each point representing the band intensity from a separate line. (E) Brightfield image of iPSC colonies for 87i-CTR, 08iBRCA, 70iBRCA, and 79iBRCA. (F) Representative image of alkaline phosphatase (AP) staining of the 08i-BRCA line. (G) Relative gene expression of *OCT4* and *SOX2* genes in all iPSC lines. Relative gene expression to 87i-CTR calculated using DDCt method and normalized to the endogenous *GAPDH* level. (H) Immunocytochemistry of the 79i-BRCA iPSC line for pluripotent stem cell markers OCT4, NANOG, SOX2, TRA160, TRA181, and SSEA4. Scale bars, 25 μm. Error bars are standard error of the mean (SEM) (n = 3 independent biological experiments and each dot represents the average of 3 independent biological experiments per line).

We previously developed a rapid and efficient method to create iPSC-derived 3D human FTE organoids that contained relevant cell types of the human FTE and a luminal architecture that closely reflects the organization of FTE tissues *in vivo* (Yucer et al., 2017). Briefly, modulation of BMP and WNT signaling (details in the STAR Methods section) was used to direct iPSC differentiation into Müllerian cells, and subsequent use of pro-Müllerian growth factors promoted differentiation into fallopian tube-like precursor cells. A 3D growth platform enabled the FTE organoid to self-organize into a convoluted luminal structure, permitting matured differentiation to a FTE lineage. Here, iPSCs were generated from three patients diagnosed with epithelial ovarian cancer, and all carried germline pathogenic mutations in the *BRCA1* gene and three unaffected, non-BRCA1 mutation controls (Barrett et al., 2014). Genetic and clinical characteristics are provided in Table 1. Sequencing confirmed the presence of *BRCA1* heterozygous mutations in iPSC lines (Figure 1A), with each patient having a different heterozygous mutation—79i-BRCA (IVGS5+1G > A, located at the junction between exon 5 and intron 6), 70i-BRCA (1048delA), and 08i-BRCA (3875del4, located in exon 11)—with all mutations predicted to be protein truncating and highly penetrant. qRT-PCR demonstrated that the *BRCA1*^*mut*^ does not discernably alter *BRCA1* transcript expression in iPSC lines from carriers versus non-carriers (Figure 1B). For western blot analysis, cultures were treated with the proteasomal inhibitor MG132 in order to facilitate the detection of BRCA1 that has low production under non-stress conditions. Although the 79i-BRCA line showed lower production, there was no significant difference overall between the *BRCA1*^*mut*^ and control iPSC lines (Figures 1C and 1D). Both *BRCA1*^*mut*^ and control iPSC lines displayed an embryonic stem cell-like morphology and exhibited pluripotent properties, including alkaline phosphatase activity, RNA expression of pluripotency factors (*OCT4* and *SOX2*), and protein production of pluripotency markers (Figures 1E–1H). Clones of *BRCA1*^*mut*^ and control iPSC lines exhibited normal karyotypes even after extensive culturing at passage 32 (Figure S1). Differentiation into three germ layers by using established protocols confirmed the differentiation capacity of *BRCA1*^*mut*^-iPSCs (Figure S2A; Chambers et al., 2009; Mae et al., 2013; Teo et al., 2014; Yucer et al., 2017). RNA expression analysis confirmed that control and *BRCA1*^*mut*^-iPSC lines had the capacity to differentiate into ectoderm-expressing *PAX6*, mesoderm-expressing *BRACHYURY*, and endoderm-expressing *SOX17*, with only slight variations in differentiation levels between lines (Figure S2A).

**Figure 2.**
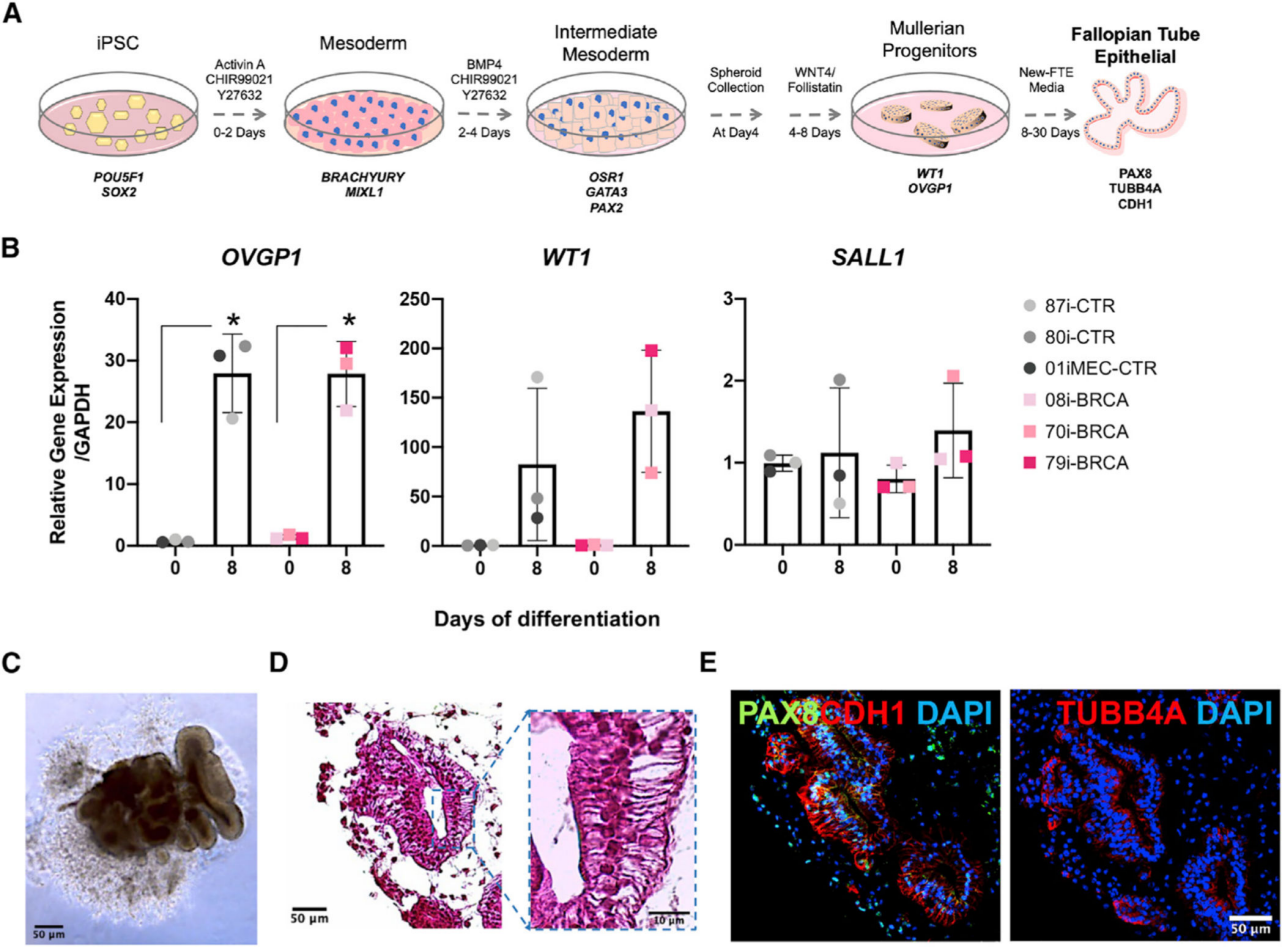
*BRCA1*^*mut*^-iPSC-derived FTE organoids share characteristics of primary human FTE tissues (A) Timeline and factors for iPSC differentiation into FTE organoids. Control lines were reprogrammed from healthy female donor cells with peripheral blood mononuclear cells (PBMCs) used for CS80i-CTR-Tn3, lymphoblastoid cells (LCLs) used for CS87i-CTR-n3, and mammary epithelial cells used for CS01iMEC-CTR-n4. Lines CS08i-BRCA-n5, CS70i-BRCA-n1, and CS79i-BRCA-n2 were reprogrammed from LCLs from ovarian cancer patients with *BRCA1*^*mut*^. (B) qRT-PCR quantification of gene expression for Müllerian duct markers, *WT1* and *OVGP1*, and kidney markers *SALL1* at day 8 of Müllerian duct differentiation. (C and D) Brightfield image and H&E stain of *BRCA1*^*mut*^*-*FTE organoid (70i-BRCA line) at day 30 and high-magnification image. Scale bars, 50 mm and 10 mm. (E) Immunocytochemistry for FTE markers PAX8 and TUBB4A and epithelial marker CDH1 in *BRCA1*^*mut*^*-*FTE organoids (70i-BRCA line) at day 30 in culture. Scale bar, 50 μm. Relative gene expression at iPSC stage (day 0) calculated using the ΔΔCt method and normalized to the endogenous *GAPDH* level for the 87i-CTR iPSC line. Error bars are SEM (n = 3 independent biological experiments). Student’s t test was used, *p ≤ 0.05.

In order to elucidate the role of *BRCA1*^*mut*^ in ovarian cancer initiation in the FTE, we used our previously established protocol to generate iPSC-derived *BRCA1*^*mut*^-FTE organoids (Figure 2A; Yucer et al., 2017). The efficiency of FTE differentiation varied between lines, but all had the capacity to generate FTE cells. Quantifying the expression of key markers demonstrated differentiation into the mesoderm with *BRACHYURY* and *MIXL1* and intermediate mesoderm with *PAX2*, *GATA3*, and *OSR1* (Figures S2B and S2C). Consistent with our previous findings with control iPSC lines (Yucer et al., 2017), an increased expression of *OVGP1* and *WT1* over 8 days in culture confirmed that *BRCA1*^*mut*^-iPSC lines can produce a pre-Mullerian duct, indicating they model the precursors of FTE (Figure 2B). Lack of expression of the kidney marker *SALL1* verified the specificity of Müllerian duct differentiation (Figure 2B). After 30 days in culture, *BRCA1*^*mut*^-FTE iPSCs formed 3D organoid structures with defined lumens lined by the cuboidal epithelial cells (Figures 2C and 2D). Importantly, these organoids expressed the epithelial marker CDH1 as well as FTE secretory and ciliated cell markers PAX8 and TUBB4A (Figure 2E; Figure S2D). TUBB4A production was restricted to the apical surface of columnar epithelial cells, consistent with the establishment of polarized epithelia.

### *BRCA1^mut^*-FTE organoids exhibit precancerous pathological changes observed in pre-neoplastic ovarian cancer lesions

**Figure 3.**
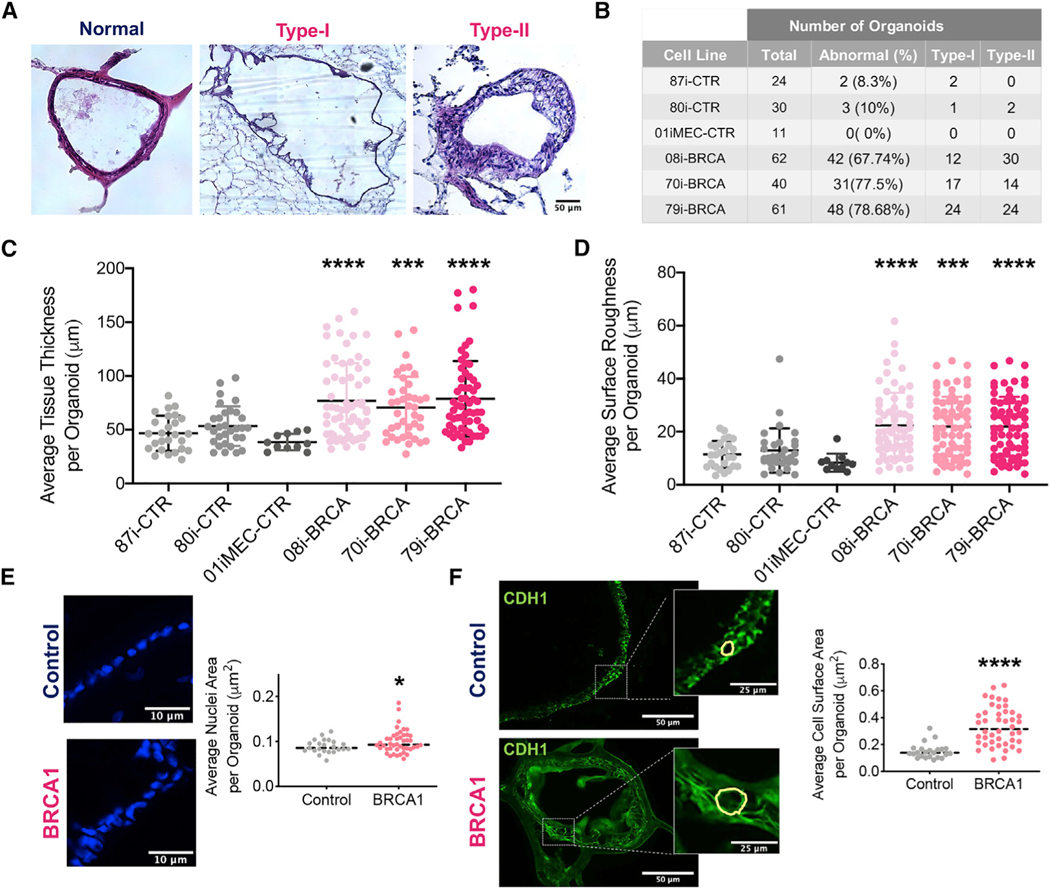
*BRCA1*^*mut*^-FTE organoids exhibit histopathological features of STIC (A) Representative H&E stain of different histopathology for organoids at 4 months in culture, compared to 80i-CTR line. Type I obtained from the 08i-BRCA line and Type II obtained from the 79i-BRCA line, with images stitched using Stereo Investigator. Scale bar, 50 μm. (B) Table for total organoids analyzed in each cell line at 4 months (n = 5 different biological replicates used per cell line). (C) FTE organoid thickness distribution by individual lines. (D) Cellular outgrowth measured as arithmetic average roughness by individual lines. (E and F) Nuclei and cell surface distribution based on DAPI and CDH1 staining and corresponding quantification. Scale bars, 10 μM (E) 50 and 25 μm (F). (n = 3 different biological replicates used per organoid). Significance calculated using the Student’s t test and Welch ANOVA;*p < 0.05, ***p < 0.001, and ****p < 0.0001.

**Figure 4.**
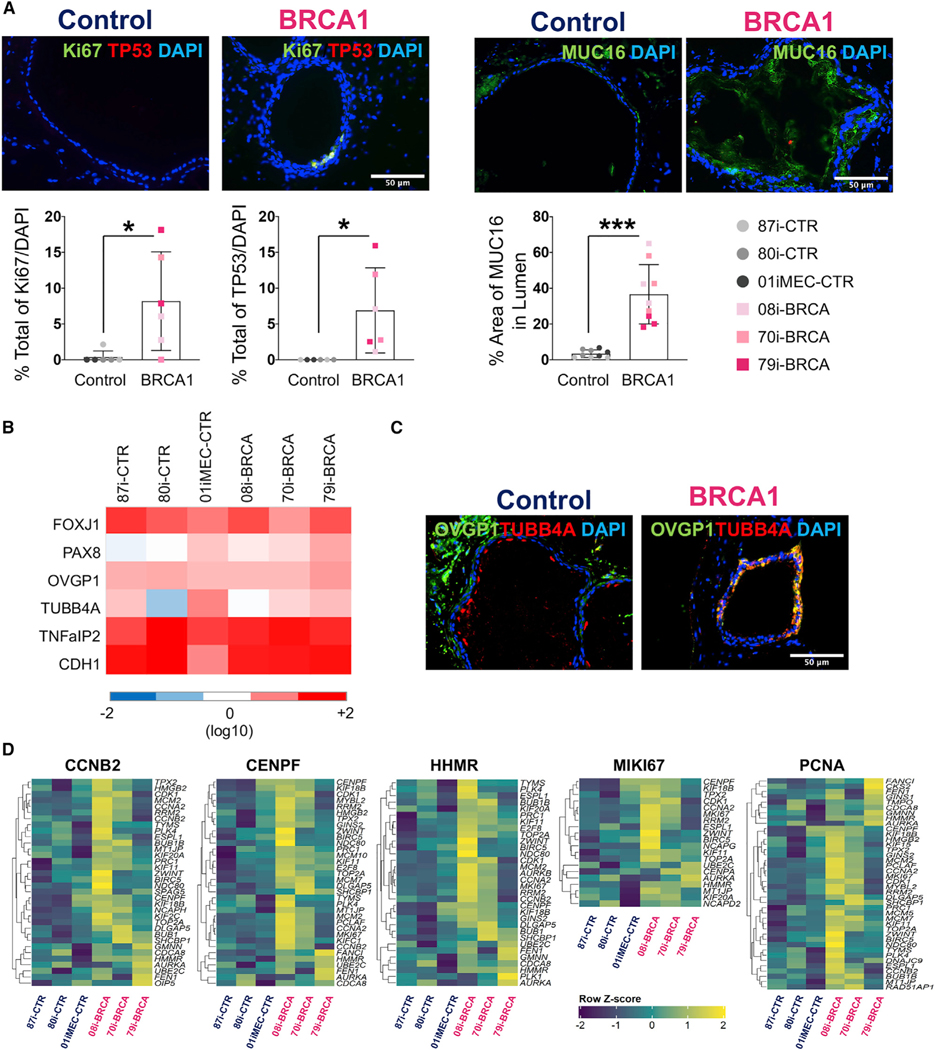
*BRCA1*^*mut*^-iPSC-derived FTE organoids recapitulate the molecular signature of STIC *in vitro* (A) Immunocytochemistry for TP53 and Ki67 and neoplastic marker MUC16 and quantification. Percentage of Ki67 and TP53/DAPI and area of MUC16 in lumen were calculated for 3 different control organoids and 3 different *BRCA1* organoids (n = 2 different biological replicates for Ki67 and TP53, n = 3 different biological replicates for MUC16). (B) Gene expression of FTE markers *FOXJ1*, *PAX8*, *OVGP1*, TUBB4A, *TNFaIP2*, and *CDH1* at 4 months of culture. Color matrix of the heatmap shows TPM (transcripts per kilobase million). (C) Immunocytochemistry for FTE secretory cell marker OVGP1 and ciliated cell marker TUBB4A in control and *BRCA1*^*mut*^-FTE organoids at 4 months. (D) Differential gene expression and GSEA for n = 3 different control and n = 3 different *BRCA1*^*mut*^*-*FTE organoids at 4 months in culture. False discovery rate (FDR) value is <0.05. *Z* scores of the leading-edge genes were calculated and used to construct a heatmap. Scale bars, 50 mm. Significance was calculated using the Student’s t test; *p < 0.05 and ***p < 0.001.

The pathological, phenotypic, and molecular properties of *BRCA1*^*mut*^-FTE organoids were compared to those of the control FTE organoids and human STICs, which are considered a precursor lesion of HGSC. A STIC lesion is defined based on distinct morphological and immunohistochemical features including severe nuclear atypia, architectural alterations, cellular crowding, loss of polarity, increased mitosis, and loss of ciliated cells (Vang et al., 2013; Voltaggio et al., 2016). Hematoxylin and eosin (H&E) staining was performed in order to determine whether *BRCA1*^*mut*^-FTE organoids cultured for 4 months develop STIC pathology. Compared to control organoids that exhibited a normal cuboidal cell morphology, *BRCA1*^*mut*^-FTE organoids displayed severe structural abnormalities with two distinct morphologies classified as Type I with a micropapillary-like structure or Type II with increased cell layers and nuclear stratification (Figures 3A and 3B). Organoids from all *BRCA1*^*mut*^-FTE lines had significantly varied tissue thickness distribution compared to controls, suggesting alteration in normal tissue architecture (Figure 3C; Figures S3A and S3B). To further analyze the Type I tissue architecture, an unbiased mathematical approach estimated cellular outgrowth incident or micropapillary-like structure formation based on the average surface roughness parameter (Figure S3C). Similar to the results from STIC histology, cellular outgrowths were significantly greater in organoids from all *BRCA1*^*mut*^-FTE lines than those in the controls, which is consistent with precancerous characteristics (Figure 3D; Figure S3D). We next investigated changes in nuclear atypia and structural abnormalities at the cellular level in 4-month cultures of *BRCA1*^*mut*^-FTE organoids. Calculating average nuclear area and surface of individual cells per organoid with 4’,6-diamidino-2-phenylindole (DAPI) and CDH1 staining showed that *BRCA1*^*mut*^-FTE organoids displayed cytological changes, including nuclear enlargement, cell surface distortion, and loss of columnar architecture (Figure 3E and 3F). Immunocytochemistry was used to characterize the pathology in 4-month-old *BRCA1*^*mut*^-FTE organoids. The production of TP53 and Ki67, which are hallmark proteins of STIC lesions that are expressed in dysplastic tissues and are used to confirm the preinvasive nature of cancer, indicates that *BRCA1*^*mut*^-FTE organoids exhibit precancerous characteristics similar to STIC *in vitro* (Figure 4A; Figure S4A). Glycoprotein CA125A (cancer antigen 125, also known as mucin 16 [MUC16]), which is overexpressed in most serous ovarian cancers and is used as a diagnostic serum biomarker, was also assessed (Felder et al., 2014). MUC16 protein promotes invasive behavior in preclinical models of cancer, both *in vitro* and *in vivo* (Rao et al., 2015). Remarkably, MUC16 production was considerably higher in *BRCA1*^*mut*^-FTE organoids and released into the lumen, suggesting that the *BRCA1*^*mut*^-FTE organoids have already acquired neoplastic features (Figure 4A). *BRCA1*^*mut*^ and control FTE organoids have similar RNA expression levels for FTE secretory cell markers (*PAX8*, *OVGP1*, and *TNF*a*IP2*) and ciliated cell markers (*FOXJ1* and *TUBB4A*), as well as the epithelial cell marker *CDH1* (Figure 4B). Additionally, both *BRCA1*^*mut*^ and control FTE organoids produced OVGP1 and TUBB4A, which were localized in the expected sub-compartments in control FTE organoids, with TUBB4A found along the lumen and OVGP1 located mostly along the basal region with a cohesive apical-to-basal polarity (Figure 4C; Figure S4B). Notably, control FTE organoids reached full maturation and formed clear cilia with the expression of its specific markers FOXJ1 and TUBB4A (Figures S4B and S4C). In contrast, proteins were distributed throughout the *BRCA1*^*mut*^-FTE organoids, indicating a loss of cellular identity and cohesive apical-to-basal polarity (Figure 4C; Figure S4D). RNA sequencing (RNA-seq) analysis of long-term cultures with gene set enrichment analysis (GSEA) and ingenuity pathways analysis (IPA) explored the molecular basis for STIC pathology in *BRCA1*^*mut*^-FTE organoids. Several ovarian cancer gene sets, including BUB1, CCNA2, CCNB2, CDC2, CDC20, CENPE, CENPF, ESPL1, HMMR, and *HPN*, as well as gene sets of the cell proliferation marker *MKi76* and proliferating cell nuclear antigen (*PCNA*), were significantly upregulated in *BRCA1*^*mut*^-FTE organoids compared with those in controls (Figure 4D; Figure S5; Table S1; Adib et al., 2004; Buttermore et al., 2017; Cahill et al., 1998; Feng et al., 2019; Li et al., 2018; Sun et al., 2017). Similarly, IPA showed that cancer-related genes, including epithelial neoplasm and ovarian cancer, were enriched in *BRCA1*^*mut*^-FTE organoids (Table S2). Together, these data demonstrate that *BRCA1*^*mut*^-FTE organoids at 4 months displayed many features similar to STIC, suggesting that they recapitulate *BRCA1*^*mut*^-related ovarian cancer initiation.

### *BRCA1^mut^*-FTE organoids recapitulate ovarian carcinogenesis

**Figure 5.**
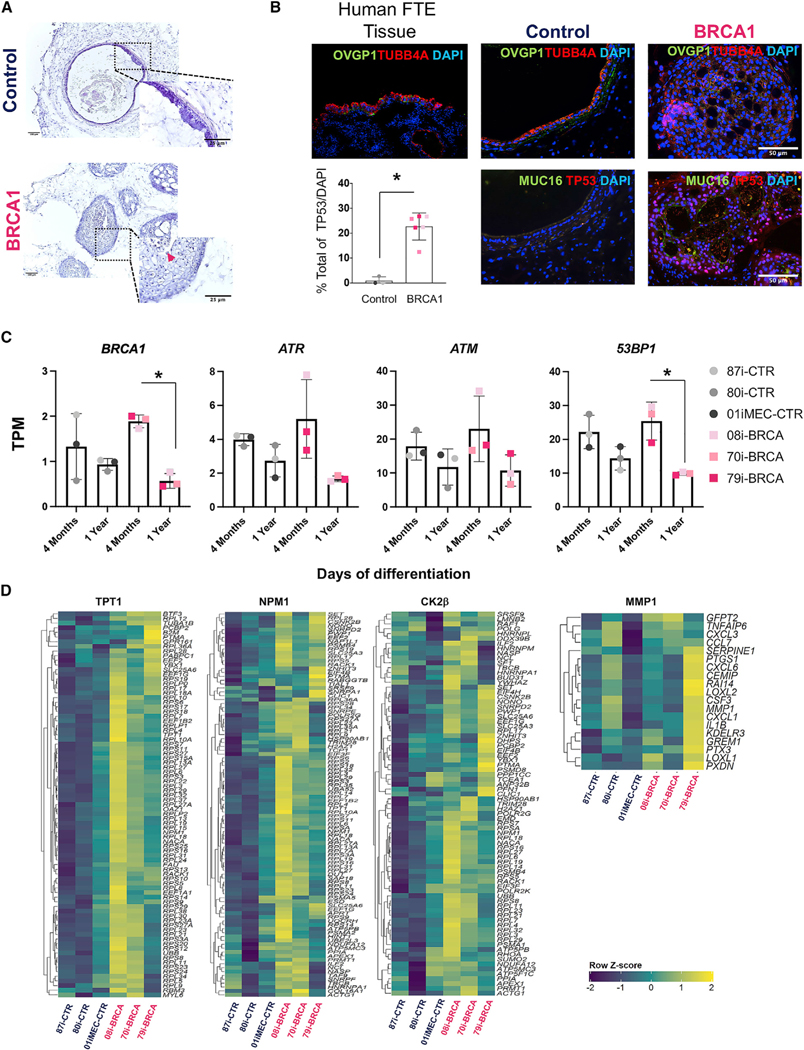
*BRCA1*^*mut*^-iPSC-derived FTE organoids reveal ovarian cancer progression of carcinogenesis *in vitro* (A) H&E images for control (80i-CTR) and *BRCA1*^*mut*^*-*FTE organoids (79i-BRCA) at 1 year in culture. (B) Immunocytochemistry of FTE markers OVGP1 and TUBB4A in human FTE tissue and control and *BRCA1*^*mut*^*-*FTE organoids. MUC16 and TP53 are shown as markers for neoplastic transformation in control and *BRCA1*^*mut*^*-*FTE organoids at 1 year in culture. Scale bars, 50 μm. Percentage of TP53 was calculated for n = 3 different controls and n = 3 different *BRCA1*^*mut*^*-*FTE organoids. (C) Expression of *BRCA1*, *ATM*, *ATR*, and *53BP1* genes at 4 months and 1 year in culture in control and *BRCA1*^*mut*^-FTE organoids. Significance was calculated using Student’s t test; *p < 0.05. (D) Differential gene expression and GSEA for control and *BRCA1*^*mut*^*-*FTE organoids at 1 year in culture. FDR value is <0.05. *Z* scores of the leading-edge genes were calculated and used to construct a heatmap.

To determine whether the precancerous lesions progress to neoplastic transformation, *in vitro* cultures were maintained to 1 year and phenotypic and molecular characteristics were evaluated. The *BRCA1*^*mut*^-FTE organoids showed enhanced structural abnormalities with increased numbers of epithelial cells, which exhibited severe dysplastic phenotypes and evidence of transformation from a single epithelial layer to a disorganized multilayer epithelium that populated the luminal space (Figure 5A). Some cells in culture sloughed off from the surface of the lumen (Figure 5A, arrowhead), suggesting that the *BRCA1*^*mut*^ epithelial cells may fail to undergo anoikis upon loss of contact with the basement membrane. Although the epithelial structures in control FTE organoids were very similar to normal human FTE tissues, *BRCA1*^*mut*^-FTE organoids lost normal epithelial structure and cellular identity (Figure 5B). Increased nuclear TP53 accumulation was observed in many epithelial cells from *BRCA1*^*mut*^-FTE organoids (Figure 5B). Collectively, these findings suggest that *BRCA1*^*mut*^-FTE organoids undergo neoplastic conversion over time.

*BRCA1*^*mut*^ carriers often show a loss of the wild-type allele in the development of primary cancers, suggesting that the reduction to homozygosity and loss of function are prerequisites of cancer development (Cancer Genome Atlas Research Network, 2011; Esteller et al., 2000; Futreal et al., 1994; Russell et al., 2000). Reports suggest that the loss of heterozygosity (LOH) in the *BRCA1* gene and DNA damage response (DDR) deficiency are strongly linked to the development of ovarian cancer (Bouwman et al., 2010; Gee et al., 2018; Weber and Ryan, 2015). To further evaluate the status of *BRCA1* activity during neoplastic development in 1-year-old and 4-month-old *BRCA1*^*mut*^-FTE organoids, gene expression profiles were analyzed for *BRCA1* and the key mediators of DDR including *ATM* (Ataxia Telangiectasia Mutated) kinase, *ATR* (ataxia-telangiectasia- and Rad3-related) kinase, and the nonhomologous end-joining factor *53BP1* (TP53 Binding Protein 1) (Bouwman et al., 2010; Hong et al., 2012; Weber and Ryan, 2015). In *BRCA1*^*mut*^-FTE organoids, but not control organoids, *BRCA1* expression was significantly downregulated in 1-year cultures compared to 4-month cultures (Figure 5C), suggesting that there has been a loss of wild-type BRCA1 activity over time. Also, *53BP1* expression was significantly downregulated over time in *BRCA1*^*mut*^-FTE organoid cultures, and *ATM* and *ATR* kinases showed a trend toward downregulation (Figure 5C), suggesting that the functional loss or deregulation of these key proteins involved in the DDR may contribute to the progression of neoplastic transformation in *BRCA1*^*mut*^-FTE organoids.

Several mechanisms exist for the loss of function of the *BRCA1* wild-type allele in heterozygous *BRCA1*^*mut*^ carriers, including LOH, methylation, and somatic coding mutation. Loss of exon 11 is one of the common genomic alterations resulting in LOH in the *BRCA1* gene. In order to evaluate whether the decrease in *BRCA1* expression in 1-year organoids resulted from LOH, qRT-PCR analysis was performed to evaluate the allele-specific transcript expression in exon 11. Although full-length *BRCA1* gene expression was significantly downregulated in *BRCA1*^*mut*^-FTE organoids at 1 year of culture, there was no evidence of changes in Delta Exon 11 expression (Figures S6A and S6B). The qRT-PCR data were confirmed with differential exon use analysis in bulk RNA-seq data, which showed no significant differential exon use at the *BRCA1* locus detected between *BRCA1*^*mut*^ and control organoids (Figure S6C). The presence of wild-type BRCA1 in *BRCA1*^*mut*^-FTE organoids suggests that downregulation of *BRCA1* expression is not due to genomic deletion leading to changes in transcript expression.

RNA-seq and subsequent GSEA analysis showed that ovarian-cancer-related modules that promote cell migration, invasion, and malignant transformation including *TPT1*, *NPM1*, *RAN*, and predictive markers such as *CK2β* and *PSME1* were significantly upregulated in *BRCA1*^*mut*^-FTE organoids, further suggesting neoplastic progression *in vitro* (Figure 5D; Figure S6D; Table S3; Chen et al., 2015; Liu et al., 2016; Londero et al., 2014; Ma et al., 2017; Wong et al., 2001; Zaoui et al., 2019). Thus, although 4-month organoid cultures exhibited a benign characteristic without evidence of malignant progression, the 1-year organoid cultures evolved into models that showed a malignant RNA profile. Strikingly, *BRCA1*^*mut*^-FTE organoids from the most aggressive case (79i-BRCA) exhibited upregulation of the MMP1 (Matrix Metallopeptidase 1) module, which may be critically involved in metastatic dissemination (Figure 5D; Behrens et al., 2001). Finally, IPA showed that cancer-related gene sets including epithelial neoplasm and malignant tumor were also highly enriched in 1-year cultures of *BRCA1*^*mut*^-FTE organoids compared to controls (Table S4).

**Figure 6.**
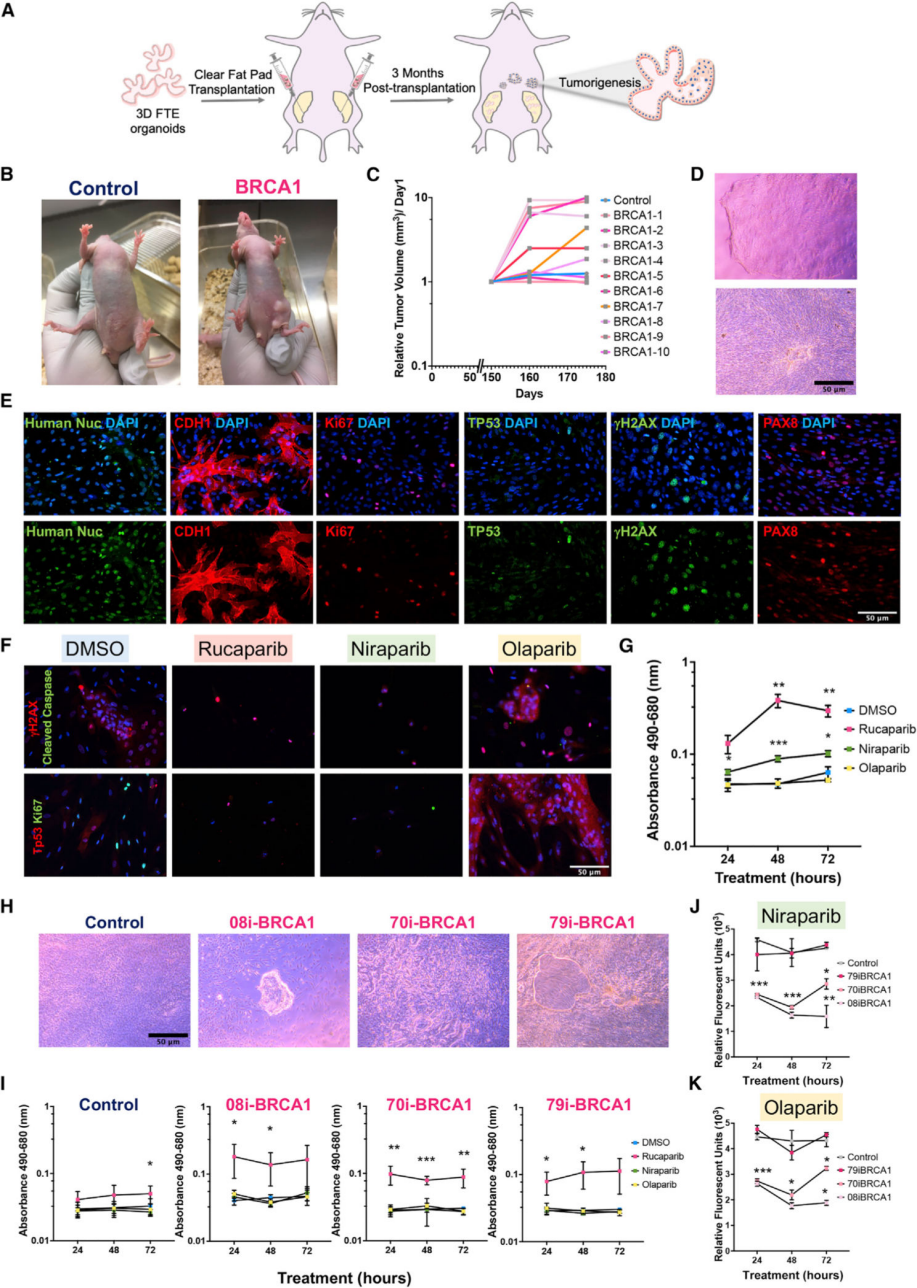
BRCA1^mut^-iPSC-derived FTE organoids show malignant characteristics of the BRCA1^mut^ lesions *in vivo* (A) Schematic of organoid transplantation into mouse fat pads. (B) Mice at 5 months post-transplantation of control-FTE organoids (87i-CTR line) and *BRCA1*^*mut*^-FTE organoids (79i-BRCA line). (C) Control and *BRCA1*^*mut*^ line tumor growth rates. (D) Brightfield images of primary cells from *BRCA1*^*mut*^ tumor lesions. (E) Immunocytochemistry of primary cells from *BRCA1*^*mut*^ tumor lesions shows cancer markers CDH1, PAX8, TP53, and Ki67; double-stranded DNA break marker γH2AX; and DAPI nuclei stain. (F) Immunocytochemistry for TP53, Ki67, γH2AX, and cleaved Caspase 3 in primary cells from *BRCA1*^*mut*^ tumor lesions treated for 72 h with 100 μM rucaparib, niraparib, olaparib, or DMSO, with DAPI nuclei stain. (G) Cellular cytotoxicity assay (LDH assay) of primary cells from *BRCA1*^*mut*^ tumor lesions over 72 h in response to 100 μM rucaparib, niraparib, or olaparib or DMSO. (H) Brightfield images of primary cells from 8-months-old control and *BRCA1*^*mut*^-FTE organoids (87i-CTR, 08i-BRCA, 70i-BRCA, and 79i-BRCA). (I–K) (I) Time course of LDH assay in response to 100 μM rucaparib treatment of primary cells from 8-monthsold control and *BRCA1*^*mut*^-FTE organoids (87i-CTR, 08i-BRCA, 70i-BRCA, and 79i-BRCA). PrestoBlue cell viability assay for primary cells from 8-monthold control and *BRCA1*^*mut*^-FTE organoids in response to 100 μM. (J) niraparib and (K) olaparib. Scale bars, 50 μm. Significance was calculated using 2-way ANOVA, Dunnett multiple comparisons;*p < 0.05, **p < 0.01, ***p < 0.001. Error bars are SD (n = 3 independent biological experiments).

Finally, in order to establish whether *BRCA1*^*mut*^-FTE organoids underwent neoplastic development *in vivo*, *BRCA1*^*mut*^ and control-FTE organoids were differentiated *in vitro* for 1 month and implanted into NU/J athymic nude mouse mammary fad pad (MFP) at three different sites (Figure 6A; Lawson et al., 2015; Zhang et al., 2019). At 3 months post-transplantation, the fat pads were extracted for macroscopic histopathological examination. Both control and *BRCA1*^*mut*^-organoids remained structurally normal in the mammary gland (Figure S7A, red arrows). However, nodules were found outside of the mammary gland, and they exhibited severe cellular abnormalities reminiscent of precancerous and cancerous characteristics only in the *BRCA1*^*mut*^ lines but not control lines (Figures S7B and S7C). H&E analysis suggested that these nodules contained both cancer and precancerous lesion formations (Figure S7C). Notably, immunohistochemical analysis demonstrated that unlike nodules from control-FTE organoids, *BRCA1*^*mut*^-FTE organoids showed substantial nuclear TP53 accumulation, which is the hallmark of an early time point in ovarian cancer development (Figure S7C). To further explore neoplastic transformation of *BRCA1*^*mut*^-FTE organoids in mice, the *in vivo* study was expanded to 6 months post-transplantation. Following transplantation of control and *BRCA1*^*mut*^-FTE organoids grown for 1 month *in vitro*, mice receiving *BRCA1*^*mut*^-FTE organoids developed several visible external tumors (Figure 6B). The relative tumor growth rate was determined and most visible *BRCA1*^*mut*^ tumor lesions showed a significant growth rate compared to only one visible lesion in controls that did not show any growth (Figure 6C). To determine whether *BRCA1*^*mut*^*-*FTE organoid-derived tumor lesions have neoplastic transformation features, control and *BRCA1*^*mut*^ tumor lesions were dissociated and primary cells were obtained. Primary cells from the control transplants did not survive (data not shown); however, primary cells from *BRCA1*^*mut*^ tumor lesions have the ability to grow in a multilayer (Figure 6D) and exhibited clear production of FTE markers and cancer markers, including CDH1, PAX8, and Ki67, and increases in the frequency of double-stranded DNA break, shown with γH2AX accumulation and heterogenous TP53 staining, which is compatible with a wild-type pattern of TP53 expression (Figure 6E). Human nuclei marker production confirmed that primary cells from *BRCA1*^*mut*^ tumor lesions were human epithelial cells (Figure 6E).

To next explore BRCA1^mut^-related cancer characteristics, we investigated sensitivity to Poly (ADP-ribose) polymerase inhibitors (PARPis), which induce synthetic lethality in the context of HR deficiencies due to BRCA1^mut^ (LaFargue et al., 2019; Lord and Ashworth, 2012, 2017). Federal Drug Administration-approved PARPi drugs used to treat epithelial ovarian cancer (olaparib, niraparib, and rucaparib) were evaluated in primary, monolayer cultures of 79i-BRCA cells derived from the mouse tumors. In response to rucaparib and niraparib treatment, primary cells showed significant DNA damage accumulation, with high γH2AX nuclear localization, compared to the control treatment (Figures 6F–6K), as well as elevated cell death demonstrated by TP53 and cleaved Caspase3 production and reduced levels of the cell proliferation marker Ki67 (Figure 6F; Figure S7D), and increased cellular cytotoxicity demonstrated by a lactate de-hydrogenase (LDH) assay (Figures 6G and S7E). Although olaparib did not appear to cause cellular cytotoxicity, it reduced cellular proliferation over time (Figures 6F and S7D).

To further explore the mechanism of PARPi actions on different *BRCA1*^*mut*^ lines, primary cells dissociated from 8-month-old *BRCA1*^*mut*^-FTE and control organoids were treated with PARPi followed by cytotoxicity and cell viability assays (Figure 6H). Rucaparib treatment led to significant cell death in all *BRCA1*^*mut*^ cells compared to the control treatment (Figure 6I). Niraparib and olaparib treatment reduced cellular viability at various levels, with a significant reduction in viability in the 08i-BRCA line, an initial significant change in the 70i-BRCA line, and no change in the 79i-BRCA line (Figures 6J and 6K). Collectively, these data show that *BRCA1*^*mut*^-FTE organoids exhibit severe cellular abnormalities, induced neoplastic transformation *in vitro* and *in vivo*, and recapitulated ovarian carcinogenesis. Importantly, this 3D human organoid model provides a platform to study cancer drug efficacy and specificity.

## DISCUSSION

HGSC is the most common form of epithelial ovarian cancer, with a 5-year survival rate of ∼90% if detected at stage I. Unfortunately, no clinical biomarkers exist to reliably detect stage I/II HGSC, and there is a wide range of clinical presentation and behavior in *BRCA1*^*mut*^ carriers and other high-risk populations. To address these clinical challenges, it is essential to better understand and model key molecular events involved in *BRCA1*-related ovarian cancer initiation and progression to improve predictive outcomes and interventions.

Current models that use established cell lines (normal and malignant) to study *BRCA1* function are limited, as they do not reflect genetic characteristics of specific patients, nor do they recapitulate the complexity of the affected tissue or the dynamic changes that occur in disease initiation and progression. Recent studies have suggested that 3D cell culture systems better reflect the tissue and cell microenvironment than 2D models, and the behaviors of 3D-cultured cells are thought to recapitulate *in vivo* cellular responses. Several patient-biopsy-derived models and mouse 3D ovarian and other cancer models have been established and are shown to resemble the tumor structure *in vitro* (Crespo et al., 2017; Dumont et al., 2019; Kopper et al., 2019; Kurman, 2013; Lõhmussaar et al., 2020). Although biopsy-derived 3D organoids provide a drug screening platform and can be used for personalized therapy, this cell source cannot address how *BRCA1*^*mut*^ leads to ovarian cancer initiation at the FTE and contributes to transformation over time. Finally, biopsy-derived organoids with introduced oncogenes and/or tumor suppressor mutations to induce cancer initiation fail to reveal critical molecular changes involved in *BRCA1*-related FTE transformation to ovarian cancer and cannot be used to improve early detection (Kopper et al., 2019; Lõhmussaar et al., 2020). Clearly, a better understanding of the initiation and early development of HGSC requires more relevant *in vitro* human models.

Here, we generated *BRCA1*^*mut*^ iPSC lines that retained a pluripotent state, did not develop chromosomal abnormalities, and could be differentiated into the three germ layers, as shown in previous reports (Silva et al., 2021a; Soyombo et al., 2013). We differentiated control and *BRCA1*^*mut*^ iPSCs into 3D FTE organoids that consisted of both the CDH1-positive epithelial layer and CDH1-negative fibroblast-like cells. We were surprised to find that iPSC-derived *BRCA1*^*mut*^ lines, but not controls, generated FTE organoids with STIC histological characteristics such as cellular crowding, loss of polarity, and severe nuclear and cellular atypia. They also showed biomarker expression such as Ki67, TP53, and CA125/MUC16 that are common to ovarian cancer (Bast et al., 2005; Felder et al., 2014; Kuhn et al., 2012). Although *BRCA1* expression was significantly downregulated in 1-year cultures compared to that of 4-month cultures, the data show that the mechanism for this ovarian cancer model is not based on LOH. Similarily, LOH was not observed following teratoma formation of BRCA1 iPSCs (Soyombo et al., 2013). Other mechanisms are currently being assessed with whole-genome and epigenetic sequencing.

Furthermore, the GSEA analysis indicated that *BRCA1*^*mut*^*-*FTE organoids cultured for 4 months retain highly similar key molecular signatures of ovarian cancer. For instance, hyaluronan-mediated motility receptor (HMMR), which is involved in microtubule spindle assembly and cell cycle progression, was one of the upregulated pathways (Buttermore et al., 2017; Maxwell et al., 2005, 2011). *BRCA1* binds to HMMR and induces its degradation in normal cells and thus regulates apicobasal polarization of the breast luminal epithelium (Maxwell et al., 2011). Therefore, the loss of *BRCA1* function linked to high HMMR expression can contribute to cancer progression (Anttila et al., 2000; Blanco et al., 2015; Maxwell et al., 2011). Similarly, elevated HMMR expression is associated with poor prognosis for *BRCA1*^*mut*^ breast cancer (Blanco et al., 2015; Maxwell et al., 2011; Wang et al., 2014). Additionally, the expression of the potential PAX8 regulator lncRNA, namely PAX8-AS1 (PAX8 Antisense RNA 1), was significantly differentially expressed at the early time point for all the *BRCA1*^*mut*^ lines (data not shown). Our data suggest that deregulation of PAX8 expression, which is the hallmark of ovarian cancer, is critical for ovarian cancer initiation and progression (Bowen et al., 2007). Therefore, *BRCA1*^*mut*^*-*FTE organoids at 4 months in culture recapitulate an early time point in ovarian cancer development that may reflect the neoplastic behavior of the original patient source and could provide a tool to identify genes for early detection. This platform can also benefit the development of new therapeutic approaches.

The FTE consists of two distinct cell populations, namely, ciliated columnar cells (FOXJ1+, TUBB4A+) and secretory cells (PAX8+, OVGP1+), and they share common progenitors. Balancing these cell populations along the FTE is critical for maintaining epithelial homeostasis. The FTE in HGSC shows reduced ciliated epithelial cells and increased PAX8-positive secretory cells, a phenotype that is considered a potential biomarker for the early stages of serous carcinogenesis (Coan et al., 2018; Tao et al., 2020; Wang et al., 2015). However, how these two cell populations shift during cancer initiation and progression is unknown. We demonstrated that control FTE cells consisted of distinct ciliated and secretory cell populations and reached full maturation by forming cilia, similar to normal FTE tissue. In contrast, early-stage *BRCA1*^*mut*^-FTE cells expressed both secretory cell markers (PAX8 and OVGP1) and TUBB4A in the same cells, which recapitulates the loss of cellular identity seen in HGSC fallopian tubes and suggests that this loss could be an early precancerous event.

We found that *BRCA1*^*mu*t^-FTE organoids exhibited structural abnormalities with two distinct morphologies in early time points, which we considered as a precancerous time. We classified these abnormalities as Type I with a micropapillary-like structure or Type II with increased cell layers and nuclear stratification. STIC morphology displays the multilayered epithelium with stratification, loss of polarity, and absence of cilia, similar to Type II. Conversely, micropapillary structure formation is reported in HGSC, specifically in p53 wild-type cancer (Chui et al., 2021). Although we do not have further evidence that a primary structure predominates for neoplastic transformation, both types could result in neoplastic transformation.

Exploring carcinogenesis in this *in vitro* model revealed a strong correlation between disease severity in the BRCA patient and the phenotype in the organoids. Notably, organoids derived from the 79i-BRCA iPSC line (patient with stage IIIC ovarian cancer at age 31.4) exhibited a more aggressive molecular signature than organoids derived from the 08i-BRCA (patient with stage IIC ovarian cancer at age 36.3) or 70i-BRCA (patient with stage IIIC ovarian cancer at age 33.6) lines, suggesting that they faithfully recapitulated *BRCA1*^*mut*^-related phenotypes. Importantly, the metastatic feature of the *BRCA1*^*mut*^*-*FTE organoid was confirmed after transplantation into immunocompromised mice where further maturity of the cancer occurred, thus providing a physiologically relevant platform of induced cancer initiation in a short period. It appears that iPSC modeling predicts the severity of disease in these *BRCA1* patients, although more cases are needed to confirm the phenotypic relationship between the severity of the organoid model and clinical outcomes.

PARPi therapy is as an exciting targeted strategy in *BRCA*-deficient ovarian cancers (Lord and Ashworth, 2012, 2017). PARPi inhibition induces DNA damage accumulation, activating DDR pathways that orchestrate cell fate decisions, including transient or prolonged cell proliferation arrest or apoptosis. In women with a BRCA germline and/or deficient and platinum-sensitive ovarian cancer, PARPi-targeted therapy increased rates of cancer-free survival. Unfortunately, not all patients respond to PARPi, and many of those patients who initially respond develop resistance. Here, we demonstrated *BRCA1*^*mut*^-dependent responses to different PARPi treatments. The efficacy and specificity of PARPi varied based on the *BRCA1*^*mut*^. Although rucaparib induced cell death specific to *BRCA1*^*mut*^ cells, olparib and niraparib led to reduced cell proliferation at various levels across all lines. Our data suggest that this model may be used to study PARPi efficacy on different *BRCA1*^*mut*^ with unique genetic backgrounds.

We established that *BRCA1*^*mut*^-FTE, but not control, organoids showed evidence of neoplastic development *in vivo*. Transplantation into mouse MFP has been shown to provide a similar hormonal microenvironment to the ovaries (Zhang et al., 2019). Interestingly, after engraftment into the MFP, *BRCA1*^*mut*^ organoids formed subcutaneous nodules that exhibited cellular abnormalities with precancerous and cancerous characteristics. This finding was not observed with control organoids. It is unknown why the nodules formed outside the MFP. Although this result was unexpected, it was consistent throughout our studies and has been reported for xenografted human breast cancer cell lines that were found to be distinct from the mouse MFP (Samineni et al., 2011). Presumably subcutaneous regions provide a conducive microenvironment for neoplastic growth. MFP injection with a subsequent visualization of subcutaneous nodules permits tumor growth assessment over time *in vivo*, which could be key for drug screening.

The tumor microenvironment is composed of several cell types, including tumor-associated stromal cells, immune cells, and endothelial cells, as well as non-cellular components including the extracellular matrix (ECM) and other soluble factors (Cho et al., 2015; Jin and Jin, 2020). The role of the tumor microenvironment in facilitating cancer progression is gaining recognition (Jin and Jin, 2020), although a molecular understanding of the tumor microenvironment role in promoting neoplastic progression remains unclear. Our 3D model could provide a platform to study the contribution of the tumor microenvironment to neoplastic progression.

In summary, this 3D platform provides a more faithful physiological model that enables a deeper investigation into signaling pathways in early ovarian cancer initiation in both a tissue- and patient-specific context. This model offers a promising, biologically relevant platform to validate new drugs and targets for a cancer that is not well understood. By generation of 3D organoid models from various *BRCA1*^*mut*^ patients, *BRCA1*^*mut*^-dependent responses to treatment can be elucidated and the information can help predict the drug sensitivity of individual patients based on their unique genetics. This iPSC-based BRCA model can provide a basis for personalized early detection and preventative strategies for women carrying *BRCA1*^*mut*^ and potentially other high-risk mutations.

## LIMITATIONS OF THE STUDY

This research is subject to some limitations. Although this iPSC-based BRCA model clearly shows abnormal cells in masses, it is still an *in vitro* model that does not fully recapitulate all *in vivo* histological features of HGSC (ovarian cancer) seen in patients. As an artificial culture environment does not include the hormonal environment and cell type heterogeneity of tissues *in vivo*, it may not reflect the entire biology of *BRCA1*-initiated cancer progression. Finally, although iPSC-derived FTE from *BCRA1* patients clearly shows cancer initiation and early signs of progression, the validity of this model for later stages of cancer needs to be validated in further studies.

## STAR★METHODS

### RESOURCE AVAILABILITY

#### Lead contact

Further information and requests for resources and reagents should be directed to and will be fulfilled by the lead contact, Clive Svendsen: Clive.Svendsen@cshs.org.

#### Materials availability

iPSC lines are available from Cedar-Sinai Biomanufacturing Center iPSC Core. Other materials generated in this study are available upon request.

#### Data and code availability

RNA-seq data have been deposited at GEO (GEO: GSE190134) and are publicly available as of publication date. Code used to generate data for this study will be available and shared by lead contact upon request. Original western blot images and microscopy data reported in this paper will be shared by the lead contact upon request. Any additional information required to reanalyze the data reported in this paper is available and will be shared by the lead contact upon request.

### EXPERIMENTAL MODEL AND SUBJECT DETAILS

#### Cell Lines

All iPSC lines were generated at the iPSC Core at Cedars-Sinai Medical Center. Parent cells were transfected with non-integrating episomal plasmid expressing 7 factors: *OCT4, SOX2*, *KLF4*, *L-MYC*, *LIN28*, *SV40LT* and *p53* shRNA (pEP4 E02S ET2K, pCXLE-hOCT3/4-shp53-F, pCXLE-hUL, and pCXLE-hSK). Details regarding the disease status, sex, age at collection, and *BRCA1* genotype for each iPSC line are provided in Table 1. All the cell lines and protocols in the present study were used in accordance with the guidelines approved by the stem cell research oversight committee (SCRO) and institutional review board (IRB) under the auspice IRB-SCRO Protocols Pro00032834 (iPSC Core Repository and Stem Cell Program) and Pro00021505 (Svendsen Stem Cell Program).

We selected 3 existing female control iPSC lines from the Cedars-Sinai Biomanufacturing Center iPSC Core. Control iPSC lines were reprogrammed from healthy female donor cells with peripheral blood mononuclear cells (PBMCs) used for CS80i-CTR-Tn3, lymphoblastoid cells (LCLs) used for CS87i-CTR-n3 and mammary epithelial cells used for CS01iMEC-CTR-n4. 3 female *BRCA1*^*mut*^ patients’ LCLs were chosen from the Gilda Radner Hereditary Cancer Program repository and *BRCA1*^*mut*^ iPSC lines (Lines CS08i-BRCA-n5, CS70i-BRCA-n1 and CS79i-BRCA-n2) were generated at Cedars-Sinai Biomanufacturing Center iPSC Core.

*BRCA1*^*mut*^ iPSC lines were confirmed by the presence of *BRCA1* heterozygous mutations based on DNA sequencing analysis. Results showed that each patient had a different heterozygous mutation - 79i-BRCA (IVGS5+1G > A, located at the junction between exon 5 and intron 6), 70i-BRCA (1048delA), and 08i-BRCA (3875del4, located in exon 11). These mutation matched to each patient’s clinical diagnosis.

#### Animals

Foxn1^nu^, formerly Hfh11^nu^ (known as NU/J athymic nude) female mice at postnatal day 24 were purchased from the Jackson Laboratory and both control and *BRCA1*^*mut*^-FTE organoids were injected. All the animals maintained at vivarium facilities at the Cedars-Sinai Medical Center under standard conditions with the light on 12 hours and off 12 hours and *ad libitum* access to standard chow and water. All animal procedures were performed following the NIH Guide for the Care and Use of Laboratory Animals and approved by the Institutional Animal Care and Use Committee (IACUC) of Cedars-Sinai Medical Center (protocol #005318).

### METHOD DETAILS

#### Ethics Statement

All human samples were obtained using the approved IRB in accordance with relevant guidelines and regulations under IRB PRO00033469 protocol number. Informed consent was acquired from all human subjects and/or their legal guardians. Human B cells, mammary epithelial cells, and fibroblast cells were obtained from the Institute for Medical Research. The Cell Repository maintains the informed consent and privacy of the donor. All the cell lines and protocols in the present study were used in accordance with the guidelines approved by the stem cell research oversight committee (SCRO) and institutional review board (IRB) under the auspice IRB-SCRO Protocols Pro00032834 (iPSC Core Repository and Stem Cell Program) and Pro00021505 (Svendsen Stem Cell Program).

#### Generation and culture of iPSCs

Human iPSC lines were generated based on a previously published protocol (Barrett et al., 2014). The starting cell source for control lines was healthy donor female cells, with PBMCs used to generate control lines CS80i-CTR-Tn3, lymphoblastoid cells (LCLs) used to generate CS87i-CTR-n3 and mammary epithelial cells (from a mammary epithelium biopsy) used to generate CS01iMEC-CTR-n4. LCLs from ovarian cancer patients with *BRCA1* mutations were used to generate lines CS08i-BRCA-n5, CS70i-BRCA-n1 and CS79i-BRCA-n2. Briefly, cells were cultured at 37°C and 5% CO_2_ in a humidified incubator, with LCLs maintained in RPMI 1640 (Life Technologies) media supplemented with 15% fetal bovine serum (FBS) and 2 mM L-glutamine, and mammary epithelial cells maintained in MammaryLife™ basal media (Life Line Cell Technology). LCLs and mammary epithelial cells were reprogrammed into virus-free iPSC lines with B cell Nucleofector Kit (VPA-1001, Lonza) using 1.5 mg of each episomal plasmid (Addgene) expressing 7 factors: *OCT4, SOX2*, *KLF4*, *L-MYC*, *LIN28*, *SV40LT* and *p53* shRNA (pEP4 E02S ET2K, pCXLE-hOCT3/4-shp53-F, pCXLE-hUL, and pCXLE-hSK). Nucleofected cells were plated on feeder-independent BD Matrigel growth factor-reduced Matrix (Corning/BD Biosciences, #354230). All cultures were maintained at 20% O_2_ during the reprogramming process. Cells were initially cultured in their maintenance medium for 3 days post-nucleofection and gradually transitioned to reprogramming media (RM) by adding 1 mL RM to the original media daily for the next 3 days to aid in LCL attachment. Reprogramming media contains DMEM/F12, 1% NEAA, 1% GlutaMax, 1% N2, 2% B27, 0.5% Antibiotic-Antimycotic (GIBCO #15240–062), 0.1 μM β-mercaptoethanol, 100ng/ml bFGF (PeproTech), 1:1000 (∼1000 units) hLIF (Millipore, #LIF1010), 0.5 μM PD0325901 (Cayman Chemicals, #13034), 3 μM CHIR99021 (Tocris, #4423), 10 μM HA-100 (Santa Cruz Biotech, #203072), and 0.5 μM A-83–01 (Tocris, #2939). Cells were maintained in RM for 15 days with fresh media replenishment every other day. They were then gradually changed to chemically-defined mTeSR®1 medium (STEMCELL) between 17–20 days post-nucleofection. Colonies with iPSC-like morphology were mechanically isolated, transferred onto 12-well plates with fresh Matrigel Matrix, and maintained in mTeSR®1 medium on growth factor-reduced Matrigel Matrix (BD Biosciences)-coated plates at 37°C in a 5% CO_2_ incubator. For weekly passaging, colonies were lifted carefully with a cell scraper, removed using a 5 mL glass pipette, and replated at a 1:6 ratio.

#### Directed differentiation of iPSCs into 3D human FTE organoids

Human iPSCs were split onto Matrigel-coated plates and cultured in mTeSR®1 medium until 80% confluent. Cells were exposed to 100 ng/ml human recombinant activin A (Stemgent) and 3 μM CHIR99021 (Cayman Chemicals) to differentiate toward mesoderm, and cultured in DMEM/F12 (GIBCO) + Glutamax (Invitrogen) supplemented with 500 U/ml penicillin streptomycin (GIBCO) and 2% FBS with addition of 10 μM ROCK inhibitor Y-27632 (Stemgent) for 2 days. To differentiate cells toward intermeditae mesoderm (IM), media was changed to DMEM/F12 (GIBCO) + Glutamax (Invitrogen) supplemented with 0.1 mM non-essential amino acids (Invitrogen), 500 μ/ml penicillin/streptomycin (GIBCO), 0.55 mM 2-mercaptoethanol, 10% KOSR (Invitrogen), 100 ng/ml BMP4 (R&D Systems), 3 μCHIR99021 (Tocris), and 10 μM ROCK inhibitor Y-27632 (Stemgent) for 2 days. Spheroids were collected on day 4 from every well under a stereomicroscope using a 200 μL barrier pipette tip and pooled into a 1.5 mL microcentrifuge tube. Spheroids were then mixed with 50 μL Matrigel (BD Biosciences) and slowly pipetted into the middle of one well of a 24-well Nunclon Delta surface dish. The 3D droplet was allowed to solidify for 10–15 minutes in a tissue incubator, and Matrigel beads were then bathed in new fallopian tube media (nFTM) containing advanced Dulbecco’s modified Eagle medium/F12 supplemented with 12 mmol/L HEPES, 1 X Glutamax, 2% B27, 1% N2 (all from Life Technologies, Carlsbad, CA), and was supplemented with 10 ng/mL murine recombinant EGF (PeproTech), 10 ng/mL basic FGF, 30 ng/ml human Noggin (R&D system), 0.5 mM TGF-b R Kinase Inhibitor IV (SB431542, Calbiochem) and 1% penicillin/ streptomycin (Invitrogen). For FTE differentiation, 100 ng/ml human recombinant WNT4 (R&D Systems) with 10 μM ROCK inhibitor Y-27632 (Stemgent) were added for two days and followed by 20 ng/ml human recombinant Follistatin (PeproTech) with 10 μM ROCK inhibitor Y-27632 (Stemgent) for next two days. Media was replaced every 3–4 days as necessary, and cells were replated every two weeks. All the cultures were tested for mycoplasma contamination monthly.

#### Western blot analysis

iPSCs were treated with the proteasome inhibitor MG132 for 24 hours, and treated and nontreated cells were gently scraped off the plates, washed with phosphate buffered saline (PBS), and centrifuged at 15000 RPM for 1 min. Samples were lysed using 1X NETN buffer (20 mM Tris-HCl (pH 8.0), 100 mM NaCl, 0.5 mM EDTA and 0.5% NP-40) supplemented with phosphatase/protease inhibitor cocktail (MS-SAFE, Sigma-Aldrich). Lysates were sonicated at a frequency of 20 kHz for 5 s (3times) on ice and incubated on ice for 20 min. Samples were centrifuged for 20 min at 4°C at 15000 RPM. Total soluble protein concentrations were measured using a Bradford assay (BIO-RAD). 4X Laemmli sample buffer (BIO-RAD 161–0774) was added to 100 μg of total protein extracts and samples were boiled for 5 min. Samples were run in 4%–20% Mini-PROTEAN TGX Precast gels (BIO-RAD, 456–1094) and transferred to nitro-cellulose membrane using 1X transfer buffer (25mM Tris, 190 mM glycine, 20% Methanol and 0.1% SDS) over night at 4°C. Membranes were blocked with Odyssey blocking buffer (LI-COR) and then incubated with primary BRCA1 antibody (Millipore, OP92), which is raised against the N-terminal region of BRCA1 protein, overnight at 4°C. Following incubation with dye-labeled mouse secondary antibody for 2 hours at room temperature, signals were visualized using an Odyssey Fc imaging system (LI-COR).

#### RNA Isolation and Real-time PCR Analysis

Total cellular RNA was isolated using QIAGEN RNeasy Mini kit following manufacturer recommendations (QIAGEN). RNeasy-treated total RNA (1 μg) was used for cDNA synthesis using the Quantitect Reverse Transcription Kit for cDNA synthesis for PCR (QIAGEN). Real-time PCR was performed using the SYBR Select Master Mix (Appliedbiosystem). The levels of expression of respective genes were normalized to corresponding *GAPDH* values and shown as fold change relative to the value of the control sample. All sample analyses were carried out in triplicate. List of primers used for real-time PCR experiments are listed in Table S5. At least one set of replicates was performed blindly.

#### Immunocytochemistry for Fallopian Tube Organoid

Organoids were fixed with 4% paraformaldehyde (PFA) in PBS for 20 minutes followed by three washes (3X) with PBS. The fixed organoids were then cryoprotected in 30% sucrose at 4°C overnight and embedded in OCT (Tissue-Tek). Frozen sections were collected at 12 μm using a cryostat onto glass slides and stored at −80°C. Each section was rehydrated with PBS for 5 min, permeabilized in PBS containing 2% Triton X-100 for 10 min at room temperature, blocked in a solution of 10% Normal Donkey Serum in PBS+0.05% Triton X-100 (PBS-T) for 1 hour at room temperature, followed by 2 hours incubation at room temperature in primary antibodies in blocking solution. The following primary antibodies were used at 1:200 dilution: PAX8 (Proteintech, 21384–1-AP), TUBB4A (Abcam, ab1315), POU5F1 (Stemgent, 09–0023), Nanog (Stemgent, 09–0020), SOX2 (Stemgent, 09–0024), TRA-1–60 (Stemgent, 09–0010), TRA-1–81 (Stemgent, 09–0011), SSEA4 (Stemgent, 09–0006), CDH1 (R&D System, AF648), OVGP1 (SIGMA, HPA062205), p53 (DO-1) (Santa Cruz Biotechnology, sc-126), Ki-67 (SP6) (Invitrogen, MA5–14520) MUC-16 (Sigma, HPA065600). The slides were then washed with PBS-T three times for 15 minutes each at room temperature and incubated with species-specific AF488 or AF594-conjugated secondary antibodies (Invitrogen) at 1:400 dilution, followed by 4’,6-diamidino-2-phenylindole (DAPI) (Molecular Probes, D3571) nuclear counterstain. Following three washes in PBS-T, the tissue was covered with a glass slide and imaged using Nikon/Leica microscopes. Each selected image is representative of a minimum of three independent experiments with at least two technical duplicates. At least one set of replicates was performed blindly.

#### Immunohistochemistry for TP53

Dissected tissues were fixed with 10% formalin (Fisher Chemical) for 1 hour at room temperature and then overnight at 4°C. The tissues were washed with PBS once and then were stored tissue in 70% EtOH overnight. Next day tissues were submitted to the Cedars-Sinai Pathology Core for paraffin embedding and then sectioning using a microtome. 5 mm slides were deparaffinized with xylene (3X) for 10 minutes each interval and then rehydrated in 100% EtOH (2X), 95% EtOH (2X), 75% EtOH, 50% EtOH at 5 minutes each. The slides were rinsed in tap water (2X) for 2 minutes intervals. The slides were microwaved 8 minutes at 100% power and 15 minutes at 30% power in Vector unmasking solution (Citric Acid Based, H-3300) for antigen retrieval and then cooled down to room temperature for 30 minutes. The slides were then washed with PBS and treated with 0.3% H_2_O_2_ in Methanol for peroxidase inactivation for 30 minutes at room temperature. The slides were washed with PBS (3X) with 2 minutes intervals, blocked in a solution of 3% BSA in PBS-T for 1 hour at room temperature, followed by overnight incubation at 4°C in TP53 primary antibody in 1:200 dilution in blocking solution. The slides were washed with PBS (3X) for 15 minutes each at room temperature, incubated with mouse-specific biotinylated secondary antibody for 2 hours at room temperature, washed with PBS (3X) for 15 minutes intervals. Then slides were treated with Avidin-Biotin Complex (ABC, VECTASTAIN® Elite® ABC-HRP Kit) for 30 minutes at room temperature and washed with PBS (3X) for 2 minutes intervals. The slides were then incubated with DAB solution (Vector, SK-4105) for 5 minutes and washed with distilled water (3X) for 5 minutes intervals. The slides were counterstained in hematoxylin (ImmunoMaster Hematoxylin, American MasterTech Scientific, Inc.) for 8 minutes, and then rinsed in running tap water for 5 minutes, dehydrated in 50% EtOH, 75% EtOH, 95% EtOH (2X), 100% EtOH (3X), at 1 minute each and Xylene (3X) at 5 minutes each. The tissues were covered with a glass cover slide using a mounting medium (Richard-Allan Scientific Mounting Medium).

#### H&E Staining

Organoid and fallopian tube tissues were fixed with 4% PFA in 1X PBS for 20 minutes, followed by PBS (3X) washes. The fixed organoids and fallopian tube tissues were cryopreserved in 30% sucrose at 4°C overnight and then embedded in OCT (Tissue-Tek). Frozen sections were collected at 12 μm using a cryostat onto glass slides and stored at −80°C. Each section was rehydrated with PBS for 5 minutes. The slides were stained in hematoxylin (ImmunoMaster Hematoxylin, American MasterTech Scientific, Inc.) for 8 minutes, then rinsed in running tap water for 5 minutes. The slides were then stained with eosin (Eosin Y Phloxine B, American MasterTech Scientific, Inc.) for 10 s. The slides were rinsed in tap water and dehydrated in 50% EtOH, 75% EtOH, 95% EtOH, 100% EtOH, and Xylene at 1 minute each. Tissues were covered with a glass coverslip using mounting medium (Richard-Allan Scientific Mounting Medium).

#### Organoid Thickness Measurement

Using Stereo Investigator 11.07 (MBF Bioscience), the “contour” tool was used to mark around FTE organoid sections on slides, which were then imaged at 20X magnification. The images were stitched together to yield one organoid section per file. The images were then imported into Neurolucdia 11.07 (MBF Bioscience) and FTE thickness measurements were taken using the “quick measure line” tool at increments of 200 μm along the epithelium of each imaged section for 228 organoids from 5 different biological replicates. Image analysis tool for three different control (87i-CTR, 80i-CTR, and 01iMEC-CTR) and three different *BRCA1* ^*mut*^ (08i-BRCA,70i-BRCA and 79i-BRCA) organoids at 4-month culture, with 200 μm grids used as a reference. 10 to 20 different points based on the full size of organoids were used to calculate average thickness (Figure S5A) and cellular outgrowth was calculated using the surface roughness equation as follows: $R_{a}=\frac{1}{n}\sum_{i=1}^{n}\left|y_{i}\right|$. Using the “ROI Manager” tool in ImageJ, individual cells stained with anti-Ecad were traced and analyzed to find the area of each FTE cell. Using ImageJ, the images of FTE sections were converted to binary and analyzed to find the area stained with DAPI. Using the DAPI count, the total area was divided to give us the nucleus size per FTE cell. At least one set of replicates was performed blindly.

#### Transcriptional Analysis of FTE Organoids

RNA integrity was determined on an Agilent 2100 Bioanalyzer and samples with RIN ≥ 9 were selected for cDNA library construction using Illumina TruSeq Stranded mRNA library preparation kit. Sample libraries were sequenced on an Illumina NextSeq 500 platform with 75bp single-end sequencing at the Cedars-Sinai Applied Genomics, Computation & Translational Core. Demultiplexing and conversion of raw sequencing data to FASTQ was performed with Illumina bcl2fastq software and reads were mapped to the GRCh38.p13 genome assembly (GENCODE 32) using Salmon version 1.1.0. Gene-level read counts were then quantified using the R/Bioconductor package tximport. Genes were filtered by an average read count of 3 per sample. Ensembl gene IDs without a matching HGNC symbol were also removed, resulting in 19,498 genes that met the filtering criteria. Raw counts were normalized using the variance stabilizing transformation of the DESeq2 package. Gene Set Enrichment Analysis (GSEA) was then performed using the filtered and normalized data to test enrichment in the C4: cancer gene neighborhood and cancer module gene sets from MSigDB (http://www.gsea-msigdb.org/gsea/index.jsp) using the default GSEA settings (Subramanian et al., 2005). Normalized expression values for the leading-edge subset of genes responsible for gene set enrichment were converted to Z-scores and plotted using the R/Bioconductor ComplexHeatmap package. A cutoff of FDR p value < 0.05 was used to determine which gene sets and pathways were significantly enriched in *BRCA1*^*mut*^ FTE organoids.

For differential exon usage (DEU) analysis, RNaseq reads were aligned to GENCODE (version 38) primary genome assembly using STAR aligner (version 2.7.9a) with default settings. Aligned reads overlapping unique exon bins were quantified and visualized using DEXseq (version 1.38.0) (Anders et al., 2012) with false discovery rate set at 10%. Default settings were used for all DEXseq functions except for aggregate flag set to ‘-r no’ during annotation file preparation.

#### Mammary Fat Pad (MFP) Transplantation

All animal procedures were performed following the NIH Guide for the Care and Use of Laboratory Animals and approved by the Institutional Animal Care and Use Committee (IACUC) of Cedars-Sinai Medical Center (protocol #005318). NU/J athymic nude female mice at postnatal day 24 were anesthetized by isoflurane, and surgery was performed. A 1.5 cm midline incision on the ventral surface of the skin was made beginning between the #4-mammary nipples and extending upward toward the thorax. Two contralateral incisions were prepared, beginning at the lower end of the previous incision, ending between the #4 and #5 mammary nipples, such that the incisions describe an inverted Y. The #4 nipples, the blood vessel near the inguinal lymph node in the #4 fat pad, and the blood vessel which courses between the #4 and #5 fat pads were cauterized. Since postnatal day 24, the #4 mammary gland has not grown beyond the lymph node, the triangular area described by the cautery points was surgically removed, resulting in the #4 mammary gland being “cleared.” The residual tissue called a “cleared” mammary fat pad was used to implant organoids. Meanwhile, organoids were collected in media and centrifuged, and the total packed volume of organoids was calculated. For 10 injections, 500 μL of total organoids were resuspended in 5 mL PBS, and 550 μL of organoids in PBS were relocated into the Eppendorf tube and then pelleted. The 1-month-old control and *BRCA1*^*mut*^-FTE organoids with similar sizes were resuspended in 20 μL Matrigel beads and then placed onto the cleared mammary fat pad using sterile micro-forceps. The surgery was carried out bilaterally, on both #4 mammary glands. After the surgery, the skin surrounding the incision was pinched and sutured with a silk suture, which was removed later. At 3 months and 6 months post-transplantation, animals were sacrificed, the brightfield images were taken, and nodules and fat pad where the organoids were transplanted were dissociated from the skin using scissors. At 5 months post-transplantation, tumor volume was recorded by caliper measurements using the formula (length [mm]) 3 (width [mm]) 3 (height [mm]) 3 (πX(4/3)). Relative tumor volume (RTV) was determined according to the formula RTV = Vn/V150 where V150 represents the tumor volume at day 150, which was the first measurement and Vn represents the tumor volume as measured after an interval of 10 days, respectively. Tissue was fixed in 10% formalin (Fisher Chemical) for 1 hour at room temperature and then overnight at 4°C, then dehydrated with 70% ethanol and paraffin-embedded according to standard protocol. 5 μm slides were cut and placed on glass slides for immunohistochemistry analysis for TP53 protein and H&E staining. Organoid injection, collection, and sectioning were performed blinded.

#### Tumor Cell Isolation and Characterization

Tumor tissues were removed from the animals and washed with 1X PBS. Tumors were minced with razor blades on an aseptic surface, transferred into a 15 mL canonical tube, and washed with PBS. Then cells were dissociated by incubating in nFTM media with 300 units/ml collagenase and 100 units/ml hyaluronidase (STEMCELL, #07912) and 2 units/ml DNaseI at 4°C overnight and centrifuged at 100 g for 10 minutes at 4°C to remove undigested tissue. Then, the supernatant was collected and centrifuged at 500 g for 10 minutes at 4°C and washed with PBS twice. Cells were resuspended in nFTM and passed through a 40 μm nylon mesh filter and then centrifuged at 500 g for 5 minutes at 4°C. Single cells were cultured on growth factor-reduced Matrigel Matrix (BD Biosciences)-coated plates at 37°C in a 5% CO_2_ incubator.

Single cells from tumors were plated on L-glass coverslips and grown as above. Cells were fixed in 4% PFA at room temperature for 10–15 minutes on day 5. Fixed coverslips were washed in PBS and permeabilized in PBS containing 2% Triton X-100 for 10 minutes at room temperature, then blocked in a solution of 10% Normal Donkey Serum in PBS-T for 1 hour at room temperature, followed by 2 hours incubation at room temperature in primary antibodies in blocking solution. The following primary antibodies were used at 1:200 dilution: PAX8 (Proteintech, 21384–1-AP), CDH1 (R&D System, AF648), Tp53 (DO-1) (Santa Cruz Biotechnology, sc-126), Ki-67 (SP6) (Invitrogen, MA5–14520), Anti-phospho-Histone H2A.X (Ser139) (Millipore, clone JBW301), Human Nuclei (MAB128–1). The coverslips were then washed with PBS-T (3X) for 15 minutes each at room temperature and incubated with species-specific AF488 or AF594-conjugated secondary antibodies (Invitrogen) at 1:400 dilution, followed by DAPI (Molecular Probes, D3571) counterstain. Coverslips were mounted on a glass slide, and images were acquired using a Nikon/Leica microscope.

#### PARPi Treatments

Cells from organoids or primary tumor cells were plated in flat-bottom 96-well plates at 1×10^4^ per well and incubated for 3 days in FTE media. Rucaparib (AG-014699, Niraparib (Synonyms: MK-4827), and Olaparib (Synonyms: AZD2281; KU0059436) were prepared in 100 mM stock in DMSO and diluted according to treatments in the media. 100 μL of FTE media containing PARPi was add to the cells, and media was collected after 24, 48, and 72 hours for a LDH assay (CyQUANT, C20301), and cells were treated with the PrestoBlue (Invitrogen, 226392) reagent for cell viability assay.

#### LDH Assay

25 μL of media from PARPi treated cells were mixed with 25 μL of LDH substrate in a 96-well clear plate, and plates were incubated at room temperature in the dark for 30 minutes. 25 μL of LDH blocking solution were added to stop the reaction, and absorbance was measured at 490nm and 680nm. To determine LDH activity, the 680nm absorbances were subtracted from 490nm absorbance.

#### PrestoBlue Assays

After the PARPi treatments, 10 μL of PrestoBlue reagent was added into the well and incubated 1 hour at 37°C. Change in the fluorescent signal was recorded at 560/ 590 nm excitation/emission wavelengths.

### QUANTIFICATION AND STATISTICAL ANALYSIS

Statistical analyses were performed by using Prism software (GraphPad Software, La Jolla, California). All quantitative data were expressed as mean values ± Standard Error of the Mean (SEM) and analyzed by analysis of variance (ANOVA) with unequal variances known as Welch ANOVA or two-tailed paired t test followed by analysis of mean differences in three biological replicates. Differences were considered significant at *p ≤ 0.05, ** p ≤ 0.01, and *** p ≤ 0.001 and **** p ≤ 0.0001.

### ADDITIONAL RESOURCES

Our study has not generated or contributed to a new website/forum, and it is not part of a clinical trial.

## Supplementary Material

Published open access Supplemental Materials

## Figures and Tables

**Table 1.** Clinical summary of controls and ovarian *BRCA1*^*mut*^ cancer patients

Cell ID	Parent tissue	Biopsy specimen	Sex	Karyotype	Mutation	Cancer stage	Age (years)
CS87i-CTR-n3	Lymphoblastoid cells	Blood	Female	Normal	None		Unknown
CS01iMEC-CTR-n4	Mammary epithelial cells	Mammary Epithelial	Female	Normal	None		Unknown
CS80i-CTR-Tn3	PBMCs	Blood	Female	Normal	None		48
CS08i-BRCA-n5	Lymphoblastoid cells	Blood	Female	Normal	BRCA1: 3875del4	IIC	36.3
CS70i-BRCA-n1	Lymphoblastoid cells	Blood	Female	Normal	BRCA1: 1048delA	IIIC	33.6
CS79i-BRCA-n2	Lymphoblastoid cells	Blood	Female	Normal	BRCA1: 1IVS5+1G > A	IIIC	31.4

**Table:** KEY RESOURCES TABLE

REAGENT or RESOURCE	SOURCE	IDENTIFIER
Antibodies		
PAX8	Proteintech	Cat#21384–1-AP
TUBB4A	Abcam	Cat# ab11315; RRID:AB_297919
POU5F1	Stemgent	Cat#09–0023; RRID:AB_2167689
Nanog	Stemgent	Cat#09–0020; RRID:AB_2298294
SOX2	Stemgent,	Cat#09–0024: RRID:AB_2195775
TRA-1−60	Stemgent	Cat#09–0010; RRID:AB_1512170
TRA-1−81	Stemgent	Cat#09–0011; RRID:AB_1512171
SSEA4	Stemgent	Cat#09–0006; RRID:AB_1512169
CDH1	R&D System	Cat#AF648; RRID:AB_355504
OVGP1	SIGMA	Cat#HPA062205; RRID:AB_2684710
p53 (DO-1)	Santa Cruz Biotechnology	Cat#sc-126; RRID:AB_628082
Ki-67 (SP6)	Invitrogen	Cat#MA5–14520; RRID:AB_10979488
MUC-16	Sigma	Cat#HPA065600; RRID:AB_2732736
Anti-phospho-Histone H2A.X (Ser139)	Millipore	Cat#16–202A; RRID:AB_568825
Human Nuclei	Millipore	Cat#MAB128–1; RRID:AB_94090
BRCA1	Millipore	Cat#OP92; RRID:AB_2750876
AF488	Invitrogen	Cat#A32790; RRID:AB_2762833 and Cat# A32766; RRID:AB_2762823
AF594	Invitrogen	Cat#A32754; RRID:AB_2762827 and Cat# A32744; RRID:AB_2762826
Chemicals, peptides, and recombinant proteins		
Human recombinant activin A	Stemgent	Cat#03–0001
Human recombinant BMP4	R&D Systems	Cat#314-BP
Human Noggin	R&D system	Cat#6057-NG-01M
Murine recombinant EGF	PeproTech	Cat#AF-100–15
Human recombinant basic FGF	PeproTech	Cat#100–18B
Human recombinant Follistatin	PeproTech	Cat#120–13
Human recombinant WNT4	R&D Systems	Cat#6076-WN-005
SB431542	Tocris	Cat#1614
Glutamax	Life Technology	Cat#35050–061
HEPES	Life Technology	Cat#15–630-106
B27 supplement	GIBCO	Cat#17504044
N2 supplement	GIBCO	Cat#17502048
Matrigel	Corning/BD Biosciences	Cat#354230
penicillin/ streptomycin	GIBCO	Cat#15140122
Y-27632	Stemgent	Cat#04–0012-02
SYBR Select Master Mix	Appliedbiosystem	Cat#4472908
OCT	Tissue-Tek	Cat#MPSMK-981385
DAPI	Molecular Probes	Cat#D3571
DAB solution	Vector	Cat#SK-4105
Avidin-Biotin Complex	Vector	Cat#PK-4000
Formalin	Fisher Chemical	Cat#23–245685
ImmunoMaster Hematoxylin	American MasterTech Scientific	Cat#HXIMHPT
EOSIN WITH PHLOXINE B	American MasterTech Scientific	Cat#MPSM-STLB117
Hyaluronidase	STEMCELL	Cat#07912
Rucaparib (AG-G14699	Chemietek	Cat#CT-AG01−10mg
OLAPARIB (AZD2281)	Chemietek	Cat#CT-A2281–100mg
NIRAPARIB (MK-4827)	Chemietek	Cat#CT-MK4827–10mg
Antibiotic-Antimycotic	GIBCO	Cat#15240-062
hLIF	Millipore	Cat#LIF1010
CHIR99021	Tocris	Cat#4423
HA-100	Santa Cruz Biotech	Cat#203072
A-83-10	Tocris	Cat#2939
mTeSR®1 medium	STEMCELL	Cat#85850
KOSR	Invitrogen	Cat#10828028
Advanced DMEM/F-12	Life technologies	Cat#12634028
Phosphatase/protease inhibitor cocktail	Sigma Aldrich	Cat#MSSAFE
Critical commercial assays		
B cell Nucleofector Kit	Lonza	Cat#VPA-1001
QIAGEN RNeasy Mini kit	QIAGEN	Cat#74004
Quantitect Reverse Transcription Kit	QIAGEN	Cat#205311
LDH assay	CyQUANT	Cat#C20301
PrestoBlue assay	Invitrogen	Cat#226392
4X Laemmli sample buffer	BIO-RAD	Cat#161-0774
Bradford assay	BIO-RAD	Cat#5000006
Mini-PROTEAN TGX Precast gels	BIO-RAD	Cat#456–1094
Odyssey blocking buffer	LI-COR	Cat#927–40000
Deposited data		
RNA Seq Data	This Paper	GEO: GSE190134
Experimental models: Cell lines		
CS87i-CTR-n3, female, age unknown, BRCA1 wildtype	Cedars-Sinai iPSC Core	CS87i-CTR
CS01iMEC-CTR, female, age unknown, BRCA1 wildtype	Cedars-Sinai iPSC Core	CS01iMEC-CTR
CS80i-CTR-Tn3, female, age 48, BRCA1 wildtype	Cedars-Sinai iPSC Core	CS80i-CTR
CS08i-BRCA-n5, female, age 36.3, BRCA1 mutant	Cedars-Sinai iPSC Core	CS08i-BRCA
CS70i-BRCA-n1, female, age 33.6, BRCA1 mutant	Cedars-Sinai iPSC Core	CS70i-BRCA
CS79i-BRCA-n2, female, age 31.4, BRCA1 mutant	Cedars-Sinai iPSC Core	CS79i-BRCA
Experimental models: Organisms/strains		
Mouse: *Foxn1*^*nu*^,formerly *Hfh11*^*nu*^ *(known as* NU/J athymic nude)	Jackson Laboratory	Cat#002019
Oligonucleotides		
Primers for Real-time PCR, see Table S5		N/A
Recombinant DNA		
pEP4 E02S ET2K	Toombs et al., 2020	Addgene 20927
pCXLE-hOCT3/4-shp53-F	Toombs et al., 2020	Addgene 27077
pCXLEhUL	Toombs et al., 2020	Addgene 27080
pCXLE-hSK	Toombs et al., 2020	Addgene 27078
pCXWB-EBNA1	Toombs et al., 2020	Addgene 37624
Software and algorithms		
ImageJ	Schneider et al., 2012	https://imagej.nih.gov/ij/
Neurolucdia 11.07	MBF Bioscience	https://www.mbfbioscience.com/
Stereo Investigator 11.07	MBF Bioscience	https://www.mbfbioscience.com/stereo-investigator
Prism software	GraphPad Software	https://www.graphpad.com/scientific-software/prism/

## Highlights

- *BRCA1*^*mut*^ patient iPSC lines can be differentiated into fallopian tube epithelium
- *BRCA1*^*mut*^ fallopian tubes recapitulate ovarian carcinogenesis *in vitro* and *in vivo*
- *BRCA1*^*mut*^ fallopian tubes may provide a model to predict disease severity
- *BRCA1*^*mut*^ fallopian tube organoids provide a platform to study efficacy of cancer drugs
