# Supplementary material for: Human iPSC-derived fallopian tube organoids with BRCA1 mutation recapitulate early-stage carcinogenesis
Source: Cell Rep. Author manuscript; Available in PMC 2022 Apr 11. (PMC9000920; doi:10.1016/j.celrep.2021.110146)
Supplement: Published open access Supplemental Materials [file NIHMS1792247-supplement-Published_open_access_Supplemental_Materials.pdf]

Cell Reports, Volume *37*

Supplemental information

Human iPSC-derived fallopian tube organoids with *BRCA1* mutation recapitulate early-stage carcinogenesis

Nur Yucer, Rodney Ahdoot, Michael J. Workman, Alexander H. Laperle, Maria S. Recouvreux, Kathleen Kurowski, Diana J. Naboulsi, Victoria Liang, Ying Qu, Jasmine T. Plummer, Simon A. Gayther, Sandra Orsulic, Beth Y. Karlan, and Clive N. Svendsen

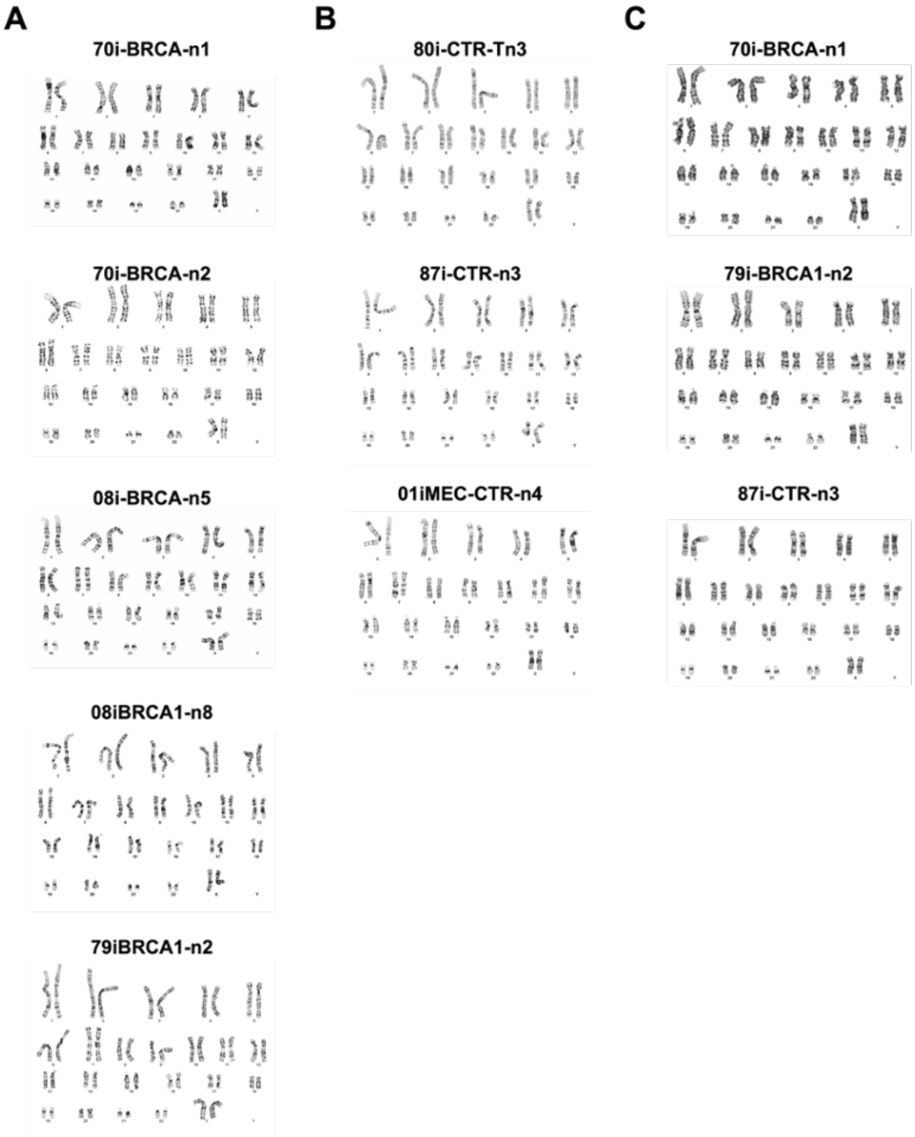

Figure S1 (Related to Figure1). ***BRCA1****^{mut}***-iPSC lines exhibit normal karyotypes, even after extensive culturing.** G-band analysis of karyotype for (A) *BRCA1*^{mut}-iPSC lines and (B) control iPSC lines at passage 9. (C) Karyotype analysis for 87i-CTR-n3 control and *BRCA1* mutant 70i-BRCA-n1 and 79i-BRCA-n2 at passage 32.

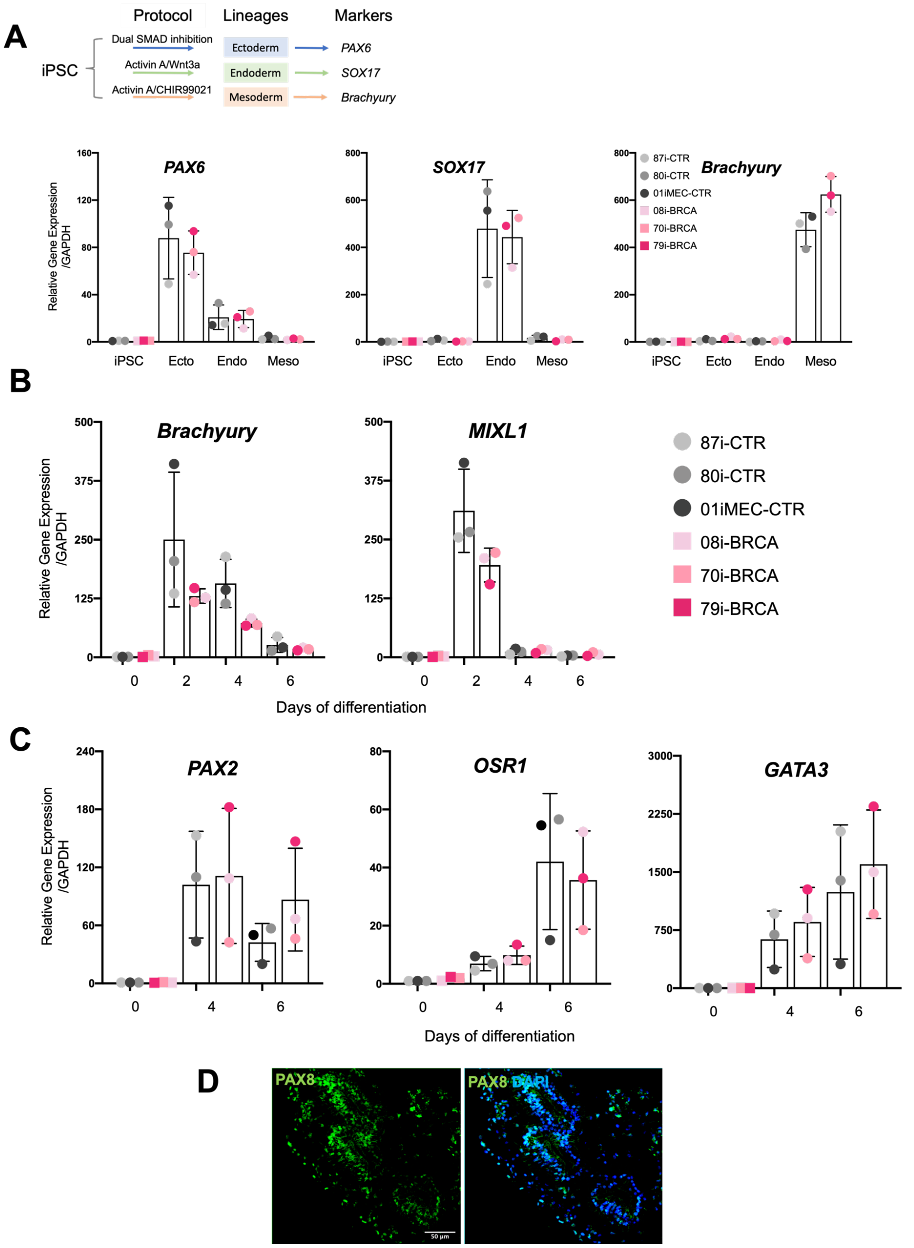

Figure S2 (Related to Figure1). *BRCA1^{mut}*-iPSC lines have the capacity to differentiate into three germ layers and (Related to Figure2) to differentiate of *BRCA1^{mut}*-iPSC into Intermediate Mesoderm-Like Cells. (A) The factors and markers involved in the differentiation of three different germ layers (Chambers et al., 2009; Mae et al., 2013; Teo et al., 2014; Yucer et al., 2017). Expression of ectoderm marker *PAX6*, mesoderm marker *BRACHYURY*, and endoderm marker *SOX17* during course of differentiations. Relative gene expression to iPSC stage (day 0) was calculated using the ΔΔCt method and normalized to endogenous *GAPDH* level. Error bars are Standard Error of the Mean (SEM) (n = 3 independent biological experiments and each dot represents the average of 3 independent

biological experiments per line). (B) Expression kinetics of mesoderm markers *BRACHYURY* and *MIXL1* during the 6-day differentiation course using established protocol (Yucer et al., 2017). (C) Expression of intermediate mesoderm markers, *PAX2, OSR1,* and *GATA3* during the 6-day differentiation course. (D) Immunocytochemistry for FTE marker PAX8 in *BRCA1^{mut}*-FTE organoid (70i-BRCA line) at day 30 in culture. Scale bars, 50 µm. Relative gene expression at iPSC stage (day 0) was calculated using the ΔΔCt method and normalized to endogenous *GAPDH* level. Error bars are Standard Error of the Mean (SEM) (n = 3 independent biological experiments and each dot represents the average of 3 independent biological experiments per line).

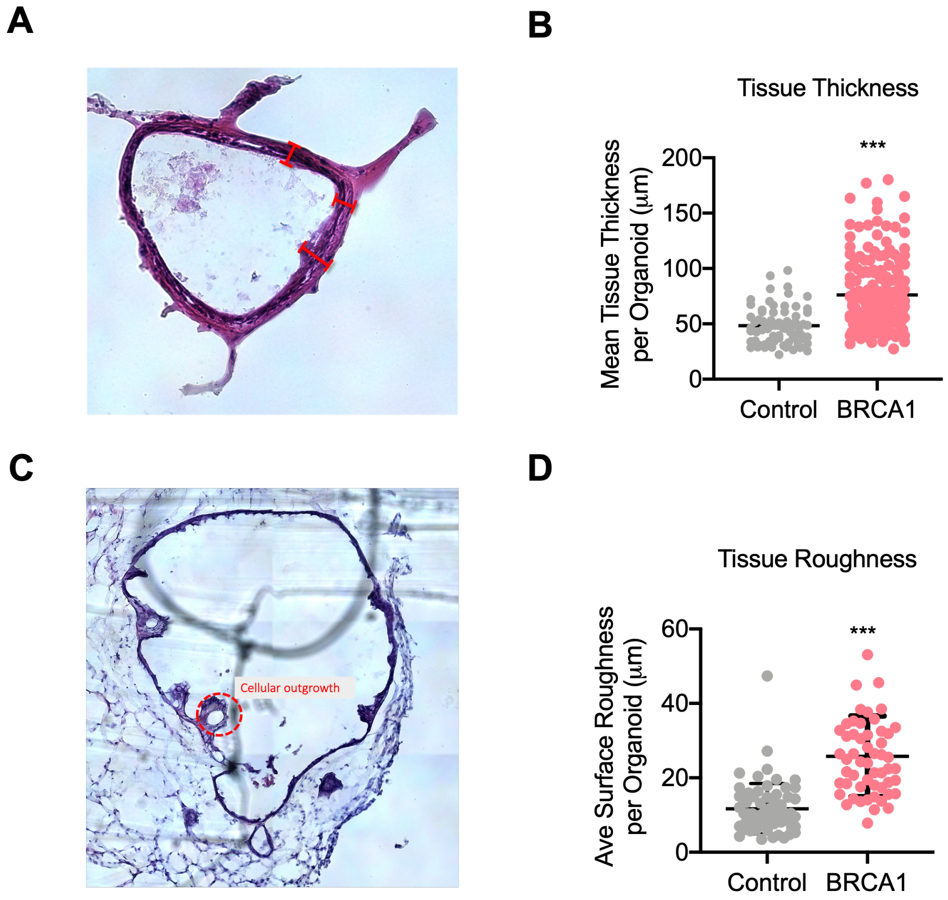

The arithmetic average roughness (cellular outgrowths), R_a,

$$R_a = \frac{1}{n}\sum_{i=1}^{n}|y_i|$$

Figure S3 (Related to Figure3). Histological characterization of controls and *BRCA1^{mut}* FTE organoids. (A) H&E staining of normal FTE organoids at 4-months and quantification tissue thickness distribution. (B) FTE organoids thickness distribution by genotype. (C) H&E staining image, which was stitched using Stereo

Investigator to indicate the cellular outgrowth (D) Cellular outgrowth measured as arithmetic average roughness by genotype.

$$R_a = \frac{1}{n}\sum_{i=1}^{n}|y_i|$$

Significance calculated using student t-test, ***p<0.00

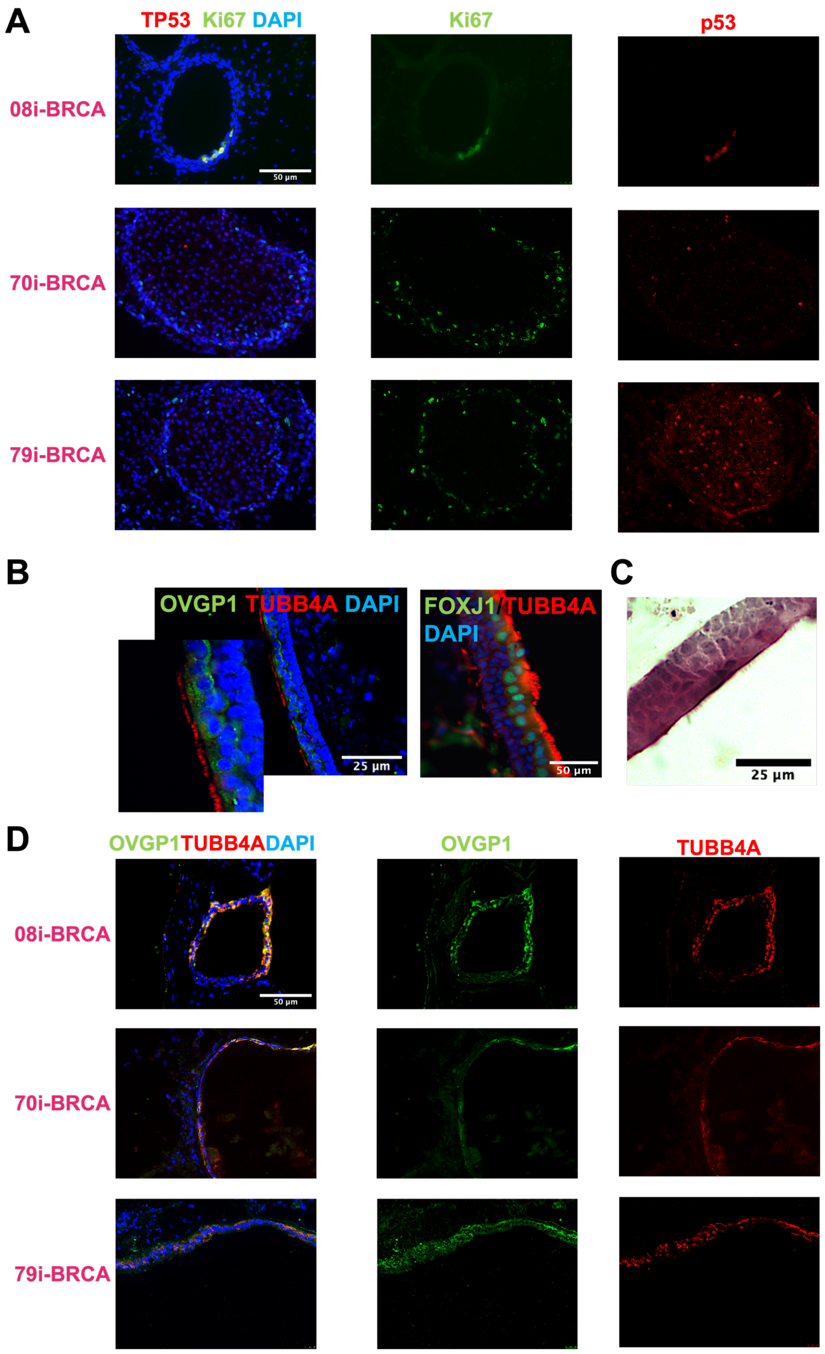

Figure S4 (Related to Figure4). ***BRCA1*^{mut}-iPSC-derived fallopian tube organoids present cellular signature of STIC pathology *in vitro*.** (A) Immunocytochemistry for TP53 and Ki67 for 3 different *BRCA1*^{mut}-FTE-organoids. Scale bars, 50 µm. Cilia formation at 6-months shown by (B) immunocytochemistry for FTE secretory cell marker OVGP1 and ciliated cell marker TUBB4A and FOXJ1 and (C) H&E staining in 80iCTR line organoids. Scale bars, 25 µm and 50 µm. (D) Immunocytochemistry for FTE secretory cell marker OVGP1 and ciliated cell marker TUBB4A in for 3 different *BRCA1*^{mut}-FTE organoids at 4 months. Scale bars, 50 µm.

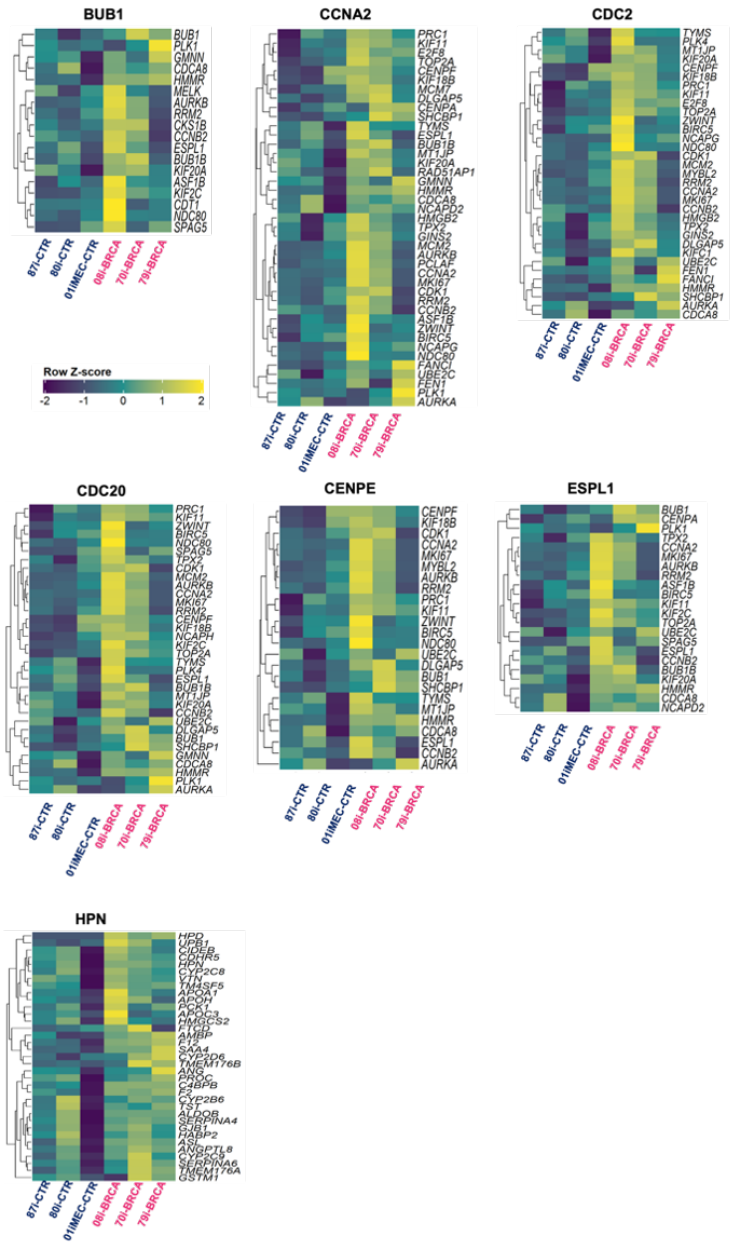

Figure S5 (Related to Figure4). ***BRCA1**^{mut}***-iPSC-derived fallopian tube organoids exhibit the molecular basis for STIC pathology *in vitro*.** Differential gene expression and GSEA for control and *BRCA1*^{mut}-FTE organoids at 4-month in culture. FDR value is < 0.05. Z-scores of the leading-edge genes were calculated and used to construct a heatmap.

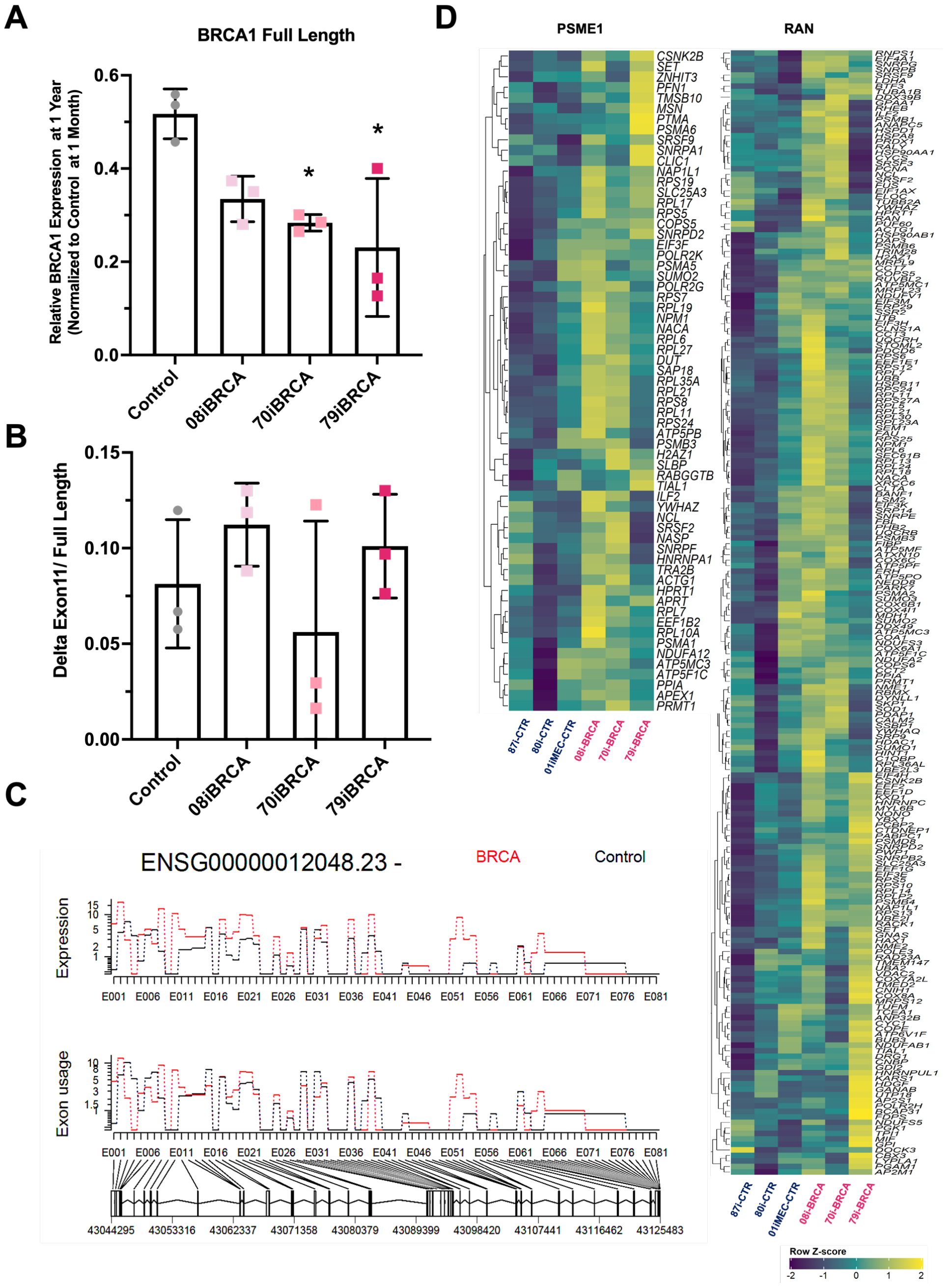

Figure S6 (Related to Figure 5). The decrease in *BRCA1* expression in *BRCA1*^{mut}-iPSC-derived fallopian tube organoid is not due to loss of heterozygosity (LOH). (A) qRT-PCR quantification of full-length *BRCA1* gene expression at 1 month and 1 year. (B) qRT-PCR quantification of Delta exon 11 *BRCA1* gene expression. (C) Fragment mapping for the *BRCA1* gene on RNA sequencing data at 1-year culture. Relative gene expression to 87i-CTR was calculated using ΔΔCt method and normalized to endogenous *GAPDH* level. Error bars are Standard Error of the Mean (SEM) (n = 3 independent biological experiments and each dot represents the average of 3 independent biological experiments per line). Significance calculated using One-way ANOVA, Dunnett Multiple comparisons,*p<0.05. (D) Differential gene expression and GSEA for control and *BRCA1*^{mut}-FTE organoids at 1 year in culture. FDR value is < 0.05. Z-scores of the leading-edge genes were calculated and used to construct a heatmap.

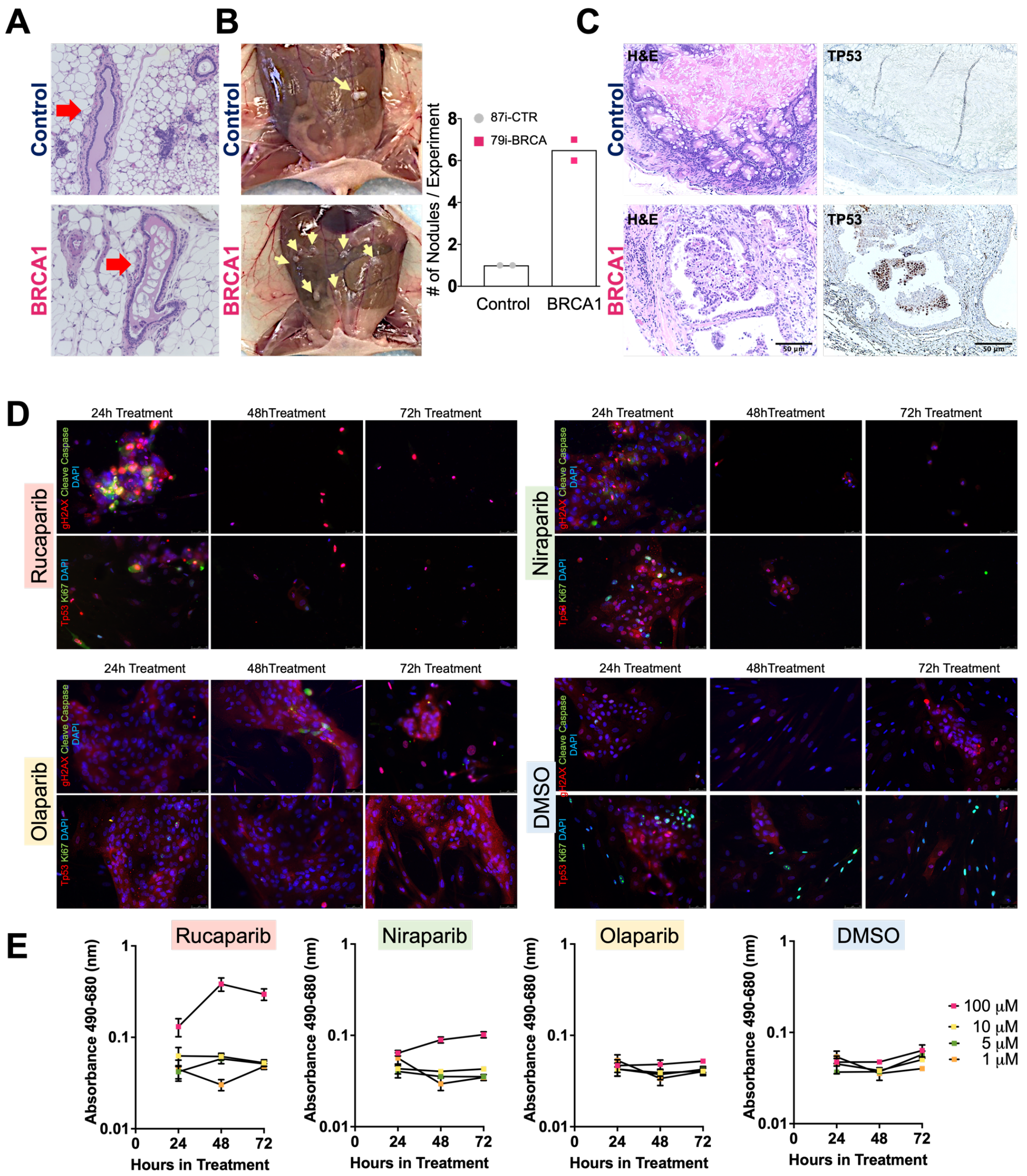

Figure S7 (Related to Figure6). ***BRCA1^{mut}*-iPSC-derived fallopian tube organoids demonstrate cancerous characteristics *in vivo*.** (A) H&E images for normal control and *BRCA1^{mut}*-FTE organoids within the mammary gland. (B) Brightfield images at 3 months post-transplantation of 87i-CTR FTE organoids and 79i-BRCA FTE organoids (after 4 months in culture), and total number of nodules per experiment. (C) H&E and TP53 immunohistochemistry images at 3 months post-transplantation. Scale bars, 50 µm.(D) Immunocytochemistry for TP53, Ki67, gH2AX and Cleaved Caspase3 in response to 100 mM DMSO, Rucaparib, Niraparib and Olaparib at 24, 48 and 72 hours treatment of primary cells from *BRCA1^{mut}* tumor lesions. (E) Time course of cellular

cytotoxicity assay (LDH assay) in response to 100, 10, 5 and 1 mM DMSO, Rucaparib, Niraparib and Olaparib at 24, 48 and 72 hours treatment of primary cells from *BRCA1^mut^* tumor lesions.

Table S1 (Related to Figure3). Gene enrichment sets for control vs *BRCA1^mut^*-FTE organoids at 4-month culture

NAME	SIZE	ES	NES	NOM p-val	FDR q-val	FWER p-val	RANK AT MAX	LEADING EDGE
CAR_HPX	58	0.6686738	2.296964	0	0	0	1512	tags=36%, list=8%, signal=39%
GNF2_CCNA2	67	0.65380585	2.2726195	0	0	0	3785	tags=61%, list=19%, signal=76%
GNF2_CCNB2	56	0.6678471	2.2662804	0	0	0	3653	tags=61%, list=19%, signal=75%
GNF2_CDC20	55	0.66336524	2.2533634	0	0	0	3785	tags=62%, list=19%, signal=77%
GNF2_HMMR	47	0.6797286	2.2286162	0	0	0	3785	tags=68%, list=19%, signal=84%
GNF2_CDC2	61	0.6328634	2.2126033	0	0	0	3653	tags=54%, list=19%, signal=66%
GNF2_CENPF	61	0.636856	2.1734078	0	1.60E-04	0.001	3653	tags=56%, list=19%, signal=68%
GNF2_CENPE	40	0.6784023	2.1681633	0	1.40E-04	0.001	3653	tags=60%, list=19%, signal=74%
GNF2_HPX	111	0.562162	2.148408	0	1.25E-04	0.001	1688	tags=32%, list=9%, signal=34%
MODULE_24	404	0.48340335	2.1414783	0	1.12E-04	0.001	2470	tags=31%, list=13%, signal=35%
GNF2_HPN	111	0.5525837	2.114176	0	2.00E-04	0.002	1688	tags=32%, list=9%, signal=34%
GNF2_TST	92	0.5701473	2.1135342	0	1.84E-04	0.002	1688	tags=33%, list=9%, signal=36%
MODULE_342	202	0.5081765	2.1120644	0	2.52E-04	0.003	1930	tags=34%, list=10%, signal=38%
GNF2_MKI67	28	0.71962315	2.095437	0	3.16E-04	0.004	3652	tags=71%, list=19%, signal=88%
GNF2_PCNA	67	0.5948131	2.0905054	0	3.72E-04	0.005	4503	tags=60%, list=23%, signal=77%
GNF2_LCAT	105	0.5475117	2.084383	0	3.49E-04	0.005	2034	tags=33%, list=10%, signal=37%
GNF2_BUB1	26	0.70440525	2.075836	0	3.95E-04	0.006	4113	tags=65%, list=21%, signal=83%
GNF2_RRM2	39	0.6553588	2.0745828	0	3.73E-04	0.006	3785	tags=64%, list=19%, signal=79%
GNF2_ESPL1	35	0.6514922	2.0113218	0	0.001224449	0.02	3785	tags=63%, list=19%, signal=78%
GNF2_GSTM1	93	0.5400912	2.004573	0	0.001499126	0.026	1688	tags=30%, list=9%, signal=33%
MODULE_180	115	0.5121064	1.989555	0	0.001798731	0.033	2229	tags=39%, list=11%, signal=44%
GNF2_MCM4	53	0.58221394	1.9832239	0.001941748	0.001918978	0.037	3886	tags=55%, list=20%, signal=68%

Table S2 (Related to Figure3). Functional annotation for control vs *BRCA1^{mut}* -FTE organoids at 4 months by IPA software

	Categories	Diseases or Functions Annotation	p-value	z-score	Gene List	# Molecules
1	Cancer,Organismal Injury and Abnormalities	Extracranial solid tumor	2.5E-48	1.023	A1CF,ABCB11,ABCC3	1591
2	Cancer,Organismal Injury and Abnormalities	Solid tumor	7.95E-48	0.139	A1CF,ABCB11,ABCC3	1598
3	Cancer,Organismal Injury and Abnormalities	Cancer	1.5E-47	0.821	A1CF,ABCB11,ABCC3	1597
4	Cancer,Organismal Injury and Abnormalities	Malignant solid tumor	1.92E-47	0.327	A1CF,ABCB11,ABCC3	1590
5	Cancer,Organismal Injury and Abnormalities	Non-hematological solid tumor	5.85E-44	0.351	A1CF,ABCB11,ABCC3	1562
6	Cancer,Organismal Injury and Abnormalities	Nonhematologic malignant neoplasm	1.15E-43	0.587	A1CF,ABCB11,ABCC3	1557
7	Cancer,Organismal Injury and Abnormalities	Tumorigenesis of tissue	1.52E-42	0.698	A1CF,ABCB11,ABCC3	1544
8	Cancer,Organismal Injury and Abnormalities	Abdominal neoplasm	2.39E-41	1.665	A1CF,ABCB11,ABCC3	1481
9	Cancer,Organismal Injury and Abnormalities	Non-melanoma solid tumor	1.53E-40	-0.481	A1CF,ABCB11,ABCC3	1544
10	Cancer,Organismal Injury and Abnormalities	Carcinoma	6.19E-40	0.771	A1CF,ABCB11,ABCC3	1531
11	Cancer,Organismal Injury and Abnormalities	Epithelial neoplasm	7.53E-40	0.511	A1CF,ABCB11,ABCC3	1533
12	Cancer,Organismal Injury and Abnormalities	Abdominal cancer	2.81E-39	2.458	A1CF,ABCB11,ABCC3	1460
13	Cancer,Organismal Injury and Abnormalities	Adenocarcinoma	3.78E-34	1.986	A1CF,ABCB11,ABCC3	1363
14	Cancer,Organismal Injury and Abnormalities	Abdominal carcinoma	1.36E-32	2.431	A1CF,ABCB11,ABCC3	1402
15	Cancer,Gastrointestinal Disease,Organismal Injury and Abnormalities	Digestive organ tumor	2.73E-32	1.602	A1CF,ABCB11,ABCC3	1394
16	Cancer,Organismal Injury and Abnormalities	Abdominal adenocarcinoma	2.97E-31	1.673	A1CF,ABCB11,ABCC3	1329
17	Cancer,Gastrointestinal Disease,Organismal Injury and Abnormalities	Digestive system cancer	1.61E-28	2.298	A1CF,ABCB11,ABCC3	1359
18	Cancer,Organismal Injury and Abnormalities	Genitourinary tumor	5.49E-28	0.512	A1CF,ABCC3,ABCF2,A	1078
19	Cancer,Gastrointestinal Disease,Organismal Injury and Abnormalities	Gastrointestinal tumor	8.6E-28	0.255	ABCB11,ABCC3,ABCF	1266
20	Cancer,Organismal Injury and Abnormalities	Malignant genitourinary solid tumor	3.74E-27	0.304	A1CF,ABCC3,ABCF2,A	1058
21	Cancer,Gastrointestinal Disease,Organismal Injury and Abnormalities	Gastrointestinal tract cancer	1.21E-25	1.388	ABCB11,ABCC3,ABCF	1253
22	Cancer,Gastrointestinal Disease,Organismal Injury and Abnormalities	Intestinal tumor	2.97E-25	0.722	ABCB11,ABCC3,ABCF	1211
23	Cancer,Gastrointestinal Disease,Organismal Injury and Abnormalities	Large intestine neoplasm	5.19E-25	1.497	ABCB11,ABCC3,ABCF	1209
24	Cancer,Organismal Injury and Abnormalities	Head and neck tumor	7.29E-25	-0.343	A1CF,ABCB11,ABCC3	1248
25	Cancer,Organismal Injury and Abnormalities	Incidence of tumor	3.8E-24	0.537	A1CF,ABCB11,ABCC3	1084
26	Cancer,Gastrointestinal Disease,Organismal Injury and Abnormalities	Intestinal cancer	1.9E-23	1.585	ABCB11,ABCC3,ABCF	1199
27	Cancer,Gastrointestinal Disease,Organismal Injury and Abnormalities	Malignant neoplasm of large intestine	2.38E-23	1.913	ABCB11,ABCC3,ABCF	1198
28	Cancer,Organismal Injury and Abnormalities	Anogenital cancer	3.48E-23	-0.248	A1CF,ABCC3,ABCF2,A	978
29	Cancer,Organismal Injury and Abnormalities	Head and neck cancer	4.48E-23	0.026	A1CF,ABCB11,ABCC3	1229
30	Cancer,Organismal Injury and Abnormalities	Head and neck carcinoma	2.34E-22	0	A1CF,ABCB11,ABCC3	1220
31	Cancer,Organismal Injury and Abnormalities	Cancer of secretory structure	2.81E-22	0.28	A1CF,ABCB11,ABCC3	1278
32	Cancer,Endocrine System Disorders,Organismal Injury and Abnormalities	Endocrine gland tumor	6.67E-22	0.072	A1CF,ABCB11,ABCC3	1183
33	Cancer,Organismal Injury and Abnormalities	Pelvic tumor	7.89E-22	-0.242	A1CF,ABCC3,ABCF2,A	947
34	Cancer,Organismal Injury and Abnormalities,Reproductive System Disease	Breast or gynecological cancer	9.69E-22	-0.363	A1CF,ABCC3,ABCF2,A	790
35	Cancer,Organismal Injury and Abnormalities	Pelvic cancer	1.37E-21	0.114	A1CF,ABCC3,ABCF2,A	933
36	Cancer,Organismal Injury and Abnormalities	Frequency of tumor	1.85E-21	1.137	A1CF,ABCB11,ABCC3	1045
37	Cancer,Organismal Injury and Abnormalities	Tumorigenesis of epithelial neoplasm	2.36E-21	1.112	A1CF,ABCB11,ABCC3	1031
38	Cancer,Endocrine System Disorders,Organismal Injury and Abnormalities	Nonpituitary endocrine tumor	2.78E-21	0.179	A1CF,ABCB11,ABCC3	1175
39	Cancer,Organismal Injury and Abnormalities	Development of carcinoma	4.96E-21	1.388	A1CF,ABCB11,ABCC3	1018
40	Cancer,Organismal Injury and Abnormalities	Genitourinary carcinoma	6.26E-21	1.48	A1CF,ABCC3,ABCF2,A	922
41	Cancer,Organismal Injury and Abnormalities	Development of malignant tumor	7.19E-21	1.189	A1CF,ABCB11,ABCC3	1028
42	Cancer,Organismal Injury and Abnormalities,Reproductive System Disease	Tumorigenesis of reproductive tract	8.6E-21	0.239	A1CF,ABCC3,ABCF2,A	664
43	Cancer,Organismal Injury and Abnormalities,Reproductive System Disease	Female genital neoplasm	8.89E-21	0.239	A1CF,ABCC3,ABCF2,A	665
44	Cancer,Organismal Injury and Abnormalities,Reproductive System Disease	Female genital tract cancer	1.33E-20		A1CF,ABCC3,ABCF2,A	646
45	Cancer,Gastrointestinal Disease,Organismal Injury and Abnormalities	Gastrointestinal carcinoma	1.87E-20	0.426	ABCB11,ABCC3,ABCF	1187
46	Cancer,Organismal Injury and Abnormalities	Neck neoplasm	2.01E-20		A1CF,ABCB11,ABCC3	1172
47	Cancer,Endocrine System Disorders,Organismal Injury and Abnormalities	Thyroid gland tumor	8.8E-20		A1CF,ABCB11,ABCC3	1159
48	Inflammatory Response,Organismal Injury and Abnormalities	Inflammation of organ	1.06E-19	-1.399	ABCB11,ACE2,ACKR4	275
49	Cancer,Organismal Injury and Abnormalities,Reproductive System Disease	Genital tumor	1.83E-19	-0.224	A1CF,ABCC3,ABCF2,A	886
50	Organismal Development	Morphology of body cavity	2.8E-19		ABCB11,ACE2,ACSL1	319
51	Cancer,Gastrointestinal Disease,Organismal Injury and Abnormalities	Gastrointestinal adenocarcinoma	3.69E-19	0.555	ABCB11,ABCC3,ABCF	1147
52	Cancer,Organismal Injury and Abnormalities,Reproductive System Disease	Genital tract cancer	3.92E-19	-0.655	A1CF,ABCC3,ABCF2,A	871
53	Cancer,Endocrine System Disorders,Organismal Injury and Abnormalities	Endocrine cancer	7.15E-19		A1CF,ABCB11,ABCC3	1156
54	Cancer,Endocrine System Disorders,Organismal Injury and Abnormalities	Endocrine carcinoma	7.82E-19		A1CF,ABCB11,ABCC3	1154
55	Cancer,Endocrine System Disorders,Organismal Injury and Abnormalities	Thyroid cancer	1.31E-18		A1CF,ABCB11,ABCC3	1151
56	Cancer,Endocrine System Disorders,Organismal Injury and Abnormalities	Thyroid carcinoma	1.69E-18		A1CF,ABCB11,ABCC3	1149
57	Cancer,Organismal Injury and Abnormalities	Genitourinary adenocarcinoma	5.73E-18	1.709	A1CF,ABCC3,ABCF2,A	824
58	Organismal Development,Organismal Injury and Abnormalities	Abnormal morphology of abdomen	8.38E-18		ABCB11,ACE2,ACTG2	199
59	Cancer,Gastrointestinal Disease,Organismal Injury and Abnormalities	Intestinal carcinoma	1.87E-17	0.816	ABCB11,ABCC3,ABCF	1122
60	Cancer,Gastrointestinal Disease,Organismal Injury and Abnormalities	Large intestine carcinoma	2.48E-17		ABCB11,ABCC3,ABCF	1121
61	Cancer,Organismal Injury and Abnormalities	Breast or pancreatic cancer	2.9E-17	0.265	ABCB11,ABCC3,ABCF	695
62	Cancer,Organismal Injury and Abnormalities,Reproductive System Disease	Development of genital tumor	3.55E-17	0.13	A1CF,ABCC3,ABCF2,A	627
63	Cancer,Organismal Injury and Abnormalities	Neoplasia of cells	9.27E-17	0.896	A1CF,ABCB11,ABCC3	768
64	Cancer,Organismal Injury and Abnormalities,Reproductive System Disease	Female genital tract serous carcinoma	1.08E-16		ACTG2,ALDH1A1,ANX	99
65	Dermatological Diseases and Conditions,Organismal Injury and Abnormalities	Psoriasis	1.85E-16	2.449	AGER,ALDH1A3,ANXA	127
66	Cancer,Gastrointestinal Disease,Organismal Injury and Abnormalities	Intestinal adenocarcinoma	1.95E-16	0	ABCB11,ABCC3,ABCF	1109
67	Inflammatory Disease	Chronic inflammatory disorder	2.59E-16	1.347	ACSL1,ACTA1,ADCY8	225
68	Cancer,Gastrointestinal Disease,Organismal Injury and Abnormalities	Large intestine adenocarcinoma	2.72E-16		ABCB11,ABCC3,ABCF	1108
69	Cancer,Organismal Injury and Abnormalities,Reproductive System Disease	Breast or ovarian cancer	5.27E-16	-0.063	ABCC3,ABCF2,ABLIM	527
70	Cancer,Organismal Injury and Abnormalities	Breast or colorectal cancer	5.5E-16	1.148	ABCC3,ABCF2,ABLIM	840
71	Digestive System Development and Function	Morphology of digestive system	1.02E-15		ABCB11,AGR2,AGTR2	155
72	Organismal Development	Abnormal morphology of body cavity	1.13E-15		ABCB11,ACE2,ACSL1	277
73	Cellular Movement	Cell movement	3.22E-15	-0.801	ACE2,ACKR4,ADAMTS	407
74	Cancer,Organismal Injury and Abnormalities	Development of adenocarcinoma	3.33E-15	1.522	ABCB11,ABCC3,ABCF	684
75	Gastrointestinal Disease,Organismal Injury and Abnormalities	Colorectal disorder	3.73E-15	-0.935	ABCC3,ABCF2,ABLIM	752
76	Cancer,Organismal Injury and Abnormalities	Advanced malignant tumor	8.5E-15	0.791	ABCC3,ABLIM1,AFAP1	216
77	Digestive System Development and Function,Gastrointestinal Disease	Abnormal morphology of digestive system	2.07E-14		ABCB11,AGR2,AGTR2	126
78	Cancer,Organismal Injury and Abnormalities	Melanoma	3.23E-14	-0.651	A1CF,ABCB11,ABCC3	969
79	Cancer,Organismal Injury and Abnormalities,Reproductive System Disease	Uterine tumor	6.56E-14		A1CF,ACSL1,ACSM3,A	505
80	Cancer,Organismal Injury and Abnormalities,Reproductive System Disease	Female genital tract adenocarcinoma	6.6E-14		ABCC3,ABCF2,ABLIM	525
81	Cellular Movement	Migration of cells	1.03E-13	-0.95	ACE2,ADARB1,ADCY	364
82	Cancer,Gastrointestinal Disease,Organismal Injury and Abnormalities	Colorectal tumor	1.06E-13	1.497	ABCC3,ABCF2,ABLIM	725
83	Cancer,Organismal Injury and Abnormalities	Pelvic adenocarcinoma	2.19E-13		A1CF,ABCC3,ABCF2,A	733
84	Inflammatory Response	Inflammation of absolute anatomical region	2.44E-13	-1.028	ABCB11,ACE2,ACKR4	223
85	Cancer,Organismal Injury and Abnormalities	Serous adenocarcinoma	3.39E-13		ABLIM2,ACTG2,ADCY	171
86	Cancer,Organismal Injury and Abnormalities	Advanced malignant solid tumor	4.13E-13	0.998	ABCC3,AFAP1L1,AGR	153
87	Cancer,Organismal Injury and Abnormalities,Reproductive System Disease	Uterine cancer	5.5E-13		A1CF,ACSL1,ACSM3,A	482
88	Cancer,Organismal Injury and Abnormalities	Extraadrenal retroperitoneal tumor	1.03E-12	0.17	ABCB11,ABCC3,ABO,A	507
89	Cancer,Gastrointestinal Disease,Organismal Injury and Abnormalities	Colorectal cancer	1.08E-12	1.913	ABCC3,ABCF2,ABLIM	708
90	Inflammatory Response	Inflammation of body cavity	1.81E-12	-1.166	ABCB11,ACE2,AGER,A	189
91	Endocrine System Disorders,Organismal Injury and Abnormalities,Reproductive System	Gonadal tumor	2.19E-12	0.647	ABCC3,ABCF2,ABLIM	301
92	Cancer,Organismal Injury and Abnormalities,Respiratory Disease	Respiratory system tumor	3.66E-12	1.322	ABCB11,ABCC3,ABO,A	460
93	crine System Disorders,Organismal Injury and Abnormalities,Reproductive System Dis	Ovarian lesion	5.58E-12	0.816	ABCC3,ABCF2,ABLIM	290
94	Endocrine System Disorders,Organismal Injury and Abnormalities,Reproductive System	Ovarian tumor	8E-12	0.447	ABCC3,ABCF2,ABLIM	289
95	Dermatological Diseases and Conditions,Organismal Injury and Abnormalities	Skin lesion	8.77E-12	0.113	A1CF,ABCB11,ABCC3	877
96	Endocrine System Disorders,Organismal Injury and Abnormalities,Reproductive System	Ovarian cancer	8.8E-12		ABCC3,ABCF2,ABLIM	283
97	Cancer,Organismal Injury and Abnormalities	Advanced extracranial solid tumor	1.02E-11	1.12	ABCC3,ANK3,ANXA8/	125
98	Cancer,Organismal Injury and Abnormalities,Reproductive System Disease	Malignant neoplasm of male genital organ	1.24E-11	0.13	A1CF,ABO,ACSM3,AD.	530
99	Organismal Injury and Abnormalities,Reproductive System Disease	Abnormal growth in endometrium	1.44E-11		A1CF,ACSL1,ACSM3,A	449
100	Cell-To-Cell Signaling and Interaction	Interaction of tumor cell lines	1.63E-11	0.85	ADAM28,APOH,BCAN,	92
101	Organismal Injury and Abnormalities,Reproductive System Disease	Abnormality of endometrium	1.89E-11		A1CF,ACSL1,ACSM3,A	449
102	Cancer,Organismal Injury and Abnormalities,Reproductive System Disease	Male genital neoplasm	2.35E-11	-0.359	A1CF,ABO,ACSM3,AD.	532
103	Cancer,Organismal Injury and Abnormalities	Cancer of cells	2.82E-11	0.579	ABCB11,ABCC3,ABLIN	630
104	Cancer,Organismal Injury and Abnormalities,Reproductive System Disease	Prostate cancer	3E-11	0.762	A1CF,ABO,ACSM3,AD.	522
105	Cancer,Organismal Injury and Abnormalities,Reproductive System Disease	Breast or ovarian carcinoma	3.13E-11	0.423	ABCC3,ABCF2,ABLIM	420
106	Endocrine System Disorders,Organismal Injury and Abnormalities,Reproductive System	Primary ovarian cancer	4.14E-11		AGR2,ANXA10,AOC1,E	26
107	Organismal Survival	Organismal death	5.24E-11	-0.83	ABCB11,ACE2,ACTA1	393
108	Cancer,Organismal Injury and Abnormalities	Malignant solid organ tumor	5.27E-11	-1.13	A1CF,ABCB11,ABCC3	875
109	Cancer,Organismal Injury and Abnormalities,Reproductive System Disease	Prostatic tumor	5.34E-11	-0.599	A1CF,ABO,ACSM3,AD.	523
110	Organismal Survival	Morbidity or mortality	6.21E-11	-0.726	ABCB11,ACE2,ACTA1	397
111	Cell-To-Cell Signaling and Interaction	Binding of tumor cell lines	7E-11	0.731	ADAM28,APOH,BCAN,	88
112	e Disorders,Inflammatory Disease,Organismal Injury and Abnormalities,Skeletal and M	Rheumatic Disease	7.99E-11	-1.805	ACSL1,ACSL5,ACTA1,	225
113	Cancer,Dermatological Diseases and Conditions,Organismal Injury and Abnormalities	Skin tumor	8.45E-11	-1.322	A1CF,ABCB11,ABCC3	864
114	Gastrointestinal Disease,Hepatic System Disease,Organismal Injury and Abnormalities	Liver lesion	9.37E-11	0.931	A1CF,ABCB11,ABCC3	728
115	Cancer,Dermatological Diseases and Conditions,Organismal Injury and Abnormalities	Skin cancer	1.07E-10	-0.6	A1CF,ABCB11,ABCC3	859
116	Cancer,Organismal Injury and Abnormalities	Malignant neoplasm of retroperitoneum	1.11E-10	0.862	ABCB11,ABO,AC0040	455
117	Tissue Development	Growth of epithelial tissue	1.15E-10	-1.11	AGER,AGR2,AGTR2,A	138
118	Cancer,Organismal Injury and Abnormalities,Reproductive System Disease	Endometrium tumor	1.3E-10		A1CF,ACSL1,ACSM3,A	441
119	Endocrine System Disorders,Organismal Injury and Abnormalities,Reproductive System	Advanced ovarian carcinoma	1.3E-10		ANXA8/ANXA8L1,ARL	37
120	Protein Synthesis	Metabolism of cellular protein	1.33E-10		AFP,APOA1,APOA2,AF	47
121	Cancer,Organismal Injury and Abnormalities,Reproductive System Disease	Endometrial cancer	1.43E-10		A1CF,ACSL1,ACSM3,A	440
122	Tissue Morphology	Quantity of cells	1.76E-10	0.37	ACKR4,ADCYAP1,AGE	309
123	Cellular Growth and Proliferation,Tissue Development	Proliferation of epithelial cells	1.86E-10	-1.518	AGR2,AGTR2,AREG,A	104
124	Endocrine System Disorders,Organismal Injury and Abnormalities,Reproductive System	Advanced ovarian cancer	1.9E-10		ANXA8/ANXA8L1,ARL	39
125	Immunological Disease	Systemic autoimmune syndrome	1.96E-10	0.164	ACSL1,ACSL5,ACTA1,	231
126	Cancer,Organismal Injury and Abnormalities	Squamous cell tumor	2.4E-10	0.96	ABCB11,ABCC3,ABO,A	474
127	Endocrine System Disorders,Organismal Injury and Abnormalities,Reproductive System	Advanced ovarian adenocarcinoma	2.55E-10		ANXA8/ANXA8L1,ARL	33
128	gestive System Development and Function,Organ Morphology,Organismal Developme	Morphology of intestine	2.74E-10		AGR2,AP1M2,AREG,B	59
129	Endocrine System Disorders,Organismal Injury and Abnormalities,Reproductive System	Serous ovarian adenocarcinoma	3.09E-10		ANXA8/ANXA8L1,ARL	55
130	Cancer,Organismal Injury and Abnormalities	Squamous-cell carcinoma	3.37E-10	1.119	ABCB11,ABCC3,ABO,A	473
131	Lipid Metabolism,Small Molecule Biochemistry	Metabolism of acylglycerol	3.79E-10	0.599	ACSL1,ACSL5,ALDH1.	40
132	Cancer,Organismal Injury and Abnormalities	Primary tumor	4.11E-10		AFAP1L1,AGR2,ANXA	86
133	Organismal Injury and Abnormalities	Benign lesion	4.16E-10	-0.192	ADARB1,AGER,AJUBA	173
134	Cancer,Dermatological Diseases and Conditions,Organismal Injury and Abnormalities	Cutaneous melanoma	5.08E-10		A1CF,ABCB11,ABCC3	816
135	nd Function,Gastrointestinal Disease,Organ Morphology,Organismal Development,Org	Abnormal morphology of intestine	5.44E-10		AGR2,AP1M2,AREG,C	53
136	Cancer,Organismal Injury and Abnormalities	Lymphohematopoietic cancer	6.93E-10	-0.338	A1CF,ABCB11,ABCC3	464
137	Cancer,Organismal Injury and Abnormalities,Reproductive System Disease	Uterine serous papillary cancer	7.3E-10		ACTG2,ALDH1A1,B3G	51
138	Cellular Movement	Invasion of cells	7.37E-10	0.027	ABLIM1,AFAP1L1,AGE	189
139	Gastrointestinal Disease,Inflammatory Disease	Inflammatory Bowel Disease	7.82E-10	1.628	ACSL1,AGER,B3GAT1	85
140	Cancer,Organismal Injury and Abnormalities	Thoracic neoplasm	7.94E-10	1.705	ABCB11,ABCC3,ABO,A	391
141	Cellular Development,Tissue Development	Differentiation of epithelial tissue	9.56E-10	1.571	AGER,AQP3,BMP4,BM	84
142	Cancer,Organismal Injury and Abnormalities,Respiratory Disease	Lung tumor	1.11E-09	1.467	ABCB11,ABCC3,ABO,A	387
143	Cancer,Organismal Injury and Abnormalities	Lymphohematopoietic neoplasia	1.17E-09	-0.162	A1CF,ABCB11,ABCC3	468
144	Cancer,Organismal Injury and Abnormalities	Advanced adenocarcinoma	1.19E-09		ABCC3,ANXA8/ANXA8	53
145	Cellular Development,Tissue Development	Differentiation of epithelial cells	1.21E-09	2.024	AGER,AQP3,BMP4,BR	74
146	Cancer,Organismal Injury and Abnormalities	Secondary tumor	1.23E-09	0.703	ABCC3,ABLIM1,AFAP1	182
147	cer,Gastrointestinal Disease,Hepatic System Disease,Organismal Injury and Abnormal	Liver tumor	1.49E-09	2.016	A1CF,ABCB11,ABCC3	696
148	Cancer,Organismal Injury and Abnormalities,Reproductive System Disease	Mammary tumor	1.78E-09	0.439	ABLIM1,ABO,ADAM28,	365
149	Cardiovascular Disease,Organismal Injury and Abnormalities	Vaso-occlusion	1.85E-09	-0.652	ABO,AGER,ANGPTL3,	105
150	Cancer,Gastrointestinal Disease,Organismal Injury and Abnormalities	Pancreatobiliary tumor	1.93E-09	0.035	ABCB11,ABCC3,ABO,A	438

151	Cancer,Organismal Injury and Abnormalities	Intrathoracic malignant tumor	2.06E-09	1.507	ABCB11,ABCC3,ABO,	380
152	Organismal Injury and Abnormalities,Reproductive System Disease	Benign pelvic disease	2.23E-09		ADARB1,ALDH1A1,AL	110
153	Cancer,Gastrointestinal Disease,Organismal Injury and Abnormalities	Hepatobiliary carcinoma	2.29E-09	2.282	A1CF,ABCB11,ABCC3	619
154	Cancer,Hematological Disease,Organismal Injury and Abnormalities	Hematopoietic neoplasm	2.32E-09	-0.162	A1CF,ABCB11,ABCC3	464
155	Cancer,Hematological Disease,Organismal Injury and Abnormalities	Hematologic cancer	2.59E-09	-0.338	A1CF,ABCB11,ABCC3	458
156	Cellular Development,Cellular Growth and Proliferation	Cell proliferation of tumor cell lines	2.62E-09	0.988	ABLIM1,ACSL5,ADAM	293
157	Lipid Metabolism,Molecular Transport,Small Molecule Biochemistry	Concentration of triacylglycerol	2.64E-09	0.848	ACSL1,ADCYAP1,AGT	71
158	Cancer,Organismal Injury and Abnormalities,Respiratory Disease	Lung carcinoma	2.69E-09	0.51	ABCB11,ABCC3,ABO,	353
159	Cancer,Organismal Injury and Abnormalities,Respiratory Disease	Lung cancer	2.71E-09	1.37	ABCB11,ABCC3,ABO,	377
160	Cancer,Organismal Injury and Abnormalities	Primary solid tumor	2.77E-09		AFAP1L1,AGR2,ANXA	83
161	cer,Gastrointestinal Disease,Hepatic System Disease,Organismal Injury and Abnormal	Hepatobiliary system cancer	2.99E-09	1.774	A1CF,ABCB11,ABCC3	637
162	Cell-To-Cell Signaling and Interaction	Adhesion of tumor cell lines	3.21E-09	1.266	ADAM28,APOH,BCAN,	69
163	Cancer,Hematological Disease,Organismal Injury and Abnormalities	Neoplasia of blood cells	3.25E-09	-0.655	A1CF,ABCB11,ABCC3	459
164	Lipid Metabolism,Molecular Transport,Small Molecule Biochemistry	Concentration of lipid	3.29E-09	0.856	ABCB11,ABCC3,ACSE	151
165	Lipid Metabolism,Molecular Transport,Small Molecule Biochemistry	Concentration of acylglycerol	4.03E-09	0.418	ACSL1,ADCYAP1,AGT	75
166	Gastrointestinal Disease,Inflammatory Response	Inflammation of gastrointestinal tract	4.09E-09	-1.518	AGR2,APOA4,APOBEC	96
167	Cancer,Organismal Injury and Abnormalities	Benign Tumors	4.67E-09	0.208	ADARB1,AGER,AJUBA	152
168	Cancer,Organismal Injury and Abnormalities,Reproductive System Disease	Uterine carcinoma	5.04E-09		A1CF,ACSL1,ACSM3,	430
169	er,Endocrine System Disorders,Gastrointestinal Disease,Organismal Injury and Abnorm	Pancreatic tumor	5.4E-09	-0.181	ABCB11,ABCC3,ABO,	388
170	Gastrointestinal Disease,Organismal Injury and Abnormalities	Abnormality of large intestine	5.89E-09	-1.182	AGR2,APOA4,AREG,B	85
171	Cancer,Organismal Injury and Abnormalities,Reproductive System Disease	Breast cancer	6.74E-09	-0.048	ABLIM1,ABO,ADAM28,	344
172	Lipid Metabolism,Small Molecule Biochemistry	Metabolism of triacylglycerol	6.81E-09	1.269	ACSL1,ACSL5,ALDH1.	31
173	Cancer,Gastrointestinal Disease,Organismal Injury and Abnormalities	Colon tumor	7.48E-09	0.792	ABCC3,ABCF2,ABLIM	608
174	Cancer,Organismal Injury and Abnormalities	Benign solid tumor	8.02E-09	0.208	ADARB1,AGER,AJUBA	150
175	Cardiovascular Disease	Occlusion of blood vessel	8.48E-09	-0.562	ABO,AGER,ANGPTL3,	102
176	Cancer,Gastrointestinal Disease,Organismal Injury and Abnormalities	Development of digestive organ tumor	9.12E-09	1.379	ABCC3,ABCF2,ABLIM	677
177	Cancer,Organismal Injury and Abnormalities,Renal and Urological Disease	Urinary tract cancer	1.07E-08		AC0040692,ADAM28,	314
178	Development,Cellular Growth and Proliferation,Organismal Injury and Abnormalities,Tu	Proliferation of tumor cells	1.16E-08	1.092	ADAM28,APCDD1,ARE	89
179	Cancer,Organismal Injury and Abnormalities	Primary neoplasm	1.37E-08	0.11	AFAP1L1,AGR2,ANXA	94
180	Cell-To-Cell Signaling and Interaction	Activation of cells	1.57E-08	-0.798	ACSL5,ADCYAP1,AFP	174
181	Dermatological Diseases and Conditions,Organismal Injury and Abnormalities	Exanthem of skin	0.000000016		C3,C4A/C4B,CCL20,C	37
182	Neurological Disease,Organismal Injury and Abnormalities	Epilepsy	1.69E-08	0.282	ADCYAP1,ALG13,ANC	74
183	Cancer,Gastrointestinal Disease,Organismal Injury and Abnormalities	Colon cancer	1.81E-08	1.109	ABCC3,ABCF2,ABLIM	599
184	Neurological Disease	Epilepsy or neurodevelopmental disorder	2.17E-08	-0.262	ADCYAP1,ALG13,ANC	91
185	cer,Gastrointestinal Disease,Hepatic System Disease,Organismal Injury and Abnormal	Liver cancer	2.22E-08	1.626	A1CF,ABCB11,ABCC3	600
186	atological Diseases and Conditions,Organ Morphology,Organismal Injury and Abnorm	Abnormal morphology of skin	2.24E-08		APOA1,AREG,BCL2L1	52
187	Cancer,Organismal Injury and Abnormalities,Respiratory Disease	Non-small cell lung carcinoma	0.000000023	1.938	ABCB11,ABCC3,ACE2	307
188	Cellular Movement	Cell movement of tumor cell lines	2.45E-08	0.151	ACKR4,ADAMTS5,AGE	182
189	Cardiovascular Disease,Organismal Injury and Abnormalities	Occlusion of artery	0.000000025	-0.562	AGER,ANGPTL3,APOA	99
190	cer,Gastrointestinal Disease,Hepatic System Disease,Organismal Injury and Abnormal	Liver carcinoma	2.75E-08	2.123	A1CF,ABCB11,ABCC3	580
191	Cell Death and Survival	Necrosis	2.87E-08	-0.273	ABCC3,ACE2,ACSL1,	410
192	Cancer,Organismal Injury and Abnormalities,Renal and Urological Disease	Urinary tract tumor	2.89E-08		AC0040692,ADAM28,	318
193	Cancer,Organismal Injury and Abnormalities	Lymphoreticular neoplasm	2.98E-08	-0.061	A1CF,ABCB11,ABCC3	405
194	Digestive System Development and Function	Development of digestive system	3.24E-08	2.2	AGR2,ALDH1A1,AQP3	82
195	Inflammatory Disease,Inflammatory Response,Organismal Injury and Abnormalities,Sk	Inflammation of joint	3.93E-08	-1.614	ACSL1,ACTA1,ADAMT	172
196	Cancer,Organismal Injury and Abnormalities	Extrapulmonary squamous cell carcinoma	4.01E-08	-0.577	ABCB11,ABCC3,ABO,	427
197	eases and Conditions,Inflammatory Disease,Inflammatory Response,Organismal Injury	Dermatitis	4.07E-08	0.02	ACKR4,AGER,AREG,B	82
198	Cancer,Organismal Injury and Abnormalities	Advanced stage carcinoma	4.39E-08		ABCC3,ANXA8/ANXA8	61
199	Organismal Injury and Abnormalities,Tissue Morphology	Abnormal morphology of epithelial tissue	4.39E-08		ABCB11,ADCYAP1,AG	82
200	Dermatological Diseases and Conditions,Organismal Injury and Abnormalities	Plaque psoriasis	4.46E-08		CCL20,CSTA,CXCL8,	33
201	ective Tissue Disorders,Organismal Injury and Abnormalities,Skeletal and Muscular Dis	Non-traumatic arthropathy	4.81E-08	0.207	ACSL1,ACTA1,ADAMT	150
202	Endocrine System Disorders,Organismal Injury and Abnormalities,Reproductive System	Ovarian serous tumor	5.01E-08		ABLIM2,ADCY2,AHNA	125
203	Hematological System Development and Function,Tissue Morphology	Quantity of leukocytes	5.16E-08	-0.938	ACKR4,ADCYAP1,AGE	162
204	Cancer,Gastrointestinal Disease,Organismal Injury and Abnormalities	Upper gastrointestinal tract cancer	6.12E-08	0	ADAM28,ADARB1,AD	416
205	Cancer,Gastrointestinal Disease,Organismal Injury and Abnormalities	Upper gastrointestinal tract tumor	6.59E-08	-0.571	ADAM28,ADARB1,AD	418
206	Neurological Disease,Organismal Injury and Abnormalities	Epileptic seizure	6.76E-08	0.152	ADCYAP1,ANOS1,BA	45
207	Embryonic Development,Organismal Development	Development of abdomen	6.97E-08	1.268	ADCYAP1,AGER,AGT	112
208	Digestive System Development and Function	Development of gastrointestinal tract	7.08E-08	1.664	AGR2,AQP3,BCL2L11,	60
209	Endocrine System Disorders,Organismal Injury and Abnormalities,Reproductive System	Advanced serous ovarian adenocarcinoma	7.19E-08		ANXA8/ANXA8L1,ARL	28
210	Cancer,Organismal Injury and Abnormalities,Respiratory Disease	Development of lung tumor	8.19E-08	1.856	ABCB11,ABCC3,ACE2	254
211	Organ Morphology	Abnormal morphology of gland	9.56E-08		AGR2,AGTR2,ALDH1A	64
212	Cancer,Gastrointestinal Disease,Organismal Injury and Abnormalities	Upper gastrointestinal carcinoma	0.000000103		ADAM28,ADARB1,AD	397
213	Cancer,Organismal Injury and Abnormalities,Reproductive System Disease	Prostatic carcinoma	0.000000107	1	A1CF,ABO,ACSM3,AD.	439
214	Embryonic Development,Organismal Development	Development of body trunk	0.000000112	1.29	ACE2,ADAP2,ADCYA	205
215	Immunological Disease	Hypersensitive reaction	0.000000114	0.318	ACKR4,AGER,BCL2L1	82
216	Cancer,Organismal Injury and Abnormalities	Metastatic solid tumor	0.000000116	0.851	ABCC3,AFAP1L1,AGR	120
217	Gastrointestinal Disease,Inflammatory Disease	Crohn disease	0.000000126		ACSL1,AGER,B3GAT1	58
218	cal Disease,Inflammatory Disease,Inflammatory Response,Organismal Injury and Abno	Rheumatoid arthritis	0.00000013	0.077	ACSL1,ACTA1,ADCY8	131
219	Cellular Movement	Invasion of tumor cell lines	0.000000131	-0.004	ABLIM1,AFAP1L1,AGE	149
220	Cancer,Organismal Injury and Abnormalities	Endometrioid carcinoma	0.000000139		ABCC3,ABCF2,ACE2,	419
221	Gastrointestinal Disease,Organismal Injury and Abnormalities	Gastric lesion	0.000000144	-0.708	ADAM28,ADARB1,AD	236
222	Cancer,Organismal Injury and Abnormalities	Ductal carcinoma	0.00000015	1.709	ABCB11,ABO,ACAP1,	377
223	Cancer,Organismal Injury and Abnormalities,Reproductive System Disease	HER2-negative breast cancer	0.000000153		AREG,BRCA1,CAV1,C	43
224	Molecular Transport	Quantity of metal	0.00000016	-0.396	ADCYAP1,APOC3,ASI	92
225	Cancer,Organismal Injury and Abnormalities	HER2 negative cancer	0.000000163		AREG,BRCA1,CAV1,C	44
226	Cancer,Organismal Injury and Abnormalities,Reproductive System Disease	Endometrial carcinoma	0.000000164		A1CF,ACSL1,ACSM3,	397
227	Gastrointestinal Disease,Inflammatory Disease	Gastroenteritis	0.000000167	-1.647	AGR2,APOA4,APOH,A	84
228	Cancer,Gastrointestinal Disease,Organismal Injury and Abnormalities	Gastroesophageal cancer	0.000000176	0	ADAM28,ADARB1,AD	286
229	Hematological System Development and Function,Tissue Morphology	Quantity of blood cells	0.000000182	-0.977	ACKR4,ADCYAP1,AGE	176
230	Cancer,Organismal Injury and Abnormalities	HER2 negative solid tumor	0.000000183		AREG,BRCA1,CAV1,C	44
231	Cardiovascular System Development and Function,Organismal Development	Angiogenesis	0.000000261	0.486	ACE2,AGER,AGTR2,A	165
232	Dermatological Diseases and Conditions,Organismal Injury and Abnormalities	Disorder of hair	0.000000287	-2.431	APCDD1,AREG,BCL2	47
233	Organ Morphology	Morphology of skin	0.000000292		ACE2,APOA1,AREG,A	61
234	Cellular Movement	Homing of cells	0.000000297	-0.804	ACKR4,AFP,AGER,AN	106
235	ocrine System Development and Function,Molecular Transport,Small Molecule Biochem	Concentration of hormone	0.000000301	1.437	ACSBG1,ADCYAP1,AF	75
236	Cancer,Organismal Injury and Abnormalities,Respiratory Disease	Development of lung carcinoma	0.000000304	1	ABCB11,ABCC3,ACE2	248
237	Cardiovascular System Development and Function,Organismal Development	Vasculogenesis	0.000000305	0.454	ACE2,AGER,AGTR2,A	138
238	Development,Cellular Growth and Proliferation,Organismal Injury and Abnormalities,Tu	Proliferation of cancer cells	0.000000314	1.405	ADAM28,APCDD1,ARE	64
239	Neurological Disease,Organismal Injury and Abnormalities	Seizure disorder	0.000000325	-0.549	ADARB1,ADCYAP1,AL	97
240	Cellular Function and Maintenance	Cellular homeostasis	0.000000328	-0.188	ABCC3,ACAP1,ACSL1	259
241	Lipid Metabolism,Molecular Transport,Small Molecule Biochemistry	Quantity of steroid	0.000000329	0.891	ABCB11,ABCC3,ACSE	88
242	Cancer,Gastrointestinal Disease,Organismal Injury and Abnormalities	Stomach tumor	0.000000339	-0.685	ADAM28,ADARB1,AD	233
243	Organismal Injury and Abnormalities,Reproductive System Disease	Endometriosis	0.000000388		ALDH1A3,ANK3,BRCA	73
244	Cancer,Gastrointestinal Disease,Organismal Injury and Abnormalities	Gastric cancer	0.000000424		ADAM28,ADARB1,AD	230
245	Cancer,Gastrointestinal Disease,Organismal Injury and Abnormalities	Development of intestinal tumor	0.000000435		ABCC3,ABCF2,ABLIM	590
246	ective Tissue Disorders,Organismal Injury and Abnormalities,Skeletal and Muscular Dis	Abnormality of cartilage tissue	0.000000437	0.388	ADAMTS5,BMPER,CA	41
247	stinal Disease,Inflammatory Disease,Inflammatory Response,Organismal Injury and Ab	Enteritis	0.000000484	-1.558	AGR2,APOA4,APOH,A	79
248	Cancer,Organismal Injury and Abnormalities,Respiratory Disease	Lung adenocarcinoma	0.000000487	1.414	ABCB11,ABCC3,ACE2	246
249	Organismal Injury and Abnormalities	Ulcer	0.00000049	-1.109	AGER,APOA1,AREG,C	31
250	stinal Disease,Inflammatory Disease,Inflammatory Response,Organismal Injury and Ab	Inflammation of large intestine	0.000000492	-1.182	AGR2,APOA4,AREG,B	74
251	stinal Disease,Inflammatory Disease,Inflammatory Response,Organismal Injury and Ab	Colitis	0.00000052	-1.054	AGR2,APOA4,AREG,B	73
252	Cancer,Gastrointestinal Disease,Organismal Injury and Abnormalities	Development of colorectal tumor	0.000000525		ABCC3,ABCF2,ABLIM	589
253	er,Endocrine System Disorders,Gastrointestinal Disease,Organismal Injury and Abnorm	Pancreatic cancer	0.000000558	0.518	ABCB11,ABO,ACAP1,	331
254	Cancer,Dermatological Diseases and Conditions,Organismal Injury and Abnormalities	Hyperplasia of epidermis	0.000000582	-1.432	AGER,AREG,CAV1,CD	28
255	Neurological Disease,Organismal Injury and Abnormalities	Seizures	0.000000589	-0.686	ADARB1,ADCYAP1,AL	87
256	Cancer,Organismal Injury and Abnormalities	Papillary adenocarcinoma	0.000000593		ABCC3,ANXA8/ANXA8	66
257	Cancer,Organismal Injury and Abnormalities	Papillary carcinoma	0.000000611		ABCC3,ANXA8/ANXA8	67
258	Cardiovascular System Development and Function	Development of vasculature	0.000000642	0.519	ACE2,AGER,AGTR2,A	181
259	Cellular Movement	Chemotaxis	0.000000664	-0.485	ACKR4,AFP,AGER,AN	100
260	Organ Morphology	Morphology of gland	0.00000068		AGR2,AGTR2,ALDH1A	79
261	Cardiovascular Disease,Organismal Injury and Abnormalities	Arteriosclerosis	0.00000072	-0.906	AGER,ANGPTL3,APOA	85
262	Respiratory Disease	Lower respiratory tract disorder	0.000000766	-0.516	ABCC3,AGER,ANKS1E	76
263	Cancer,Gastrointestinal Disease,Organismal Injury and Abnormalities	Colorectal carcinoma	0.00000081		ABCC3,ABCF2,ABLIM	587
264	ncer,Hematological Disease,Immunological Disease,Organismal Injury and Abnormalit	Leukemia	0.000000821	0.464	A1CF,ABCB11,ABCC3	310
265	Cancer,Organismal Injury and Abnormalities,Reproductive System Disease	Hormone receptor negative breast cancer	0.000000827		AREG,BRCA1,CAV1,C	38
266	Cancer,Dermatological Diseases and Conditions,Organismal Injury and Abnormalities	Hyperplasia of skin	0.000000877	-1.368	AGER,AREG,CAV1,CD	32
267	Cancer,Organismal Injury and Abnormalities,Reproductive System Disease	Progesterone receptor negative breast tumor	0.000000878		AREG,BRCA1,CAV1,C	39
268	Cancer,Organismal Injury and Abnormalities	Growth of malignant tumor	0.000000887	0.627	ADAM28,APCDD1,AP	81
269	Cell Death and Survival	Apoptosis	0.000000941	-0.119	ABO,ACE2,ACSL1,AD	399
270	Digestive System Development and Function,Hepatic System Development and Functic	Differentiation of hepatocytes	0.000000956	-0.685	BMP4,E2F7,FGF1,FOS	10
271	Skeletal and Muscular Disorders	Abnormality of limb	0.000000956	-0.893	ATL1,BMP4,BMPER,C	64
272	Cancer,Organismal Injury and Abnormalities,Reproductive System Disease	Prostatic adenocarcinoma	0.000000973		A1CF,ABO,ACSM3,AD.	415
273	Cell-To-Cell Signaling and Interaction,Cellular Assembly and Organization	Cell-cell contact	0.00000098	1.037	AJUBA,AMPH,ANK3,A	114
274	Cardiovascular System Development and Function,Cellular Movement	Cell movement of endothelial cells	0.000000985	0.093	ANGPTL3,APOH,ARH	73
275	Molecular Transport	Quantity of metal ion	0.00000108	-0.333	ADCYAP1,APOC3,ASI	80
276	Endocrine System Disorders,Organismal Injury and Abnormalities,Reproductive System	Ovarian adenocarcinoma	0.00000114		ABCC3,ABCF2,ABLIM	222
277	Cell-To-Cell Signaling and Interaction	Intercellular communication	0.00000121		ADCYAP1,ASIP,CCL2	35
278	ncer,Hematological Disease,Immunological Disease,Organismal Injury and Abnormalit	Acute myeloid leukemia	0.00000123	0.059	A1CF,ABCB11,ABCC3	242
279	orders,Organismal Injury and Abnormalities,Skeletal and Muscular Disorders,Skeletal a	Abnormal morphology of bone	0.00000123		AGTR2,AREG,BMP4,B	80
280	nd Function,Gastrointestinal Disease,Organ Morphology,Organismal Development,Org	Abnormal morphology of small intestine	0.00000123		AGR2,AP1M2,AREG,C	25
281	ncer,Hematological Disease,Immunological Disease,Organismal Injury and Abnormalit	Myeloid leukemia	0.00000126	0.864	A1CF,ABCB11,ABCC3	247
282	Cellular Movement	Migration of tumor cell lines	0.00000129	-0.067	AGER,AREG,ARHGAP	150
283	Digestive System Development and Function,Gastrointestinal Disease	Abnormal morphology of midgut	0.00000134		AGR2,AP1M2,AREG,C	26
284	lular Movement,Hematological System Development and Function,Immune Cell Traffick	Cell movement of granulocytes	0.00000139	-0.771	ACE2,ADCYAP1,AGEF	73
285	Cancer,Organismal Injury and Abnormalities,Renal and Urological Disease	Bladder cancer	0.00000142		ADCY8,AHNAK2,AJUE	185
286	cer,Gastrointestinal Disease,Hepatic System Disease,Organismal Injury and Abnormal	Hepatocellular carcinoma	0.00000147	2.123	ABCB11,ACSM3,ADAM	144
287	System Disorders,Gastrointestinal Disease,Metabolic Disease,Organismal Injury and Ab	Diabetes mellitus	0.00000153	0.339	ACE2,ACSL1,ACSM3,	198
288	Cardiovascular Disease,Organismal Injury and Abnormalities	Atherosclerosis	0.00000155	-0.747	AGER,ANGPTL3,APOA	83
289	Cellular Movement,Immune Cell Trafficking	Leukocyte migration	0.00000156	-1.555	ACE2,ADCYAP1,ADGF	163
290	Tissue Development	Development of epithelial tissue	0.00000164	0.63	AGER,AGTR2,APOA1,	97
291	Organismal Injury and Abnormalities,Tissue Morphology	Size of lesion	0.00000167		ADCYAP1,AGER,APO	70
292	Cellular Assembly and Organization,Cellular Function and Maintenance	Organization of cytoskeleton	0.00000179	0.331	ABLIM1,ACAP1,ADAP	228
293	Lipid Metabolism,Molecular Transport,Small Molecule Biochemistry	Secretion of triacylglycerol	0.00000179	0.024	APOA1,APOA2,APOA4	12
294	Lipid Metabolism,Small Molecule Biochemistry,Vitamin and Mineral Metabolism	Metabolism of terpenoid	0.00000179	1.43	ADCYAP1,ADH1C,AFF	62
295	Cellular Movement	Cell movement of blood cells	0.00000189	-1.601	ACE2,ADCYAP1,ADGF	164
296	Neurological Disease	Progressive neurological disorder	0.00000196	0.508	ACTN3,ADH1C,AGER,	164
297	Cancer,Organismal Injury and Abnormalities,Reproductive System Disease	R2 negative hormone receptor negative breast ca	0.00000198		AREG,BRCA1,CAV1,C	37
298	Cancer,Organismal Injury and Abnormalities,Reproductive System Disease	Endometrial adenocarcinoma	0.000002		ACSL1,ACSM3,ADCY2	347
299	Cellular Function and Maintenance	Ion homeostasis of cells	0.00000206	1.024	ABCC3,ADCY8,ADCY	103
300	lular Movement,Hematological System Development and Function,Immune Cell Traffick	Cell movement of leukocytes	0.00000225	-1.495	ACE2,ADCYAP1,AGEF	138

301	t,Hair and Skin Development and Function,Organ Development,Organismal Developm	Formation of epidermis	0.00000231	-1.054	APCDD1,BNC1,CDH1,	38
302	Carbohydrate Metabolism	Metabolism of carbohydrate	0.00000232	1.254	ADCY2,ADCYAP1,AGT	119
303	Cancer,Organismal Injury and Abnormalities	Head and neck squamous cell carcinoma	0.00000241		ABCC3,ABO,ADAM28,	329
304	System Development and Function,Lymphoid Tissue Structure and Development,Tiss	Morphology of lymphoid tissue	0.00000245		AGR2,ARHGDIB,ARHC	94
305	Lipid Metabolism,Molecular Transport,Small Molecule Biochemistry	Clearance of lipid	0.00000246	0.156	ABCB11,ADH1C,ALDH	14
306	Behavior,Digestive System Development and Function	Feeding	0.00000318	0.295	AGTR2,APOA4,ASIP,A	58
307	nd Function,Gastrointestinal Disease,Organ Morphology,Organismal Development,Org	Abnormal morphology of stomach	0.00000318		AGR2,BRCA1,CA9,CA	19
308	Cellular Movement	Cell movement of myeloid cells	0.00000321	-1.119	ACE2,ADCYAP1,AGEF	102
309	System Development and Function,Hepatic System Disease,Inflammatory Disease,Infl	Inflammation of liver	0.00000361	0.638	ABCB11,APOA1,APOA	64
310	Lipid Metabolism,Molecular Transport,Small Molecule Biochemistry	Concentration of sterol	0.00000361	0.932	ABCB11,ADCYAP1,AN	60
311	llular Growth and Proliferation,Hair and Skin Development and Function,Organ Develop	Proliferation of dermal cells	0.00000366	-1.404	AGTR2,AREG,CASR,C	39
312	Lipid Metabolism,Molecular Transport,Small Molecule Biochemistry	Concentration of cholesterol	0.0000037	1.009	ABCB11,ADCYAP1,AN	58
313	Hair and Skin Development and Function,Organ Development	Growth of skin	0.00000373	-1.148	AGTR2,AREG,BRCA1,	45
314	Cell Signaling,Molecular Transport,Vitamin and Mineral Metabolism	Quantity of Ca2+	0.00000376	-0.216	ADCYAP1,APOC3,ASII	72
315	Cellular Assembly and Organization	Formation of plasma membrane	0.00000393	0.238	BCAN,BHLHB9,CASR,	53
316	ration,Connective Tissue Development and Function,Hair and Skin Development and F	Proliferation of keratinocytes	0.00000397	-1.421	AGTR2,AREG,CASR,C	35
317	t,Hair and Skin Development and Function,Organ Development,Organismal Developm	Formation of skin	0.00000405	0.433	AGTR2,APCDD1,AQP3	79
318	Metabolic Disease,Organismal Injury and Abnormalities	Glucose metabolism disorder	0.00000408	0.3	ACE2,ACSL1,ACSM3,	231
319	Endocrine System Disorders,Organismal Injury and Abnormalities,Reproductive System	Early stage serous ovarian cancer	0.00000414		CLEC3B,MSLN,MUC1,	8
320	Organismal Injury and Abnormalities,Tissue Morphology	Morphology of lesion	0.00000424		ADCYAP1,AGER,APO	74
321	Neurological Disease,Skeletal and Muscular Disorders	Neuromuscular disease	0.00000447	0.379	ACTN3,ADCY2,ADCY7	147
322	Endocrine System Disorders,Organismal Injury and Abnormalities,Reproductive System	Grade 3 primary serous ovarian carcinoma	0.00000451		CLEC3B,MSLN,MUC1,	6
323	Respiratory Disease,Respiratory System Development and Function	Abnormal morphology of respiratory system	0.00000459		ALDH1A1,ALDH1A3,A	55
324	Conditions,Immunological Disease,Inflammatory Disease,Inflammatory Response,Orga	Atopic dermatitis	0.00000461		ACKR4,BRF2,C3,CARI	48
325	llular Growth and Proliferation,Hair and Skin Development and Function,Organ Develop	Proliferation of epidermal cells	0.00000479	-1.577	AGTR2,AREG,CASR,C	37
326	Cancer,Gastrointestinal Disease,Organismal Injury and Abnormalities	Gastro-esophageal carcinoma	0.00000482		ADAM28,ADARB1,ADC	258
327	Cancer,Hematological Disease,Organismal Injury and Abnormalities	Malignant myeloid neoplasm	0.00000482	0.059	A1CF,ABCB11,ABCC3	258
328	Organismal Development	Morphology of head	0.00000502		ADCYAP1,AGTR2,ALD	165
329	Cancer,Organismal Injury and Abnormalities,Renal and Urological Disease	Bladder tumor	0.00000506		ADCY8,AHNAK2,AJUB	186
330	Organismal Injury and Abnormalities	Visceromegaly	0.00000529		ABCB11,ACE2,ACSL1	130
331	Cellular Movement	Migration of hepatoma cell lines	0.00000534	-1.045	CAV1,CDC25B,CXCL8	26
332	ular Development,Embryonic Development,Organismal Development,Tissue Developm	Differentiation of embryonic tissue	0.00000537	2.135	AJUBA,BEX2,BMP4,BF	55
333	Development and Function,Lymphoid Tissue Structure and Development,Organ Morpho	Morphology of lymphoid organ	0.0000054		ARHGDIB,ARHGEF6,A	86
334	Lipid Metabolism,Small Molecule Biochemistry,Vitamin and Mineral Metabolism	Steroid metabolism	0.0000054	1.351	ADCYAP1,AFP,ANGPT	51
335	Cardiovascular System Development and Function,Cellular Movement	Migration of endothelial cells	0.00000556	0.703	ANGPTL3,APOH,ARHC	66
336	tion,Organ Morphology,Organismal Development,Skeletal and Muscular System Devel	Morphology of rib	0.00000584		BMP4,CDX1,CDX2,CR	19
337	Cell-To-Cell Signaling and Interaction,Cellular Assembly and Organization	Stabilization of intercellular junctions	0.00000611	-0.518	AJUBA,ANK3,BCAN,D	9
338	ellular Development,Connective Tissue Development and Function,Tissue Developme	Differentiation of connective tissue cells	0.00000625	1.408	AGER,AREG,ASXL2,A	110
339	Cancer,Organismal Injury and Abnormalities,Reproductive System Disease	Uterine endometrioid carcinoma	0.00000642		ACSL1,ACSM3,ADCY2	334
340	Cell-To-Cell Signaling and Interaction,Cellular Assembly and Organization	Formation of intercellular junctions	0.00000656	0.46	AJUBA,BCAN,BHLHB9	54
341	Endocrine System Disorders,Organismal Injury and Abnormalities,Reproductive System	Ovarian carcinoma	0.00000686		ABCC3,ABCF2,ABLIM2	228
342	Cancer,Hematological Disease,Organismal Injury and Abnormalities	Bone marrow cancer	0.00000701	0.864	A1CF,ABCB11,ABCC3	259
343	Hematological System Development and Function,Tissue Morphology	Quantity of granulocytes	0.00000704	-0.958	AGER,AMBP,ARHGDIE	61
344	unological Disease,Lymphoid Tissue Structure and Development,Organ Morphology,C	Abnormal morphology of lymphoid organ	0.0000071		ARHGDIB,ARHGEF6,B	72
345	Renal and Urological System Development and Function	Morphology of urinary system	0.00000711		ACE2,ACTG2,AGER,A	62
346	Metabolism,Molecular Transport,Small Molecule Biochemistry,Vitamin and Mineral Meta	Quantity of retinoid	0.00000749	-0.618	ADH1C,ALDH1A1,APC	12
347	Cancer,Organismal Injury and Abnormalities	Liquid tumor	0.00000755	0.376	A1CF,ABCB11,ABCC3	319
348	Metabolic Disease,Organismal Injury and Abnormalities	Amyloidosis	0.00000763		AGER,AMPH,APOA1,A	103
349	Digestive System Development and Function	Function of gastrointestinal tract	0.00000793		AGR2,AP1M2,CFTR,CI	13
350	l Signaling and Interaction,Cellular Assembly and Organization,Cellular Function and M	Assembly of intercellular junctions	0.00000808	0.444	BCAN,BHLHB9,CASR,	51
351	Immunological Disease,Inflammatory Disease	Atopic disease	0.00000808	-0.152	ACKR4,BRF2,C3,CARI	51
352	Cancer,Organismal Injury and Abnormalities	Upper aerodigestive tract carcinoma	0.00000814		ADAM28,ADARB1,AHN	299
353	Gastrointestinal Disease,Hepatic System Disease	Cholestasis	0.00000841		ABCB11,ABCC3,ACSL	22
354	Cancer,Neurological Disease,Organismal Injury and Abnormalities	Nervous system neoplasm	0.0000087	0.538	ABCC3,ADAMTS5,ADA	209
355	Neurological Disease,Organismal Injury and Abnormalities,Psychological Disorders	Degenerative dementia	0.00000887		AGER,AMPH,APOA1,A	100
356	Developmental Disorder,Organismal Injury and Abnormalities	Ectodermal dysplasia	0.0000092		CDH1,COL11A1,DSP,I	15
357	Cancer,Organismal Injury and Abnormalities,Reproductive System Disease	Breast carcinoma	0.00000927	0.423	ABLIM1,ABO,ADAM28,	241
358	natological System Development and Function,Inflammatory Response,Tissue Morphol	Quantity of phagocytes	0.0000094	-0.913	AGER,AGR2,AMBP,AP	78
359	Cancer,Organismal Injury and Abnormalities	Neoplasia of tumor cell lines	0.00000942	0.522	ABLIM1,ALDH1A3,ARE	74
360	Gastrointestinal Disease,Organismal Injury and Abnormalities	Abnormality of small intestine	0.00000994		AGR2,AP1M2,AREG,C	29
361	atological Diseases and Conditions,Organ Morphology,Organismal Injury and Abnorm	Redness of skin	0.0000101		CTSE,DGAT1,GBA,IL3	10
362	Cancer,Hematological Disease,Organismal Injury and Abnormalities	Myeloid neoplasm	0.0000102	0.128	A1CF,ABCB11,ABCC3	265
363	atological Diseases and Conditions,Organ Morphology,Organismal Injury and Abnorm	Abnormal morphology of epidermis	0.0000103		AREG,CBS/CBSL,CTS	28
364	Cancer,Gastrointestinal Disease,Organismal Injury and Abnormalities	Gastroesophageal adenocarcinoma	0.0000105		ADAM28,ADARB1,ADC	233
365	Cancer,Organismal Injury and Abnormalities	Growth of tumor	0.0000105	0.084	ADAM28,AFAP1L1,AG	151
366	Molecular Transport	Transport of ion	0.0000107	1.654	ABCC3,ANK3,ANO10,	89
367	ic Disease,Neurological Disease,Organismal Injury and Abnormalities,Psychological D	Alzheimer disease	0.000011		AGER,AMPH,APOA1,A	98
368	Cancer	Hyperplasia of tissue	0.000011	-1.739	AGR2,C3,CA9,CAV1,C	39
369	Respiratory System Development and Function	Morphology of respiratory system	0.0000112		ACE2,ALDH1A1,ALDH	59
370	Organismal Injury and Abnormalities,Renal and Urological Disease	Renal lesion	0.0000112	0.116	AC0040692,ADAM28,	199
371	Neurological Disease,Organismal Injury and Abnormalities,Psychological Disorders	Alzheimer disease or frontotemporal dementia	0.0000117		AGER,AMPH,APOA1,A	99
372	er,Endocrine System Disorders,Gastrointestinal Disease,Organismal Injury and Abnorm	Pancreatic carcinoma	0.0000118	-0.115	ABCB11,ABO,ACAP1,	305
373	Cancer,Organismal Injury and Abnormalities	Malignant neoplasm of aerodigestive tract	0.0000119		ADAM28,ADARB1,AHN	304
374	Carbohydrate Metabolism	Synthesis of carbohydrate	0.0000121	0.961	ADCY2,ADCYAP1,AGT	90
375	ective Tissue Disorders,Organismal Injury and Abnormalities,Skeletal and Muscular Dis	Abnormality of rib cage	0.0000124		BMP4,CDX1,CDX2,CR	25
376	Cancer,Organismal Injury and Abnormalities	Connective or soft tissue tumor	0.0000128	0.39	A1CF,ADAMTS5,ADAF	217
377	Embryonic Development,Organismal Development	Development of head	0.0000135	1.682	ADCYAP1,AGTR2,AK7	166
378	Endocrine System Disorders,Organismal Injury and Abnormalities,Reproductive System	Grade 3 ovarian cancer	0.0000136		CLEC3B,FOXG1,MSLN	8
379	nd Function,Gastrointestinal Disease,Organ Morphology,Organismal Development,Org	Abnormal morphology of duodenum	0.0000136		AP1M2,CFTR,FGA,IHH	8
380	Neurological Disease,Organismal Injury and Abnormalities,Psychological Disorders	Dementia	0.0000138		AGER,AMPH,APOA1,A	105
381	Lipid Metabolism,Molecular Transport,Small Molecule Biochemistry	Uptake of lipid	0.0000142	0.528	ABCC3,ACSL1,ACSL5	34
382	Cancer,Gastrointestinal Disease,Organismal Injury and Abnormalities	Colon carcinoma	0.0000144		ABCC3,ABCF2,ABLIM2	550
383	Organismal Injury and Abnormalities,Respiratory Disease	Lung injury	0.0000144	-0.841	AGER,AMBP,APOH,C3	40
384	Cell-To-Cell Signaling and Interaction	Activation of connective tissue cells	0.0000149	1.454	AGER,APOB,BCL2L11	32
385	Dermatological Diseases and Conditions,Organismal Injury and Abnormalities	Chronic psoriasis	0.000015		AGER,CASP4,CDC25E	28
386	Developmental Disorder,Organismal Injury and Abnormalities	Congenital malformation of genitourinary system	0.0000153	-0.069	ACSL1,AGTR2,ALDH1	73
387	Embryonic Development,Organismal Development,Tissue Development	Development of endoderm	0.0000156		BMP4,CFC1/CFC1B,DI	18
388	Metabolism,Molecular Transport,Small Molecule Biochemistry,Vitamin and Mineral Meta	Concentration of retinol	0.0000157	-0.983	APOA1,APOC3,DGAT1	9
389	Tissue Development	Formation of gland	0.0000159	0.374	APOA1,AREG,BCL2L1	46
390	Cancer,Hematological Disease,Organismal Injury and Abnormalities	Bone marrow neoplasm	0.0000161	0.397	A1CF,ABCB11,ABCC3	262
391	ncer,Hematological Disease,Immunological Disease,Organismal Injury and Abnormalit	Acute leukemia	0.0000161	-0.44	A1CF,ABCB11,ABCC3	253
392	Hair and Skin Development and Function,Organ Morphology	Morphology of hair	0.0000164		AREG,ASIP,CBS/CBSL	25
393	Vitamin and Mineral Metabolism	Quantity of vitamin	0.0000165	-0.692	ABCC3,ADH1C,ALDH	20
394	er,Endocrine System Disorders,Gastrointestinal Disease,Organismal Injury and Abnorm	Pancreatic adenocarcinoma	0.0000167	0.849	ABCB11,ABO,ACAP1,	290
395	Organismal Injury and Abnormalities	Extraintestinal functional disorder	0.0000168	0.273	ALDH1A1,ALDH1A3,A	71
396	Cancer,Organismal Injury and Abnormalities,Tumor Morphology	Invasion of tumor	0.0000171	0.087	AREG,BCAN,CA9,CAV	44
397	Cancer,Organismal Injury and Abnormalities,Reproductive System Disease	Endometrioid endometrial adenocarcinoma	0.0000174		ACSL1,ACSM3,ADCY2	326
398	Cancer,Gastrointestinal Disease,Organismal Injury and Abnormalities	Secondary neoplasm of digestive system	0.000018	0.816	ANK3,APOA1,CAV1,CI	58
399	ogy,Organismal Development,Organismal Injury and Abnormalities,Skeletal and Muscu	Abnormal morphology of rib	0.0000188		BMP4,CDX1,CDX2,CR	18
400	elopment,Digestive System Development and Function,Hepatic System Development a	Differentiation of liver cells	0.0000189	-0.964	BMP4,E2F7,FGF1,FOS	12
401	Cellular Assembly and Organization	Sliding of myofilaments	0.0000189		ACTA1,ACTN3,DES,M	12
402	Cardiovascular Disease,Organismal Injury and Abnormalities	Peripheral vascular disease	0.0000191	0.216	ACE2,ADAP2,ADCY8,	79
403	Neurological Disease,Organismal Injury and Abnormalities,Psychological Disorders	Tauopathy	0.0000199		AGER,AMPH,APOA1,A	100
404	isease,Lymphoid Tissue Structure and Development,Organ Morphology,Organismal De	Abnormal morphology of spleen	0.0000205		BANK1,BCL2L11,BLNI	57
405	Cancer,Organismal Injury and Abnormalities	Lymphatic system tumor	0.0000206	-0.721	AFAP1L1,AGR2,ALDH	281
406	Gastrointestinal Disease,Organismal Injury and Abnormalities	Obstruction of intestine	0.0000212	-1.144	ACTG2,AGR2,CFTR,CI	19
407	elopment and Function,Organ Development,Organismal Development,Tissue Developm	Development of cornea	0.0000214	0.039	ALDH1A1,ALDH1A3,A	14
408	Cell-To-Cell Signaling and Interaction	Adhesion of carcinoma cell lines	0.0000214	0.42	CAV1,CDH1,CHP2,DS	14
409	Cancer,Gastrointestinal Disease,Organismal Injury and Abnormalities	Colorectal adenocarcinoma	0.0000214		ABCC3,ABCF2,ABLIM2	560
410	and Conditions,Hair and Skin Development and Function,Organ Morphology,Organism	Abnormal morphology of hair	0.0000218		AREG,CBS/CBSL,CEL	23
411	Tissue Development	Organization of extracellular matrix	0.0000234	0	ADAMTS5,BCAN,CAPI	42
412	Molecular Transport	Transport of chloride	0.0000235	0.223	ANO10,ANO4,ANO7,AI	17
413	Cellular Development,Cellular Growth and Proliferation	Proliferation of pancreatic cancer cell lines	0.0000239	1.219	CDH17,CEACAM6,CEI	36
414	opment,Organismal Injury and Abnormalities,Skeletal and Muscular Disorders,Skeletal a	Abnormal morphology of rib cage	0.0000242		BMP4,CDX1,CDX2,CR	21
415	Cell-To-Cell Signaling and Interaction,Cellular Growth and Proliferation	Stimulation of cells	0.000025	-1.008	ADCYAP1,APOA1,ASII	56
416	Molecular Transport	Transport of molecule	0.000025	1.687	A1CF,ABCB11,ABCC3	246
417	Cancer,Gastrointestinal Disease,Organismal Injury and Abnormalities	Metastatic malignant neoplasm of digestive systen	0.0000252		ANK3,CAV1,CD36,CDI	59
418	Organismal Injury and Abnormalities,Renal and Urological Disease	Renal impairment	0.0000254	-0.659	AGTR2,APOB,C3,C4A	48
419	Cardiovascular System Development and Function	Neovascularization	0.0000263	-0.571	C3,CAV1,CBS/CBSL,C	34
420	Neurological Disease	Neurological signs	0.0000264	-1.176	ACTN3,ADCY2,ADCY7	114
421	Lipid Metabolism,Molecular Transport,Small Molecule Biochemistry	Concentration of fatty acid	0.0000264	0.771	ACSBG1,ACSL1,ADCY	54
422	Cancer,Organismal Injury and Abnormalities	Upper aero-digestive squamous cell carcinoma	0.0000269		ADAM28,ADARB1,AHN	235
423	Digestive System Development and Function,Organismal Development	Morphology of mouth	0.0000269		AREG,BMP4,CFTR,CR	30
424	Cancer,Gastrointestinal Disease,Organismal Injury and Abnormalities	Gastric carcinoma	0.0000274		ADAM28,ADARB1,ADC	199
425	eases and Conditions,Immunological Disease,Inflammatory Disease,Organismal Injury	Lichen planus	0.0000282		CALML3,CCL20,DEFB	21
426	Gastrointestinal Disease,Hepatic System Disease,Organismal Injury and Abnormalities	Intrahepatic cholestasis	0.0000284		ABCB11,ACSL1,ADH1	17
427	Cancer,Neurological Disease,Organismal Injury and Abnormalities	Central nervous system tumor	0.0000286	0.339	ABCC3,ADAMTS5,ADA	197
428	Carbohydrate Metabolism	Synthesis of polysaccharide	0.0000286	-0.024	ADCY2,B3GAT1,B3GN	41
429	Organismal Development	Abnormal morphology of body wall	0.0000289		BMP4,CDX1,CDX2,CR	23
430	velopment,Skeletal and Muscular Disorders,Skeletal and Muscular System Developme	Abnormal morphology of limb	0.0000291		ATL1,BMP4,BMPER,CI	43
431	Endocrine System Disorders,Organismal Injury and Abnormalities,Reproductive System	Stage I serous ovarian adenocarcinoma	0.0000296		CLEC3B,MSLN,MUC1,	7
432	Cellular Assembly and Organization,Cellular Function and Maintenance	Organization of cytoplasm	0.0000297	0.331	ABLIM1,ACAP1,ADAP	240
433	Cancer,Organismal Injury and Abnormalities,Renal and Urological Disease	Bladder carcinoma	0.00003		ADCY8,AHNAK2,AJUE	165
434	Organ Morphology	Morphology of epidermis	0.0000315		AREG,CASR,CBS/CBS	32
435	Cancer,Organismal Injury and Abnormalities	Invasion of tissue	0.0000317	-0.586	AFAP1L1,ALDH1A1,AL	36
436	Cancer,Gastrointestinal Disease,Organismal Injury and Abnormalities	Advanced colorectal cancer	0.0000322		ANK3,CAV1,CD36,CD	53
437	Cellular Assembly and Organization,Cellular Function and Maintenance	Microtubule dynamics	0.0000322	0.169	ABLIM1,ACAP1,ADAP	191
438	Organismal Survival	Survival of organism	0.0000324	-0.666	ACSL1,ACTG2,ADCYA	125
439	Lipid Metabolism,Small Molecule Biochemistry	Recruitment of lipid	0.0000324	1.954	APOA1,APOA2,PPARG	4
440	Cell Morphology,Embryonic Development	Conversion of embryonic cells	0.0000324		BMP4,DMBT1,FOS,MM	4
441	Dermatological Diseases and Conditions,Organismal Injury and Abnormalities	Loss of hair	0.0000326	-2.431	AREG,BCL2A1,BRCA1	27
442	Cancer,Neurological Disease,Organismal Injury and Abnormalities	Central nervous system solid tumor	0.0000329	0.339	ABCC3,ADAMTS5,ADA	196
443	Cancer,Gastrointestinal Disease,Organismal Injury and Abnormalities	Metastatic colorectal cancer	0.0000341		ANK3,CAV1,CD36,CD	52
444	Cardiovascular Disease,Organismal Injury and Abnormalities	Atherosclerotic lesion	0.0000353	-1.537	APOA1,APOA2,APOA4	34
445	ve Tissue Development and Function,Skeletal and Muscular System Development and	Remodeling of bone	0.0000359	-0.354	BCL2L11,CAV1,CD68,	39
446	Organismal Injury and Abnormalities	Fibrosis	0.0000362	1.412	ACE2,ACSL1,ACTA1,A	101
447	Cancer,Organismal Injury and Abnormalities	Neoplasia of carcinoma cell lines	0.0000371	0.257	ALDH1A3,CAV1,CEAC	24
448	Lipid Metabolism,Small Molecule Biochemistry	Synthesis of acylglycerol	0.0000379	0.685	ACSL1,ACSL5,ALDH1.	23
449	Gastrointestinal Disease,Organismal Injury and Abnormalities	Non-inflammatory intestinal disease	0.0000379	-1.4	ACTG2,AGR2,CFTR,CI	21
450	Cancer,Organismal Injury and Abnormalities	Stage I-II cancer	0.0000382		BPIFA1,CLDN2,CLEC	36
451	Cancer,Organismal Injury and Abnormalities,Renal and Urological Disease	Renal cancer	0.0000386		AC0040692,ADAM28,	180
452	Organismal Development,Organismal Injury and Abnormalities	Abnormal morphology of thoracic cavity	0.0000391		ACE2,ACSL1,ACTG2,	144
453	Organismal Injury and Abnormalities,Renal and Urological Disease	Formation of renal lesion	0.0000397		AC0040692,ADAM28,	164
454	velopmental Disorder,Organismal Injury and Abnormalities,Renal and Urological Disea	Congenital anomalies of kidney and urinary tract	0.0000399	-0.736	ACSL1,AGTR2,ALDH1	56
455	Embryonic Development,Organismal Development	Development of body axis	0.00004	1.682	ADCYAP1,AGTR2,AK7	173
456	ent,Hematological System Development and Function,Immune Cell Trafficking,Inflamm	Cell movement of phagocytes	0.0000407	-0.777	ACE2,ADCYAP1,AGEF	98
457	Cancer	Hyperplasia of epithelial cells	0.0000411	-1.061	C3,CAV1,CD40,ERBB	19
458	l Signaling and Interaction,Cellular Assembly and Organization,Cellular Function and M	Development of gap junctions	0.0000416	-0.193	BCAN,BHLHB9,CAV1,	44
459	ndocrine System Disorders,Gastrointestinal Disease,Organ Morphology,Organismal De	Abnormal morphology of pancreas	0.0000422		AGTR2,CES1,CFTR,DI	24
460	nd Function,Gastrointestinal Disease,Organ Morphology,Organismal Development,Org	Abnormal morphology of large intestine	0.0000429		AGR2,CEACAM1,CFTF	16
461	Cancer,Organismal Injury and Abnormalities,Renal and Urological Disease	Renal cell carcinoma	0.0000431		AC0040692,ADAM28,	163
462	Lipid Metabolism,Small Molecule Biochemistry	Catabolism of acylglycerol	0.0000434		APOA2,APOB,APOC3,	10
463	feration,Connective Tissue Development and Function,Skeletal and Muscular System D	Formation of osteoblasts	0.0000435	0.192	ALDH1A1,DKK1,FRZB	15
464	ndocrine System Disorders,Gastrointestinal Disease,Organismal Injury and Abnormaliti	Pancreatic duct disorder	0.0000442	0.849	ABCB11,ABO,ACAP1,	282
465	opment and Function,Hepatic System Development and Function,Organ Morphology,O	Morphology of liver	0.0000448		ABCB11,ANGPTL3,AP	59
466	Endocrine System Disorders,Organismal Injury and Abnormalities,Reproductive System	Stage 3c primary serous ovarian carcinoma	0.0000451		MSLN,MUC1,MUC16,S	5
467	Endocrine System Disorders,Organismal Injury and Abnormalities,Reproductive System	Stage 1A primary serous ovarian carcinoma	0.0000451		CLEC3B,MUC1,MUC1	5
468	Cell Cycle	Mitogenesis of sarcoma cell lines	0.0000451	0.391	EGF,IGF1,IGFBP2,IGFE	5
469	Cellular Movement	Cellular infiltration	0.0000462	-0.403	ACE2,AGER,APOA1,BI	81
470	lopment and Function,Gastrointestinal Disease,Organismal Development,Organismal I	Abnormal morphology of mouth	0.0000463		AREG,BMP4,CFTR,CR	27
471	Immunological Disease	Allergy	0.0000467	-0.518	ACKR4,AGER,AQP3,B	71
472	Hereditary Disorder,Neurological Disease,Organismal Injury and Abnormalities	Familial encephalopathy	0.0000475		ABLIM1,ACSL1,ACTN3	182
473	Cancer,Organismal Injury and Abnormalities	Visceral metastasis	0.0000482	1.685	ANK3,APOA1,CAV1,CI	72
474	Cellular Development,Embryonic Development,Organismal Development	Differentiation of embryonic cells	0.0000494	2.47	AGER,AJUBA,BAIAP2,	48
475	Organismal Injury and Abnormalities	Edema	0.0000499	1.05	AGER,ATP1A3,C3,CA	65
476	Cancer,Organismal Injury and Abnormalities,Renal and Urological Disease	Renal tumor	0.000051		AC0040692,ADAM28,	183
477	Cancer,Gastrointestinal Disease,Organismal Injury and Abnormalities	Metastatic gastrointestinal tract cancer	0.0000517		ANK3,CAV1,CD36,CD	56
478	Endocrine System Disorders,Organismal Injury and Abnormalities,Reproductive System	Grade 3 ovarian carcinoma	0.0000518		CLEC3B,MSLN,MUC1,	7
479	Hereditary Disorder,Neurological Disease,Organismal Injury and Abnormalities	Peroxisomal acyl CoA oxidase deficiency	0.0000518		CCL26,CCL7,CXCL14	7
480	Organ Morphology	Abnormal morphology of exocrine gland	0.0000519		AGR2,AREG,BRCA1,C	27
481	Organismal Development	Size of body	0.0000523	1.125	ABCB11,ACTA1,ADCY	131
482	llular Movement,Hematological System Development and Function,Immune Cell Traffick	Cellular infiltration by leukocytes	0.0000523	-0.494	ACE2,AGER,APOA1,BI	73
483	nent,Nervous System Development and Function,Organ Development,Organismal Deve	Angiogenesis of eye	0.0000527	0.632	ANGPTL3,CXCL14,CX	14
484	Gastrointestinal Disease,Hepatic System Disease,Organismal Injury and Abnormalities	Cirrhosis of liver	0.0000535		ABCB11,ABCC3,APOA	37
485	Connective Tissue Disorders,Organismal Injury and Abnormalities,Tissue Morphology	Deterioration of connective tissue	0.0000535	0.744	ADAMTS5,AREG,CTSI	37
486	er,Endocrine System Disorders,Gastrointestinal Disease,Organismal Injury and Abnorm	Pancreatic ductal carcinoma	0.0000536	0.849	ABCB11,ABO,ACAP1,	281
487	nismal Injury and Abnormalities,Skeletal and Muscular Disorders,Skeletal and Muscular	Abnormal morphology of skeleton	0.0000539		BMP4,BMPER,CDK10,	49
488	ellular Development,Connective Tissue Development and Function,Tissue Developme	Differentiation of bone	0.0000539	0.449	AREG,ASXL2,BMP4,BI	67
489	Molecular Transport	Transport of anion	0.000054	0.967	ABCC3,ANO10,ANO4,	28
490	Cancer,Gastrointestinal Disease,Organismal Injury and Abnormalities	Advanced malignant gastrointestinal neoplasm	0.0000548		ANK3,CAV1,CD36,CD	57
491	Cell Signaling	Cell surface receptor linked signal transduction	0.0000549	0.628	AKAP7,APOA1,BATF,B	66
492	Neurological Disease,Organismal Injury and Abnormalities	Progressive encephalopathy	0.0000559		ACTN3,ADH1C,AGER,	135
493	Organismal Injury and Abnormalities,Reproductive System Disease	Benign uterine disease	0.0000562		ADARB1,ALDH1A1,AR	47
494	Organismal Injury and Abnormalities,Renal and Urological Disease	Proximal tubular toxicity	0.0000563		BMP4,CRYM,CYP2C9,	19
495	t,Cellular Function and Maintenance,Cellular Growth and Proliferation,Nervous System	Axonogenesis	0.0000566	-0.116	ADGRG6,AGTR2,ANK3	44
496	Endocrine System Development and Function,Small Molecule Biochemistry	Metabolism of hormone	0.0000567	0.785	ADCYAP1,AFP,AREG,I	38
497	Organismal Injury and Abnormalities	Pathological Cyst	0.0000572	-0.558	ADAP1,AGER,ANO9,B	31
498	Digestive System Development and Function,Organismal Development	Morphogenesis of embryonic gastrointestinal trac	0.0000575		FGFR2,GATA4,HLX,HN	8
499	Digestive System Development and Function,Organ Development	Function of intestine	0.0000597		AGR2,AP1M2,CFTR,EI	10
500	Organismal Development,Organismal Injury and Abnormalities	Abnormal morphology of head	0.0000603		ADCYAP1,AGTR2,ALD	143

Table S3 (Related to Figure4). Gene enrichment sets for control vs *$BRCA1^{mut}$* -FTE organoids at 1 year culture

NAME	SIZE	ES	NES	NOM p-val	FDR q-val	FWER p-val	RANK AT MAX	LEADING EDGE
GNF2_EIF3S6	120	0.6727409	2.8466096	0	0	0	5273	tags=82%, list=27%, signal=111%
MORF_ACTG1	138	0.6190265	2.7050114	0	0	0	6618	tags=83%, list=34%, signal=125%
MORF_TPT1	102	0.6477453	2.6736124	0	0	0	5716	tags=81%, list=29%, signal=115%
MORF_NPM1	162	0.6042291	2.647897	0	0	0	6649	tags=83%, list=34%, signal=125%
GCM_TPT1	69	0.6866267	2.6212063	0	0	0	4785	tags=86%, list=25%, signal=113%
GCM_NPM1	115	0.61789876	2.5851052	0	0	0	6596	tags=86%, list=34%, signal=129%
GNF2_ST13	64	0.6888458	2.5632267	0	0	0	5273	tags=84%, list=27%, signal=115%
GNF2_TPT1	38	0.73548156	2.5450826	0	0	0	4761	tags=95%, list=24%, signal=125%
MORF_NME2	154	0.5721919	2.5130274	0	0	0	6659	tags=77%, list=34%, signal=115%
GNF2_MMP1	32	0.7303712	2.4124823	0	0	0	1928	tags=59%, list=10%, signal=66%
MODULE_312	45	0.68017954	2.389409	0	0	0	2563	tags=40%, list=13%, signal=46%
MODULE_83	316	0.49353978	2.3648179	0	0	0	6709	tags=68%, list=34%, signal=102%
GCM_PSME1	84	0.5871864	2.352315	0	0	0	6830	tags=75%, list=35%, signal=115%
MODULE_254	54	0.629287	2.3378637	0	0	0	2563	tags=39%, list=13%, signal=45%
MORF_RAN	266	0.49931005	2.332909	0	0	0	8072	tags=77%, list=41%, signal=129%
MORF_FBL	138	0.5317876	2.3047824	0	0	0	8072	tags=82%, list=41%, signal=139%
GCM_CSNK2B	98	0.56095266	2.303643	0	0	0	7609	tags=81%, list=39%, signal=132%
MODULE_357	76	0.5848927	2.2981043	0	0	0	1789	tags=37%, list=9%, signal=40%
GNF2_FBL	145	0.52505535	2.2934113	0	0	0	5833	tags=61%, list=30%, signal=87%
MODULE_29	27	0.7299376	2.2928236	0	0	0	4785	tags=93%, list=25%, signal=123%
MORF_UBE2I	238	0.49950063	2.2902822	0	0	0	7819	tags=71%, list=40%, signal=117%
MODULE_154	71	0.5919356	2.2594893	0	8.69E-05	0.001	1789	tags=37%, list=9%, signal=40%
GNF2_DAP3	118	0.5474657	2.259309	0	8.32E-05	0.001	6928	tags=72%, list=36%, signal=111%
MODULE_297	76	0.58601916	2.2580717	0	7.97E-05	0.001	1789	tags=37%, list=9%, signal=40%
MORF_ANP32B	197	0.49914193	2.2527962	0	7.65E-05	0.001	7939	tags=75%, list=41%, signal=125%
GCM_APEX1	113	0.5416784	2.2478492	0	7.36E-05	0.001	6935	tags=73%, list=36%, signal=113%
GNF2_GLTSCR2	31	0.6947166	2.234576	0	1.40E-04	0.002	4785	tags=94%, list=25%, signal=124%
GNF2_SPRR1B	21	0.75724036	2.2250254	0	1.35E-04	0.002	1541	tags=57%, list=8%, signal=62%
GCM_ACTG1	123	0.5223154	2.2139533	0	1.87E-04	0.003	7113	tags=72%, list=37%, signal=112%
MORF_ERH	114	0.52058136	2.2041113	0	1.81E-04	0.003	7947	tags=81%, list=41%, signal=135%
MORF_EIF3S6	122	0.5279552	2.200043	0	1.75E-04	0.003	7947	tags=80%, list=41%, signal=135%
MODULE_298	19	0.7568386	2.1905832	0	1.69E-04	0.003	596	tags=42%, list=3%, signal=43%
MORF_JUND	64	0.5846139	2.18988	0	1.64E-04	0.003	5647	tags=72%, list=29%, signal=101%
MODULE_68	19	0.75790423	2.1639967	0	1.60E-04	0.003	596	tags=42%, list=3%, signal=43%
MORF_G22P1	171	0.48629752	2.160848	0	1.55E-04	0.003	7947	tags=72%, list=41%, signal=120%
MODULE_73	19	0.7453506	2.1528246	0	1.51E-04	0.003	560	tags=53%, list=3%, signal=54%
GNF2_CKS2	50	0.59455144	2.1213331	0	1.47E-04	0.003	4890	tags=58%, list=25%, signal=77%
MORF_EIF4A2	138	0.49196252	2.100293	0	2.38E-04	0.005	6830	tags=64%, list=35%, signal=99%
MODULE_114	332	0.4412614	2.099389	0	2.32E-04	0.005	6614	tags=64%, list=34%, signal=96%
GNF2_CDC20	56	0.569282	2.09359	0	2.26E-04	0.005	5387	tags=63%, list=28%, signal=86%
MODULE_151	311	0.43870503	2.0863914	0	3.06E-04	0.007	6614	tags=65%, list=34%, signal=96%
MODULE_32	237	0.45042282	2.0774214	0	5.56E-04	0.013	7049	tags=65%, list=36%, signal=100%
MODULE_153	32	0.6424479	2.0701852	0	5.84E-04	0.014	727	tags=34%, list=4%, signal=36%
MORF_EIF3S2	243	0.44350612	2.0691514	0	5.71E-04	0.014	7819	tags=64%, list=40%, signal=105%
GNF2_CCNA2	68	0.5379757	2.0674078	0	5.58E-04	0.014	5387	tags=57%, list=28%, signal=79%
GNF2_RAN	85	0.51783055	2.0476763	0	6.27E-04	0.016	7423	tags=71%, list=38%, signal=114%
MODULE_552	16	0.7388666	2.041278	0	6.91E-04	0.017	2623	tags=69%, list=13%, signal=79%
GCM_PFN1	51	0.5614346	2.027505	0	7.47E-04	0.019	6657	tags=69%, list=34%, signal=104%
MODULE_537	17	0.72189254	2.0227401	0.002304148	7.32E-04	0.019	1687	tags=41%, list=9%, signal=45%
MORF_CSNK2B	285	0.4247475	1.9933726	0	0.001108753	0.03	8124	tags=65%, list=42%, signal=110%
GNF2_PTX3	36	0.60176075	1.990988	0.002604167	0.001154729	0.032	3290	tags=50%, list=17%, signal=60%
GNF2_HMMR	47	0.5508787	1.9842057	0	0.001268832	0.036	5387	tags=62%, list=28%, signal=85%
GCM_PPP1CC	56	0.5361963	1.9795676	0	0.001517148	0.044	7609	tags=70%, list=39%, signal=114%
GNF2_CCNB2	57	0.5345916	1.9762547	0	0.001523605	0.045	5387	tags=58%, list=28%, signal=80%

Table S4 (Related to Figure4). Functional annotation for control vs *$BRCA1^{mut}$* -FTE organoids at 1 year by IPA software

	Categories	Diseases or Functions Annotation	p-value	z-score	Gene List	# Molecules
1	Cancer,Organismal Injury and Abnormalities	Non-hematological solid tumor	4.47E-44	1.922	AAAS,AATK,ABCA8,ABC	1023
2	Cancer,Organismal Injury and Abnormalities	Nonhematologic malignant neoplasm	9.97E-44	1.665	AAAS,AATK,ABCA8,ABC	1020
3	Cancer,Organismal Injury and Abnormalities	Tumorigenesis of tissue	1.2E-43	2.413	AAAS,AATK,ABCA8,ABC	1014
4	Cancer,Organismal Injury and Abnormalities	Non-melanoma solid tumor	1.1E-42	2.787	AAAS,AATK,ABCA8,ABC	1015
5	Cancer,Organismal Injury and Abnormalities	Cancer of secretory structure	1.25E-42	0.223	AAAS,AATK,ABCA8,ABC	898
6	Cancer,Organismal Injury and Abnormalities	Epithelial neoplasm	3.1E-42	2.692	AAAS,AATK,ABCA8,ABC	1009
7	Cancer,Endocrine System Disorders,Organismal Injury and Abnormalities	Thyroid cancer	3.32E-42		AAAS,AATK,ABCA8,ABC	833
8	Cancer,Endocrine System Disorders,Organismal Injury and Abnormalities	Thyroid carcinoma	3.8E-42		AAAS,AATK,ABCA8,ABC	832
9	Cancer,Organismal Injury and Abnormalities	Carcinoma	8.37E-42	1.914	AAAS,AATK,ABCA8,ABC	1007
10	Cancer,Endocrine System Disorders,Organismal Injury and Abnormalities	Endocrine carcinoma	9.28E-42		AAAS,AATK,ABCA8,ABC	833
11	Cancer,Endocrine System Disorders,Organismal Injury and Abnormalities	Endocrine cancer	9.87E-42		AAAS,AATK,ABCA8,ABC	834
12	Cancer,Endocrine System Disorders,Organismal Injury and Abnormalities	Nonpituitary endocrine tumor	9.23E-41		AAAS,AATK,ABCA8,ABC	836
13	Cancer,Organismal Injury and Abnormalities	Neck neoplasm	1.59E-40		AAAS,AATK,ABCA8,ABC	836
14	Cancer,Endocrine System Disorders,Organismal Injury and Abnormalities	Endocrine gland tumor	2.44E-40	1.897	AAAS,AATK,ABCA8,ABC	838
15	Cancer,Organismal Injury and Abnormalities	Head and neck carcinoma	4.49E-40		AAAS,AATK,ABCA8,ABC	858
16	Cancer,Organismal Injury and Abnormalities	Head and neck cancer	1.73E-39	2.784	AAAS,AATK,ABCA8,ABC	860
17	Cancer,Organismal Injury and Abnormalities	Extracranial solid tumor	6.39E-39	2.026	AAAS,AATK,ABCA8,ABC	1026
18	Cancer,Organismal Injury and Abnormalities	Malignant solid tumor	1.17E-38	1.182	AAAS,AATK,ABCA8,ABC	1026
19	Cancer,Organismal Injury and Abnormalities	Cancer	4.64E-38	0.85	AAAS,AATK,ABCA8,ABC	1029
20	Cancer,Organismal Injury and Abnormalities	Solid tumor	6.83E-38	1.868	AAAS,AATK,ABCA8,ABC	1029
21	Cancer,Organismal Injury and Abnormalities	Head and neck tumor	1.35E-37	3.063	AAAS,AATK,ABCA8,ABC	862
22	Cancer,Organismal Injury and Abnormalities	Abdominal carcinoma	3.07E-36	2.209	AAAS,AATK,ABCA8,ABC	933
23	Cancer,Organismal Injury and Abnormalities	Abdominal adenocarcinoma	2.32E-33		AAAS,AATK,ABCA8,ABC	885
24	Cancer,Organismal Injury and Abnormalities	Adenocarcinoma	2.44E-32	0.622	AAAS,AATK,ABCA8,ABC	897
25	Cancer,Organismal Injury and Abnormalities	Abdominal cancer	4.6E-32	0.253	AAAS,AATK,ABCA8,ABC	946
26	Cancer,Organismal Injury and Abnormalities	Abdominal neoplasm	7.24E-32	1.959	AAAS,AATK,ABCA8,ABC	955
27	Cancer,Gastrointestinal Disease,Organismal Injury and Abnormalities	Large intestine adenocarcinoma	4.37E-29		AAAS,AATK,ABCA8,ABC	778
28	Cancer,Gastrointestinal Disease,Organismal Injury and Abnormalities	Digestive system cancer	7.54E-28	1.006	AAAS,AATK,ABCA8,ABC	894
29	Cancer,Gastrointestinal Disease,Organismal Injury and Abnormalities	Digestive organ tumor	2.68E-27	1.984	AAAS,AATK,ABCA8,ABC	906
30	Cancer,Gastrointestinal Disease,Organismal Injury and Abnormalities	Gastrointestinal adenocarcinoma	5.95E-27		AAAS,AATK,ABCA8,ABC	786
31	Cancer,Gastrointestinal Disease,Organismal Injury and Abnormalities	Gastrointestinal carcinoma	3.34E-25	1.119	AAAS,AATK,ABCA8,ABC	801
32	Cancer,Gastrointestinal Disease,Organismal Injury and Abnormalities	Large intestine neoplasm	4.27E-23	0.493	AAAS,AATK,ABCA8,ABC	795
33	Cancer,Gastrointestinal Disease,Organismal Injury and Abnormalities	Malignant neoplasm of large intestine	1.2E-22	-0.478	AAAS,AATK,ABCA8,ABC	791
34	Cancer,Gastrointestinal Disease,Organismal Injury and Abnormalities	Gastrointestinal tumor	3.32E-21	0.806	AAAS,AATK,ABCA8,ABC	817
35	Cancer,Gastrointestinal Disease,Organismal Injury and Abnormalities	Gastrointestinal tract cancer	7E-21	-0.169	AAAS,AATK,ABCA8,ABC	813
36	Cancer,Organismal Injury and Abnormalities	Frequency of tumor	7.44E-21	3.807	AAAS,AATK,ABCA8,ABC	694
37	Cancer,Organismal Injury and Abnormalities	Tumorigenesis of epithelial neoplasm	1E-20	3.082	AAAS,AATK,ABCA8,ABC	685
38	Cancer,Organismal Injury and Abnormalities	Development of carcinoma	5.07E-20	2.706	AAAS,AATK,ABCA8,ABC	675
39	Cancer,Organismal Injury and Abnormalities	Development of malignant tumor	8.04E-20	3.033	AAAS,AATK,ABCA8,ABC	681
40	Cancer,Organismal Injury and Abnormalities	Incidence of tumor	3.92E-19	4.033	AAAS,AATK,ABCA8,ABC	704
41	Cancer,Organismal Injury and Abnormalities	Genitourinary tumor	1.12E-15	1.258	AAAS,AATK,ABCA8,ABC	675
42	Cancer,Organismal Injury and Abnormalities	Endometrioid carcinoma	3.3E-15		AAAS,ABCA8,ABCB1,AB	318
43	Cancer,Organismal Injury and Abnormalities	Pelvic adenocarcinoma	3.61E-15		AAAS,ABCA8,ABCB1,AB	499
44	Cancer,Gastrointestinal Disease,Organismal Injury and Abnormalities	Colorectal adenocarcinoma	3.73E-15		AAAS,AATK,ABCA8,ABC	425
45	Cancer,Organismal Injury and Abnormalities	Genitourinary adenocarcinoma	5.15E-15		AAAS,ABCA8,ABCB1,AB	540
46	Cancer,Organismal Injury and Abnormalities	Malignant genitourinary solid tumor	6.32E-15	-0.107	AAAS,AATK,ABCA8,ABC	661
47	Cancer,Organismal Injury and Abnormalities,Reproductive System Disease	Genital tumor	1.1E-14	1.501	AAAS,AATK,ABCA8,ABC	573
48	Cancer,Organismal Injury and Abnormalities	Pelvic tumor	1.33E-14	1.616	AAAS,AATK,ABCA8,ABC	604
49	Cancer,Organismal Injury and Abnormalities	Genitourinary carcinoma	2.01E-14	0.896	AAAS,ABCA8,ABCB1,AB	590
50	Cancer,Gastrointestinal Disease,Organismal Injury and Abnormalities	Colon adenocarcinoma	3.18E-14		AAAS,AATK,ABCA8,ABC	408
51	Cancer,Organismal Injury and Abnormalities	Pelvic cancer	3.19E-14		AAAS,AATK,ABCA8,ABC	594
52	Cancer,Gastrointestinal Disease,Organismal Injury and Abnormalities	Colorectal carcinoma	5.28E-14		AAAS,AATK,ABCA8,ABC	429
53	Cancer,Organismal Injury and Abnormalities,Reproductive System Disease	Genital tract cancer	5.9E-14		AAAS,AATK,ABCA8,ABC	561
54	Cancer,Gastrointestinal Disease,Organismal Injury and Abnormalities	Colon carcinoma	8.61E-14		AAAS,AATK,ABCA8,ABC	411
55	Cancer,Organismal Injury and Abnormalities	Development of adenocarcinoma	1.58E-13	0.948	AAAS,ABCA8,ABCB1,AB	453
56	Cancer,Organismal Injury and Abnormalities,Reproductive System Disease	Uterine endometrioid carcinoma	3.58E-13		AAAS,ABCA8,ABCB1,AB	260
57	Cancer,Gastrointestinal Disease,Organismal Injury and Abnormalities	Colon tumor	3.97E-13	0.366	AAAS,AATK,ABCA8,ABC	428
58	Cancer,Organismal Injury and Abnormalities	Anogenital cancer	4.76E-13	0.199	AAAS,AATK,ABCA8,ABC	612
59	Cancer,Organismal Injury and Abnormalities,Reproductive System Disease	Female genital tract adenocarcinoma	7.7E-13		AAAS,ABCA8,ABCB1,AB	352
60	Cancer,Organismal Injury and Abnormalities,Reproductive System Disease	Endometrioid endometrial adenocarcinoma	9.85E-13		AAAS,ABCA8,ABCB1,AB	255
61	Cancer,Gastrointestinal Disease,Organismal Injury and Abnormalities	Colon cancer	1.02E-12	-1.067	AAAS,AATK,ABCA8,ABC	422
62	Cancer,Organismal Injury and Abnormalities,Reproductive System Disease	Endometrial adenocarcinoma	1.28E-12		AAAS,ABCA8,ABCB1,AB	264
63	Cancer,Gastrointestinal Disease,Organismal Injury and Abnormalities	Development of digestive organ tumor	1.77E-12	3.047	AAAS,AATK,ABCA8,ABC	470
64	Cancer,Organismal Injury and Abnormalities,Reproductive System Disease	Endometrial carcinoma	2.86E-12		AAAS,ABCA8,ABCB1,AB	291
65	Organismal Injury and Abnormalities,Reproductive System Disease	Abnormality of endometrium	7.38E-12		AAAS,ABCA8,ABCB1,AB	307
66	Cancer,Gastrointestinal Disease,Organismal Injury and Abnormalities	Hepatobiliary carcinoma	8.71E-12	2.138	AAAS,AATK,ABCA8,ABC	426
67	Cancer,Organismal Injury and Abnormalities,Reproductive System Disease	Female genital neoplasm	9.74E-12	1.96	AAAS,ABCA8,ABCB1,AB	414
68	Organismal Injury and Abnormalities,Reproductive System Disease	Abnormal growth in endometrium	9.75E-12		AAAS,ABCA8,ABCB1,AB	306
69	Cancer,Organismal Injury and Abnormalities,Reproductive System Disease	Tumorigenesis of reproductive tract	1.13E-11	1.96	AAAS,ABCA8,ABCB1,AB	413
70	Cancer,Organismal Injury and Abnormalities,Reproductive System Disease	Development of genital tumor	1.52E-11	1.446	AAAS,ABCA8,ABCB1,AB	400
71	Cancer,Organismal Injury and Abnormalities,Reproductive System Disease	Female genital tract cancer	4.62E-11		AAAS,ABCA8,ABCB1,AB	399
72	Cancer,Organismal Injury and Abnormalities	Cancer of head	4.67E-11	2.613	ABCB1,ACVR1,AMER1,A	155
73	Cancer,Organismal Injury and Abnormalities,Reproductive System Disease	Endometrium tumor	5.13E-11		AAAS,ABCA8,ABCB1,AB	301
74	r,Gastrointestinal Disease,Hepatic System Disease,Organismal Injury and Abnorm	Liver carcinoma	7.35E-11	2.138	AAAS,AATK,ABCA8,ABC	401
75	Cancer,Organismal Injury and Abnormalities,Reproductive System Disease	Endometrial cancer	1.05E-10		AAAS,ABCA8,ABCB1,AB	299
76	Cancer,Organismal Injury and Abnormalities,Reproductive System Disease	Breast or gynecological cancer	1.31E-10	0.555	AAAS,ABCA8,ABCB1,AB	484
77	Gastrointestinal Disease,Organismal Injury and Abnormalities	Colorectal disorder	1.53E-10	2.047	AAAS,AATK,ABCA8,ABC	481
78	Cancer,Gastrointestinal Disease,Organismal Injury and Abnormalities	Colorectal tumor	1.67E-10	0.343	AAAS,AATK,ABCA8,ABC	469
79	r,Gastrointestinal Disease,Hepatic System Disease,Organismal Injury and Abnorm	Hepatobiliary system cancer	1.92E-10	2.261	AAAS,AATK,ABCA8,ABC	431
80	Cancer,Organismal Injury and Abnormalities,Reproductive System Disease	Uterine carcinoma	2.41E-10		AAAS,ABCA8,ABCB1,AB	297
81	Cancer,Organismal Injury and Abnormalities	Breast or colorectal cancer	7.27E-10	-0.588	AAAS,AATK,ABCA8,ABC	530
82	Cellular Assembly and Organization,Cellular Function and Maintenance	Organization of cytoplasm	1.05E-09	-4.002	AATK,ABCD3,ABI1,ACTR	184
83	Cancer,Gastrointestinal Disease,Organismal Injury and Abnormalities	Colorectal cancer	1.27E-09	-0.896	AAAS,AATK,ABCA8,ABC	457
84	Lipid Metabolism,Small Molecule Biochemistry	Metabolism of glycosphingolipid	1.48E-09	-2.572	ABCA8,ABCB1,ARSA,AS	21
85	Lipid Metabolism,Small Molecule Biochemistry	Metabolism of membrane lipid derivative	1.55E-09	-3.397	ABCA8,ABCB1,ABHD6,A	69
86	r,Gastrointestinal Disease,Hepatic System Disease,Organismal Injury and Abnorm	Liver cancer	1.65E-09	2.261	AAAS,AATK,ABCA8,ABC	406
87	r,Gastrointestinal Disease,Hepatic System Disease,Organismal Injury and Abnorm	Liver tumor	2.55E-09	2.797	AAAS,AATK,ABCA8,ABC	461
88	Hereditary Disorder,Neurological Disease,Organismal Injury and Abnormalities	Autosomal recessive neurological disorder	3.44E-09		AAAS,ALDH18A1,ALG14	87
89	Lipid Metabolism,Small Molecule Biochemistry	Metabolism of glycolipid	4.08E-09	-2.581	ABCA8,ABCB1,ALG14,AL	25
90	Cancer,Organismal Injury and Abnormalities,Reproductive System Disease	Uterine tumor	4.81E-09		AAAS,ABCA8,ABCB1,AB	320
91	Developmental Disorder	Autism spectrum disorder or intellectual disability	5.76E-09		ABCB1,ALDH18A1,ARH	78
92	Cancer,Organismal Injury and Abnormalities,Reproductive System Disease	Uterine cancer	6.09E-09		AAAS,ABCA8,ABCB1,AB	308
93	Cancer,Gastrointestinal Disease,Organismal Injury and Abnormalities	Oral tumor	6.27E-09		ABCB1,AMER1,ANKRD1	133
94	Cancer,Gastrointestinal Disease,Organismal Injury and Abnormalities	Oral cancer	7.66E-09		ABCB1,AMER1,ANKRD1	132
95	Cancer,Gastrointestinal Disease,Organismal Injury and Abnormalities	Oral squamous cell carcinoma	8.69E-09		ABCB1,AMER1,ANKRD1	126
96	Cell Morphology,Cellular Function and Maintenance	Autophagy of cells	9.75E-09	-2.784	ARSA,ATG3,BAX,BCL2L	62
97	Cell Morphology,Cellular Function and Maintenance	Autophagy	1.38E-08	-2.544	ACBD5,ARSA,ATG3,BAX	68
98	Cellular Assembly and Organization,Cellular Function and Maintenance	Organization of cytoskeleton	1.75E-08	-3.991	AATK,ABI1,ACTR2,ADAP	165
99	astrointestinal Disease,Hepatic System Disease,Organismal Injury and Abnormaliti	Liver lesion	2.34E-08	3.448	AAAS,AATK,ABCA8,ABC	470
100	Cancer,Gastrointestinal Disease,Organismal Injury and Abnormalities	Oral cavity carcinoma	2.42E-08		ABCB1,AMER1,ANKRD1	128
101	Developmental Disorder,Neurological Disease	Mental retardation	5.02E-08		ALDH18A1,ARHGEF6,AF	62
102	Cancer,Gastrointestinal Disease,Organismal Injury and Abnormalities	Upper gastrointestinal carcinoma	5.03E-08		AATK,ABCA8,ABCB1,AB	269
103	Lipid Metabolism,Small Molecule Biochemistry	Metabolism of sphingolipid	6.02E-08	-2.647	ABCA8,ABCB1,ARSA,AS	29
104	Cancer,Gastrointestinal Disease,Organismal Injury and Abnormalities	Upper gastrointestinal tract tumor	6.86E-08	1.124	AATK,ABCA8,ABCB1,AB	281
105	Developmental Disorder	Autism or intellectual disability	0.000000077		ABCB1,ALDH18A1,ARH	65
106	Cancer,Gastrointestinal Disease,Organismal Injury and Abnormalities	Upper gastrointestinal tract cancer	8.57E-08		AATK,ABCA8,ABCB1,AB	279
107	Cellular Assembly and Organization,Cellular Function and Maintenance	Microtubule dynamics	9.77E-08	-3.883	AATK,ABI1,ACTR2,ADAP	142
108	Gene Expression	Transcription of RNA	0.000000233	-3.334	ACTR2,ACVR1,AKIRIN2,	196
109	Cancer,Organismal Injury and Abnormalities,Reproductive System Disease	Malignant neoplasm of male genital organ	0.000000237		AATK,ABCB1,ABCB9,AB	334
110	Cancer,Organismal Injury and Abnormalities,Reproductive System Disease	Male genital neoplasm	0.000000268		AATK,ABCB1,ABCB9,AB	336
111	Cancer,Organismal Injury and Abnormalities,Reproductive System Disease	Prostatic carcinoma	0.000000273		ABCB1,ABCB9,ABCF3,AI	292
112	Cancer,Organismal Injury and Abnormalities,Reproductive System Disease	Prostate cancer	0.000000275		AATK,ABCB1,ABCB9,AB	330
113	Cellular Assembly and Organization	Transport of vesicles	0.000000339	-1.276	AATK,ABI1,APC,BICD2,C	24
114	Cancer,Organismal Injury and Abnormalities,Reproductive System Disease	Prostatic tumor	0.000000342		AATK,ABCB1,ABCB9,AB	331
115	Cancer,Organismal Injury and Abnormalities	Neoplasia of cells	0.000000557	0.711	ABCB1,ABCF1,ABHD15,	463
116	Post-Translational Modification	Ubiquitination of protein	0.0000009	-2.692	AMER1,ATG3,BAG5,BRA	58
117	Organismal Survival	Organismal death	0.000000918	14.072	ABCB1,ABI1,ACVR1,AKI	246
118	Organismal Survival	Morbidity or mortality	0.00000124	14.065	ABCB1,ABI1,ACVR1,AKI	248
119	Cancer,Organismal Injury and Abnormalities,Reproductive System Disease	Prostatic adenocarcinoma	0.00000125		ABCB1,ABCB9,ABCF3,AI	277
120	Lipid Metabolism,Small Molecule Biochemistry	Hydrolysis of glycosylceramide	0.00000145	-2.2	ARSA,GBA2,HEXA,HEXB	5
121	,Hepatic System Development and Function,Hepatic System Disease,Organ Morp	Abnormal morphology of liver	0.00000162		ABCD3,AOAH,APC,ARID	35
122	Cancer,Organismal Injury and Abnormalities	Breast or pancreatic cancer	0.00000171	-0.2	AATK,ABCA8,ABCB1,AB	412
123	ment and Function,Hepatic System Development and Function,Organ Morphology	Morphology of liver	0.00000225		ABCD3,AOAH,APC,ARID	46
124	ental Disorder,Hereditary Disorder,Neurological Disease,Organismal Injury and Ab	Familial mental retardation	0.00000253		ALDH18A1,ARHGEF6,AF	46
125	Gene Expression	Expression of RNA	0.00000326	-2.657	ABCF1,ACO1,ACTR2,AC	231
126	Cell Death and Survival	Cell death of tumor cell lines	0.00000402	3.227	AAAS,AATK,ABCB1,AKIP	170
127	Lipid Metabolism,Molecular Transport,Small Molecule Biochemistry	Accumulation of glycosphingolipid	0.00000479	1.468	ARSA,GALC,GBA2,GULF	8
128	Cancer,Organismal Injury and Abnormalities	Upper aero-digestive squamous cell carcinoma	0.00000483		ABCB1,AMER1,ANKRD1	163
129	Lipid Metabolism,Molecular Transport,Small Molecule Biochemistry	Accumulation of galactosylceramide	0.00000527		ARSA,GALC,PSAP,UGT8	4
130	Neurological Disease	Cognitive impairment	0.00000529	0.965	ALDH18A1,ARHGEF6,AF	80
131	Gene Expression	Transcription of DNA	0.00000569	-3.398	ACTR2,ACVR1,AKIRIN2,	161
132	Infectious Diseases	Infection by HIV-1	0.0000058	-7.644	ALG14,ATMIN,ATXN2,BI	70
133	l Morphology,Cellular Assembly and Organization,Cellular Function and Maintena	Formation of cellular protrusions	0.00000662	-3.12	ABI1,ACTR2,ADAP1,APC	107
134	Cell Death and Survival	Apoptosis of colorectal cancer cell lines	0.00000662	1.517	APC,ATMIN,BAX,BCL2L1	37
135	Hereditary Disorder,Neurological Disease,Organismal Injury and Abnormalities	Familial encephalopathy	0.00000738	-0.358	ALDH18A1,AMER1,ANO	128
136	Cancer,Cardiovascular Disease,Organismal Injury and Abnormalities	Capillary hemangioma	0.00000744		ABCB1,AMER1,BCL2L1,	13
137	Lipid Metabolism,Molecular Transport,Small Molecule Biochemistry	Accumulation of sphingolipid	0.00000837	1.54	ARSA,ASAH1,BCL2L1,C	11
138	Hereditary Disorder,Neurological Disease,Organismal Injury and Abnormalities	Hereditary neuropathy	0.00000857		ALDH18A1,ASAH1,ATL1	37
139	Cell Death and Survival	Apoptosis of tumor cell lines	0.00000915	2.707	AAAS,AATK,ABCB1,AKIP	138
140	Lipid Metabolism,Small Molecule Biochemistry	Synthesis of sphingolipid	0.00000921	-2.231	ABCA8,ABCB1,ARSA,AS	21
141	DNA Replication, Recombination, and Repair	Repair of DNA	0.0000107	-3.697	ASCC3,ATF2,ATMIN,BCL	46
142	Cancer,Organismal Injury and Abnormalities	Melanoma	0.0000123	-0.393	AATK,ABCA8,ABCB1,AB	589
143	Infectious Diseases	Infection of cells	0.0000132	-7.855	ACTR2,ADAL,ALG14,ATM	85
144	Cell Death and Survival	Cell viability of tumor cell lines	0.0000135	-6.073	ABCB1,AGPS,AK3,ASAH	106
145	Gene Expression	Transcription	0.0000231	-3.676	ACO1,ACTR2,ACVR1,AK	214
146	Cancer,Organismal Injury and Abnormalities,Reproductive System Disease	Breast or ovarian carcinoma	0.0000232		ABCA8,ABCB9,ABHD10,	253
147	ellular Growth and Proliferation,Nervous System Development and Function,Orga	Morphogenesis of neurons	0.0000255	-1.997	ABI1,ADAP1,APC,ARHG	74
148	Cancer,Organismal Injury and Abnormalities,Reproductive System Disease	Breast or ovarian cancer	0.0000262	0.152	ABCA8,ABCB1,ABCB9,A	303
149	Cancer,Organismal Injury and Abnormalities	Malignant neoplasm of aerodigestive tract	0.0000264		ABCA8,ABCB1,ACO1,AC	202
150	Lipid Metabolism,Molecular Transport,Small Molecule Biochemistry	Accumulation of lipid	0.0000271	0.831	ARSA,ASAH1,ATF6,ATP	41

**151**	Carbohydrate Metabolism,Lipid Metabolism,Small Molecule Biochemistry	Synthesis of phosphatidylinositol	0.0000308	-0.728	CDS1,HSPA5,INPP4A,IN	20
**152**	Cell Morphology,Cellular Assembly and Organization	Morphology of lysosome	0.0000347		BAX,CLN3,CTSA,DNM2,E	10
**153**	Lipid Metabolism,Small Molecule Biochemistry	Synthesis of phospholipid	0.0000362	-1.293	ABCA8,AGPS,ATF6,CDS	32
**154**	Digestive System Development and Function,Gastrointestinal Disease	Abnormal morphology of digestive system	0.0000384		ABCB1,ABCD3,AOAH,AF	66
**155**	Lipid Metabolism,Small Molecule Biochemistry	Metabolism of phospholipid	0.0000405	-2.279	ABCA8,ABHD6,AGPS,AT	37
**156**	Cell Death and Survival	Cell death of neuronal progenitor cells	0.0000415	0.046	DICER1,ERBB3,HTT,PAX	6
**157**	ental Disorder,Hereditary Disorder,Neurological Disease,Organismal Injury and Ab	Autosomal dominant mental retardation	0.000045		ARID2,ATP6V1B2,CDK1	18
**158**	Cell Death and Survival	Cell viability	0.0000483	-6.186	ABCB1,AGPS,AK3,AMIG	147
**159**	docrine System Disorders,Organismal Injury and Abnormalities,Reproductive Syste	Ovarian tumor	0.0000514		ABCA8,ABCB1,ABCB9,A	168
**160**	Cell Death and Survival	Cell death of connective tissue cells	0.0000519	-0.894	ABCB1,ASAH1,ATF2,ATF	68
**161**	Cellular Function and Maintenance,Cellular Growth and Proliferation,Nervous Syst	Neuritogenesis	0.0000524	-1.997	ABI1,ADAP1,APC,ARHG	72
**162**	Cancer,Organismal Injury and Abnormalities	Upper aerodigestive tract carcinoma	0.0000527		ABCA8,ABCB1,ACO1,AC	196
**163**	Carbohydrate Metabolism,Lipid Metabolism,Small Molecule Biochemistry	Synthesis of phosphatidic acid	0.0000542	-1.673	ATF6,CDS1,CTBP2,DGK	28
**164**	ental Disorder,Hereditary Disorder,Neurological Disease,Organismal Injury and Ab	Dominant mental retardation	0.0000555		ARID2,ATP6V1B2,CDK1	19
**165**	Cell Morphology	Morphology of cellular protrusions	0.0000582		AATK,AP1M2,APC,ARSA	39
**166**	docrine System Disorders,Organismal Injury and Abnormalities,Reproductive Syste	Ovarian cancer	0.0000609		ABCA8,ABCB1,ABCB9,A	164
**167**	d Function,Neurological Disease,Organ Morphology,Organismal Development,Or	Abnormal morphology of Purkinje's layer of cerebellum	0.0000645		ARSA,ATXN2,BICD2,CLN	14
**168**	docrine System Disorders,Organismal Injury and Abnormalities,Reproductive Syste	Gonadal tumor	0.0000664	1.387	ABCA8,ABCB1,ABCB9,A	173
**169**	Cellular Assembly and Organization,Cellular Compromise	Formation of cellular inclusion bodies	0.0000824	-1.721	ATF6,BAG5,BCL2L1,CRY	15
**170**	Cell Death and Survival	Apoptosis	0.0000833	2.677	AAAS,AATK,ABCB1,ABR	254
**171**	Carbohydrate Metabolism,Lipid Metabolism,Small Molecule Biochemistry	Metabolism of phosphatidic acid	0.000087	-2.496	ATF6,CDS1,CTBP2,DGK	30
**172**	Cell Death and Survival	Apoptosis of fibroblast cell lines	0.0000876	-1.495	ABCB1,ASAH1,ATF2,ATF	40
**173**	Cell Cycle	Cell cycle progression	0.0000895	-2.989	ABRAXAS2,AMER1,ANK	111
**174**	Lipid Metabolism,Molecular Transport,Small Molecule Biochemistry	Accumulation of cerebroside	0.0000914	1.165	ARSA,GALC,GBA2,PSAP	5
**175**	Cell Morphology,Cellular Function and Maintenance	Autophagy of tumor cell lines	0.0000922	-2.973	ATG3,BAX,BCL2L1,CRY	25
**176**	Cell Morphology	Morphology of microvilli	0.0000944		AP1M2,PLS1,PLS3,RAB	6
**177**	order,Neurological Disease,Organismal Injury and Abnormalities,Skeletal and Mus	Autosomal dominant hereditary spastic paraplegia	0.0000944		ALDH18A1,ATL1,CPT1C	6
**178**	Cell Death and Survival	Cell death of colorectal cancer cell lines	0.000107	1.335	APC,ATMIN,BAX,BCL2L1	39
**179**	Cellular Assembly and Organization	Opening of cellular membrane	0.00011		BAX,BCL2L1,BID	3
**180**	Cellular Growth and Proliferation,Organismal Development	Growth of yeast	0.000116	0.522	ABCB1,ALG14,ATXN2,B	15
**181**	DNA Replication, Recombination, and Repair	Metabolism of DNA	0.000116	0.016	ABCB1,ANKRD17,APC,A	46
**182**	Cancer,Organismal Injury and Abnormalities	Mixed neoplasia	0.00012		ABCB9,ACADS,ACTR2,A	136
**183**	Cellular Function and Maintenance	Clathrin mediated endocytosis	0.00012	-2.791	ATP6AP1,ATP6V1B2,CD	13
**184**	ent,Cellular Growth and Proliferation,Nervous System Development and Function,T	Development of neurons	0.000122	-3.665	ABI1,ADAP1,AMIGO2,AP	89
**185**	Cell Death and Survival	Cellular degradation	0.000133	2.181	ARSA,ATF2,ATP8B1,BAC	34
**186**	Lipid Metabolism,Small Molecule Biochemistry	Synthesis of glycolipid	0.000135	-2.156	ABCA8,ABCB1,ALG14,AL	14
**187**	docrine System Disorders,Organismal Injury and Abnormalities,Reproductive Syste	Development of gonadal tumor	0.000148		ABCA8,ABCB9,ABHD6,A	141
**188**	Lipid Metabolism,Small Molecule Biochemistry	Synthesis of lipid	0.000154	-3.705	ABCA8,ABCB1,ABCD3,A	87
**189**	docrine System Disorders,Organismal Injury and Abnormalities,Reproductive Syste	Ovarian carcinoma	0.000159		ABCA8,ABCB9,ABHD6,A	147
**190**	Infectious Diseases	HIV infection	0.000164	-7.705	ABCB1,ALG14,ATMIN,AT	75
**191**	Cell Death and Survival,Embryonic Development	Apoptosis of ectoderm	0.000164	2	CUL1,HNF4A,HTT,VPS4	4
**192**	Carbohydrate Metabolism	Synthesis of carbohydrate	0.000185	-1.404	AKR1A1,ALG14,ALG3,AT	59
**193**	Cancer,Hematological Disease,Organismal Injury and Abnormalities	Neoplasia of blood cells	0.00019	2.617	ABCB1,ABI1,ABRAXAS2,	278
**194**	opment and Function,Neurological Disease,Organ Morphology,Organismal Devel	Loss of Purkinje cells	0.00019		ATXN2,EI24,HTT,OSTM1	6
**195**	Cell Death and Survival	Cell death of fibroblast cell lines	0.000197	-1.685	ABCB1,ASAH1,ATF2,ATF	49
**196**	Infectious Diseases	Viral Infection	0.0002	-7.507	AAAS,ABCB1,ABI1,ACTR	153
**197**	Cell Death and Survival	Cell survival	0.000201	-6.138	ABCB1,AGPS,AK3,AMIG	150
**198**	Cell Morphology,Cellular Assembly and Organization	Morphology of mitochondria	0.000214		ACOT13,ATG3,BAX,BCL	20
**199**	er,Hematological Disease,Immunological Disease,Organismal Injury and Abnorm	Acute myeloid leukemia	0.000217	0.272	ABCB1,ACBD5,AFTPH,A	152
**200**	l Morphology,Cellular Assembly and Organization,Cellular Function and Maintena	Formation of microvilli	0.000219		ATP8B1,DAG1,HNF4A,PL	7
**201**	Cancer,Organismal Injury and Abnormalities	Extraadrenal retroperitoneal tumor	0.000221	1.444	AATK,ABCB1,ABCD3,AB	294
**202**	Hereditary Disorder,Neurological Disease,Organismal Injury and Abnormalities	Autosomal dominant neuropathy	0.000223		ALDH18A1,ATL1,CHMP2	13
**203**	Cancer,Gastrointestinal Disease,Organismal Injury and Abnormalities	Gastric carcinoma	0.000224		AATK,ABCF1,ACO1,ACV	130
**204**	Digestive System Development and Function	Morphology of digestive system	0.000226		ABCB1,ABCD3,AOAH,AF	77
**205**	Cell Morphology,Cellular Function and Maintenance	Macroautophagy	0.000226	-1.982	ATG3,CHMP2A,CHMP2B	18
**206**	er,Hematological Disease,Immunological Disease,Organismal Injury and Abnorm	Myeloid leukemia	0.000233	-0.073	ABCB1,ACBD5,AFTPH,A	155
**207**	docrine System Disorders,Organismal Injury and Abnormalities,Reproductive Syste	Ovarian adenocarcinoma	0.000238		ABCA8,ABCB9,ABHD6,A	139
**208**	logy,Nervous System Development and Function,Organ Morphology,Organismal D	Morphology of cerebellar cortex cells	0.000254		ARSA,ATXN2,BICD2,EI2	12
**209**	ental Disorder,Hereditary Disorder,Neurological Disease,Organismal Injury and Ab	Recessive mental retardation	0.00027		ALDH18A1,ARHGEF6,AS	25
**210**	ll Death and Survival,Cellular Compromise,Neurological Disease,Tissue Morpholo	Degeneration of neurons	0.000282	2.136	ARSA,ATF2,ATP8B1,BAC	31
**211**	Cancer,Hematological Disease,Organismal Injury and Abnormalities	Hematopoietic neoplasm	0.000288	2.726	ABCB1,ABI1,ABRAXAS2,	279
**212**	Cell Death and Survival,Embryonic Development	Apoptosis of embryonic cell lines	0.000288	-1.618	ATF2,ATG3,BAX,BCL2L1	34
**213**	Cell Death and Survival,Embryonic Development	Apoptosis of embryonic tissue	0.000289	2.449	AMER1,CUL1,HNF4A,HC	9
**214**	Lipid Metabolism,Small Molecule Biochemistry	Catabolism of lipid	0.000294	-3.093	ABHD6,ACBD5,ARSA,AS	19
**215**	Cancer,Cardiovascular Disease,Organismal Injury and Abnormalities	Hemangioblastoma	0.000295		ABCB1,AMER1,DDX6,DI	10
**216**	Organismal Development	Arrest in growth of organism	0.000296		ACVR1,ATF2,CTCF,CUL	22
**217**	Cancer,Gastrointestinal Disease,Organismal Injury and Abnormalities	Rectal adenocarcinoma	0.000302		ACBD5,AMER1,AMIGO2,	56
**218**	vous System Development and Function,Organ Morphology,Organismal Developm	Morphology of cerebral cortex	0.000309		ARHGAP5,ARL6,BACE1,	30
**219**	d Function,Neurological Disease,Organ Morphology,Organismal Development,Or	Abnormal morphology of cerebellar cortex	0.00033		ARSA,ATXN2,BICD2,CLN	15
**220**	Cancer,Organismal Injury and Abnormalities	Cancer of cells	0.000337	1.953	ABCB1,ABCF1,ABHD15,	374
**221**	Cell Cycle	G2/M phase	0.00035	-0.378	ABCB1,ATF2,BCL2L1,BM	34
**222**	Cancer,Hematological Disease,Organismal Injury and Abnormalities	Bone marrow neoplasm	0.000354	0.439	ABCB1,ABI1,ACBD5,AD	168
**223**	Cancer,Gastrointestinal Disease,Organismal Injury and Abnormalities	Cecum adenocarcinoma	0.000369		AMER1,APC,ARHGAP5,	54
**224**	vous System Development and Function,Organ Morphology,Organismal Developm	Morphology of hippocampus	0.000371		ARL6,BACE1,BICD2,BID,	19
**225**	Carbohydrate Metabolism	Metabolism of carbohydrate	0.00039	-1.517	ABCB1,AKR1A1,ALG14,	74
**226**	l Morphology,Cellular Assembly and Organization,Cellular Function and Maintena	Extension of cellular protrusions	0.000401	-2.693	AATK,ARHGAP20,ARHG	27
**227**	Infectious Diseases	Infection of tumor cell lines	0.000401	-6.15	ACTR2,ADAL,ALG14,BIC	53
**228**	Cell Cycle	Cycling of centrosome	0.000422	-1.546	CCP110,CEP57,CEP68,	16
**229**	Cancer,Hematological Disease,Organismal Injury and Abnormalities	Hematologic cancer	0.000425	2.49	ABCB1,ABRAXAS2,ACB	274
**230**	Cell Morphology	Size of microvilli	0.000425		PLS1,RAB8A,USH1C	3
**231**	Cancer,Gastrointestinal Disease,Organismal Injury and Abnormalities	Right colon tumor	0.000425		APC,KRAS,MSH2	3
**232**	Cancer,Organismal Injury and Abnormalities,Reproductive System Disease	Breast carcinoma	0.000428		ABCA8,ABHD10,ACOT9,	153
**233**	Gene Expression	Activation of DNA endogenous promoter	0.000429	-3.654	ACTR2,ACVR1,AKIRIN2,	122
**234**	Behavior	Habituation	0.000463	-1.342	BACE1,CERS6,CKAP5,F	6
**235**	Cancer,Organismal Injury and Abnormalities	Lymphoreticular neoplasm	0.000473	2.559	ABCB1,ABI1,ABRAXAS2,	245
**236**	phology,Cellular Function and Maintenance,DNA Replication, Recombination, an	Double-stranded DNA break repair of tumor cell lines	0.000473	-0.958	BMI1,DEK,PBRM1,RB1,R	9
**237**	Cancer,Organismal Injury and Abnormalities	Clear-cell adenocarcinoma	0.000501		ABCB1,ACADS,ADAL,AP	91
**238**	Cell Morphology,Cellular Assembly and Organization	Size of lysosome	0.000506		BAX,DNM2,EPS8,HEXA,	5
**239**	Cancer,Hematological Disease,Organismal Injury and Abnormalities	Malignant myeloid neoplasm	0.000512	1.034	ABCB1,ACBD5,ADAL,AF	162
**240**	Cell Morphology,Cellular Function and Maintenance	Macroautophagy of cells	0.000512	-1.715	ATG3,CHMP2A,CHMP2B	17
**241**	Disorder,Neurological Disease,Organismal Injury and Abnormalities,Psychologica	Familial amyotrophic lateral sclerosis	0.000534		BCL2L1,CHMP2B,GRN,M	8
**242**	er,Hematological Disease,Immunological Disease,Organismal Injury and Abnorm	Acute leukemia	0.000536	-0.161	ABCB1,ACBD5,AFTPH,A	161
**243**	Function,Neurological Disease,Organ Morphology,Organismal Development,Org	Abnormal morphology of Purkinje cells	0.000537		ARSA,ATXN2,EI24,FMR1	11
**244**	Cancer,Hematological Disease,Organismal Injury and Abnormalities	Bone marrow cancer	0.00057	0.428	ABCB1,ACBD5,ADAL,AF	163
**245**	Embryonic Development,Organismal Development	Arrest in growth of embryo	0.000572		ACVR1,ATF2,CTCF,CUL	21
**246**	Cell Death and Survival	Neuronal cell death	0.00058	1.418	AATK,AMIGO2,APC,ASA	72
**247**	Cancer,Organismal Injury and Abnormalities	Head and neck squamous cell carcinoma	0.000585		ABCB1,ABCF1,AMER1,A	205
**248**	ental Disorder,Hereditary Disorder,Neurological Disease,Organismal Injury and Ab	Autosomal recessive mental retardation	0.0006		ALDH18A1,ASCC3,CAS	20
**249**	Cancer,Gastrointestinal Disease,Organismal Injury and Abnormalities	Gastric adenocarcinoma	0.000628		ABCF1,ACO1,ACVR1,AD	122
**250**	Lipid Metabolism,Small Molecule Biochemistry	Synthesis of glycosphingolipid	0.000643	-1.922	ABCA8,ABCB1,ARSA,B4	9
**251**	Protein Synthesis	Homo-oligomerization of protein	0.000655		AK3,APC,ATL1,ATL2,BA	22
**252**	Lipid Metabolism,Molecular Transport,Small Molecule Biochemistry	Concentration of lipid	0.000667	-0.808	ABCB1,ABHD6,ACOT13,	85
**253**	Protein Synthesis	Metabolism of protein	0.000669	-1.871	ABCF1,ACO1,ACR,AKIP1	117
**254**	Cancer,Gastrointestinal Disease,Organismal Injury and Abnormalities	Rectum cancer	0.000672		ACBD5,AMER1,AMIGO2,	57
**255**	Cancer,Hematological Disease,Organismal Injury and Abnormalities	Myeloid neoplasm	0.000672	0.729	ABCB1,ABI1,ACBD5,AD	167
**256**	Cell Cycle	Interphase	0.000688	-1.837	ABCB1,ACVR1,APC,ATF	79
**257**	Infectious Diseases,Organismal Injury and Abnormalities	Infection of embryonic cell lines	0.000699	-5.559	ATMIN,ATXN2,CD164,CE	31
**258**	Auditory Disease,Neurological Disease	Hearing loss	0.000704	3.07	ABCB1,ARSA,ATP6V1B2	29
**259**	Cellular Function and Maintenance,Cellular Growth and Proliferation,Nervous Syst	Polarization of neurons	0.000706		APC,GSK3B,HTT,KIF3A,F	5
**260**	Cell Death and Survival	Cell death of lung cancer cell lines	0.000717	-0.296	AMER1,BAX,BCL2L1,BID	35
**261**	Cellular Function and Maintenance	Maintenance of stem cells	0.000725		APC,BMPR1A,DDX6,DIC	12
**262**	Protein Degradation,Protein Synthesis	Catabolism of protein	0.000742	-2.074	ACR,ANAPC10,APC,ATF	67
**263**	Cell Death and Survival,DNA Replication, Recombination, and Repair	Fragmentation of DNA	0.000744	-0.642	ABCB1,ASAH1,BAX,BCL	21
**264**	Cancer,Organismal Injury and Abnormalities,Reproductive System Disease	Breast cancer	0.000746	0.152	ABCA8,ABCB1,ABHD10,	203
**265**	Cellular Compromise	Degeneration of cells	0.000757	3.312	ARSA,ATF2,ATP8B1,BAC	40
**266**	logical Disease,Hereditary Disorder,Immunological Disease,Organismal Injury an	Familial acute myeloid leukemia	0.000769		APC,CHIC2,KRAS,MSH2	6
**267**	Infectious Diseases	Infection of epithelial cell lines	0.000775	-5.468	ATMIN,ATXN2,CD164,CE	30
**268**	Cancer,Gastrointestinal Disease,Organismal Injury and Abnormalities	Intestinal gastric adenocarcinoma	0.000775		ABCF1,ADAL,APC,CEP6	30
**269**	Cell Morphology,Nervous System Development and Function,Organ Morphology,C	Loss of brain cells	0.000782		ATXN2,CHMP2B,DICER1	10
**270**	Cell Cycle,Cellular Development,Connective Tissue Development and Function	Senescence of fibroblast cell lines	0.000827	-0.682	ARHGAP5,BMI1,BRD7,D	19
**271**	Cell Death and Survival	Apoptosis of connective tissue cells	0.000836	-0.38	ATF2,ATF6,BAX,BCL2L1,	34
**272**	Molecular Transport,Protein Trafficking	Transport of protein	0.000852	-1.965	ABCB9,AP1M2,APPBP2,	35
**273**	Cancer,Gastrointestinal Disease,Organismal Injury and Abnormalities	Gastroesophageal adenocarcinoma	0.000871		ABCA8,ABCB1,ABCF1,A	146
**274**	Drug Metabolism,Molecular Transport,Small Molecule Biochemistry	Transport of vinblastine	0.000877		ABCB1,HSF1,RALBP1,RI	4
**275**	Embryonic Development,Organismal Development,Tissue Development	Development of epiblast	0.000877		APC,EED,IHH,SBDS	4
**276**	Cellular Assembly and Organization	Expansion of endoplasmic reticulum	0.000877	0.254	ATF6,ATG3,HSPA5,SUC	4
**277**	al Disease,Hepatic System Disease,Inflammatory Disease,Organismal Injury and	Primary sclerosing cholangitis	0.000887		BAX,BCL2L1,CASP2,CAS	8
**278**	Infectious Diseases	Infection of cervical cancer cell lines	0.000933	-5.771	ADAL,ALG14,BICD2,BMF	44
**279**	Cancer,Gastrointestinal Disease,Organismal Injury and Abnormalities	Pancreatobiliary tumor	0.000943	1.664	AATK,ABCD3,ABCD4,AB	258
**280**	hydrate Metabolism,Lipid Metabolism,Molecular Transport,Small Molecule Bioche	Concentration of phosphatidic acid	0.000959	-1.27	BAX,BID,CDS1,CERS6,C	20
**281**	logy,Nervous System Development and Function,Organ Morphology,Organismal D	Morphology of brain cells	0.000959		ARSA,ATXN2,BICD2,BID	20
**282**	Cellular Development,Cellular Growth and Proliferation	Colony formation of bone cancer cell lines	0.000961	0.943	BMI1,CNOT7,DICER1,MT	5
**283**	order,Neurological Disease,Organismal Injury and Abnormalities,Skeletal and Mus	Hereditary spastic paraplegia	0.000966		ALDH18A1,ATL1,BICD2,	11
**284**	Cell Cycle	Mitosis	0.000978	-1.254	ABRAXAS2,ANKS1A,BR	53
**285**	Cell Morphology	Permeabilization of mitochondria	0.000988	-0.908	BAX,BCL2L1,BID,CASP2	9
**286**	em Development and Function,Organ Morphology,Organismal Development,Tissu	Morphology of intestinal epithelium	0.000989		ABCB1,AP1M2,DICER1,	7
**287**	Cell Morphology,Nervous System Development and Function,Tissue Morphology	Morphology of neurons	0.00102		AATK,ARSA,ATXN2,BAC	65
**288**	Cancer,Neurological Disease,Organismal Injury and Abnormalities	Medulloblastoma	0.00102	2.425	APC,BAX,BCL2L1,BMI1,	21
**289**	Cellular Function and Maintenance	Movement of centrosome	0.00102		APC,GSK3B,PARD6A	3
**290**	Cancer,Hereditary Disorder,Organismal Injury and Abnormalities	Turcot syndrome	0.00102		APC,MSH2,PMS2	3
**291**	Hereditary Disorder,Nutritional Disease,Organismal Injury and Abnormalities	Autosomal recessive obesity	0.00102		FTO,INPP5E,TUB	3
**292**	Lipid Metabolism,Small Molecule Biochemistry	Degradation of glycosylceramide	0.00102		ARSA,GBA2,PSAP	3
**293**	Developmental Disorder,Neurological Disease,Nutritional Disease	Obesity and intellectual disability	0.00102		INPP5E,KIDINS220,PAX	3
**294**	Cell Morphology,Cellular Development	Branching of cells	0.00103	-1.546	ABI1,ADAP1,ATF6,BACE	45
**295**	Cancer,Organismal Injury and Abnormalities,Renal and Urological Disease	Renal clear cell adenocarcinoma	0.00103		ABCB1,ACADS,ADAL,AP	86
**296**	Cancer,Neurological Disease,Organismal Injury and Abnormalities	Cerebellar neoplasm	0.00107	2.425	APC,BAX,BCL2L1,BMI1,	22
**297**	Cellular Function and Maintenance	Maintenance of cells	0.00108		APC,BMPR1A,DDX6,DIC	19
**298**	Nervous System Development and Function,Tissue Morphology	Morphology of nervous tissue	0.00109		AATK,ARSA,ATXN2,BAC	66
**299**	Cell Death and Survival	Apoptosis of fibroblasts	0.00116	-1.283	ATF2,BAX,BCL2L1,BID,C	26
**300**	Lipid Metabolism,Small Molecule Biochemistry	Cleavage of sphingolipid	0.00119	-2.584	ARSA,ASAH1,GALC,GBA	7

301	Neurological Disease	Neurodegeneration	0.0012	2.584	ARSA,ATF2,ATP8B1,BAC	37
302	Cellular Development,Cellular Growth and Proliferation	Colony formation of sarcoma cell lines	0.00121	1.226	BMI1,CNOT7,DICER1,MT	6
303	Cellular Compromise,DNA Replication, Recombination, and Repair	Chromosomal instability	0.00121	2.488	ABRAXAS2,APC,CDK12,	12
304	Cancer,Organismal Injury and Abnormalities,Reproductive System Disease	Mammary tumor	0.00121	0.36	ABCA8,ABCB1,ABHD10,	212
305	Neurological Disease	Degeneration of nervous system	0.00123	2.375	ARSA,ATF2,ATP8B1,BAC	34
306	Organismal Development	Morphology of head	0.00127		AATK,AMER1,ARHGAP5	101
307	Cell Death and Survival	Cell viability of gastrointestinal stromal tumor cell lines	0.00128	-2.224	IGF2R,ZNF254,ZNF429,Z	5
308	Cell Death and Survival	Apoptosis of spinal neuron	0.00128	-1.446	BAX,BCL2L1,CASP2,PAF	5
309	Cancer,Gastrointestinal Disease,Organismal Injury and Abnormalities	Stomach tumor	0.00128		AATK,ABCB1,ABCF1,AC	139
310	Cellular Function and Maintenance	Cellular homeostasis	0.00129	-2.896	ABCD3,ACBD5,ACO1,AC	155
311	Cell Death and Survival	Apoptosis of lung cancer cell lines	0.0013	-0.369	AMER1,BAX,BCL2L1,BID	30
312	Cell Death and Survival	Apoptosis of neuronal progenitor cells	0.00133	1.067	DICER1,ERBB3,HTT,RB1	4
313	Cell Death and Survival,Embryonic Development	Anoikis of embryonic cell lines	0.00133		KRAS,RB1,RBL2,TSC1	4
314	Cell Morphology	Permeabilization of liposome	0.00133	-1.067	BAX,BID,FGF1,RB1	4
315	Organismal Development	Growth of organism	0.00133	-1.39	ABCB1,ACVR1,ALG14,A	83
316	DNA Replication, Recombination, and Repair,Gene Expression	Methylation of DNA	0.00134	0.52	APC,ARID4A,ATF7IP,CT	11
317	Cell Morphology,Cellular Function and Maintenance	Repair of tumor cell lines	0.00134	-1.569	BMI1,DEK,PARG,PBRM1	11
318	Infectious Diseases	Infection of kidney cell lines	0.00136	-5.468	ATMIN,ATXN2,CD164,CE	30
319	Cell Death and Survival	Cell death of fibroblasts	0.00136	-0.522	ATF2,BAX,BCL2L1,BID,C	30
320	Cell Death and Survival	Necrosis	0.00136	2.684	AAAS,AATK,ABCB1,AKIP	244
321	Cancer,Organismal Injury and Abnormalities	Liquid tumor	0.00137	0.342	ABCB1,ABI1,ACBD5,AC	198
322	Cycle,Cellular Assembly and Organization,DNA Replication, Recombination, and F	Segregation of chromosomes	0.00137	-1.371	ABRAXAS2,APC,CHMP2	17
323	ive Tissue Disorders,Organismal Injury and Abnormalities,Skeletal and Muscular D	Bone deformity	0.00138	1.982	AMER1,CHPF,CSK,CTSK	21
324	er,Hematological Disease,Immunological Disease,Organismal Injury and Abnorm	Leukemia	0.00138	-0.104	ABCB1,ACBD5,ACOT9,A	188
325	Cellular Assembly and Organization,Cellular Function and Maintenance	Organization of nucleus	0.0014		APC,CHMP2A,CHMP2B,	18
326	Neurological Disease,Organismal Injury and Abnormalities	Gliosis	0.0014	2.305	AGPS,ARSA,BACE1,BID,	18
327	Cell Cycle	Quiescence	0.00141		BID,DEK,DICER1,DYRK1	7
328	vous System Development and Function,Organ Morphology,Organismal Developn	Size of brain	0.00142		AATK,ARHGAP5,ATF2,B	22
329	Cancer,Organismal Injury and Abnormalities	Malignant neoplasm of retroperitoneum	0.00144	-0.447	AATK,ABCB1,ABCD3,AB	262
330	Carbohydrate Metabolism	Quantity of carbohydrate	0.00146	-2.178	ABCB1,AP1M2,ATF6,AT	57
331	Infectious Diseases	Infection by RNA virus	0.00148	-7.878	ABCB1,ACTR2,ADAL,AL	87
332	Neurological Disease,Skeletal and Muscular Disorders	Paralysis	0.00149	2.608	ALDH18A1,ATL1,BICD2,	25
333	Cell Death and Survival	Apoptosis of rhombencephalon	0.0015	-1.067	ATF2,BAX,EP300,GSK3B	6
334	Lipid Metabolism,Small Molecule Biochemistry	Catabolism of sphingolipid	0.0015	-2.2	ARSA,ASAH1,GBA2,HEX	6
335	Cancer,Gastrointestinal Disease,Organismal Injury and Abnormalities	Gastric cancer	0.00151		AATK,ABCB1,ABCF1,AC	137
336	Cell Cycle	G2 phase	0.00152	0.174	ABCB1,ATF2,BAX,BCL2L	37
337	Cellular Assembly and Organization	Organization of organelle	0.00152	-1.664	ABCD3,APC,ATL1,ATL2,	65
338	Developmental Disorder,Neurological Disease	Global developmental delay with intellectual disability	0.00161		CYFIP1,LOC102724788/	10
339	Hepatic System Development and Function,Hepatic System Disease,Organ Morph	Abnormal morphology of hepatocytes	0.00162		CLN3,CTHRC1,ENTPD5,	12
340	Cell Morphology,Cellular Function and Maintenance	Autophagy of cervical cancer cell lines	0.00162	-0.059	BAX,BCL2L1,CRYAB,MA	8
341	vous System Development and Function,Organ Morphology,Organismal Developn	Morphology of brain	0.00164		AATK,ARHGAP5,ARL6,A	57
342	Embryonic Development,Organismal Development	Patterning of body axis	0.00164		APC,BMPR1A,GRSF1,HN	16
343	RNA Damage and Repair	Destabilization of mRNA	0.00167		FTO,GIGYF2,METTL3,PU	5
344	Lipid Metabolism,Small Molecule Biochemistry	Catabolism of glycosphingolipid	0.00167	-1.964	ARSA,GBA2,HEXA,HEXB	5
345	Cancer,Neurological Disease,Organismal Injury and Abnormalities	Brain cancer	0.00168	2.429	ACVR1,APC,BAX,BCL2L	28
346	Hereditary Disorder,Neurological Disease,Organismal Injury and Abnormalities	Familial motor neuron disease	0.00171		ASAH1,BCL2L1,BICD2,C	14
347	Cellular Movement,Connective Tissue Development and Function	Cell movement of fibroblast cell lines	0.00173	-1.557	ABI1,ACTR2,ARHGEF1,A	24
348	Lipid Metabolism,Molecular Transport,Small Molecule Biochemistry	Concentration of phospholipid	0.00173	-0.563	ABHD6,BAX,BID,CDS1,C	24
349	Developmental Disorder	Global developmental delay	0.00182		CYFIP1,DICER1,KAT6A,L	15
350	Carbohydrate Metabolism,Lipid Metabolism,Small Molecule Biochemistry	Dephosphorylation of phosphatidylinositol	0.00183		INPP5D,INPP5E,INPP5F,	6
351	docrine System Disorders,Organismal Injury and Abnormalities,Reproductive Syste	Mixed ovarian carcinoma	0.00183		ABCB9,ACADS,ACTR2,A	84
352	vous System Development and Function,Organ Morphology,Organismal Developn	Size of cerebral ventricles	0.00191		ATF2,DICER1,HSF2,VAM	4
353	Neurological Disease	Incoordination	0.00191		ATXN2,FMR1,HTT,PRNP	4
354	Auditory Disease,Neurological Disease	Sensorineural hearing loss	0.00196	1	ABCB1,ATP6V1B2,BAX,	15
355	Embryonic Development,Organismal Development,Tissue Morphology	Size of embryo	0.00197		ACVR1,BMPR1A,BRAP,	37
356	Cancer,Organismal Injury and Abnormalities,Tissue Morphology,Tumor Morpholog	Volume of benign tumor	0.00198		APC,KRAS,NR3C1	3
357	Cellular Assembly and Organization,Cellular Compromise	Disruption of chromosome components	0.00198		HSF1,NR3C1,RB1	3
358	Carbohydrate Metabolism,Lipid Metabolism,Small Molecule Biochemistry	Cleavage of phosphatidylinositol-3,4,5-triphosphate	0.00198		INPP5D,INPP5E,INPP5F	3
359	Cell Death and Survival,Embryonic Development	Cell death of embryonic tissue	0.002	2.646	AMER1,CUL1,HNF4A,HC	10
360	Embryonic Development,Organismal Development	Patterning of rostrocaudal axis	0.002		APC,BMPR1A,GRSF1,HN	14
361	Cellular Assembly and Organization	Transport of endosomes	0.00201		AP5M1,CHMP2A,CHMP2	11
362	pmental Disorder,Embryonic Development,Organismal Development,Tissue Morp	Abnormal morphology of embryo	0.00209		ABI1,ACVR1,AMER1,ANI	58
363	Cell Death and Survival	Cell death of forebrain	0.00213	0.414	BAX,BCL2L1,CASP2,CAS	9
364	docrine System Disorders,Organismal Injury and Abnormalities,Reproductive Syste	Ovarian endometrioid carcinoma	0.00214		ABCB9,ACADS,ACTR2,A	85
365	Cancer,Organismal Injury and Abnormalities	Initiation of neoplasia	0.00214		APC,BCL2L1,KRAS,SPIN	5
366	Nervous System Development and Function	Morphology of nervous system	0.00218		AATK,APC,ARHGAP5,AR	96
367	Lipid Metabolism,Small Molecule Biochemistry	Cleavage of glycosphingolipid	0.00221	-2.415	ARSA,GALC,GBA2,HEXA	6
368	Cellular Assembly and Organization,Cellular Function and Maintenance	Organization of Golgi apparatus	0.00223		ATL2,BAG5,CLASP1,CO	10
369	anization,Cellular Function and Maintenance,Cellular Movement,Nervous System	Axonal transport	0.00223		AATK,FMR1,GSK3B,HTT,	10
370	Skeletal and Muscular Disorders	Abnormality of limb	0.0023	-0.57	ABCB1,AMER1,ATL1,ATI	36
371	Cell Morphology,Cellular Function and Maintenance	Transmembrane potential of tumor cell lines	0.0023		BCL2L1,HNF4A	2
372	rder,Hereditary Disorder,Metabolic Disease,Neurological Disease,Organismal Inju	Infantile Sandhoff disease	0.0023		HEXB,PSAP	2
373	sembly and Organization,Cellular Compromise,DNA Replication, Recombination,	Disruption of chromatin	0.0023		HSF1,RB1	2
374	Cellular Compromise	Destabilization of lipid bilayer	0.0023		BCL2L1,BID	2
375	Cancer,Cardiovascular Disease,Organismal Injury and Abnormalities	Pyogenic granuloma	0.0023		BCL2L1,GNA11	2
376	logical Disease,Hereditary Disorder,Immunological Disease,Organismal Injury an	Hereditary type M7 acute myeloid leukemia	0.0023		APC,MSH2	2
377	isorder,Gastrointestinal Disease,Organismal Development,Organismal Injury and	Frontal bossing	0.0023		HEXA,HEXB	2
378	al Disorder,Endocrine System Disorders,Organ Morphology,Organismal Injury and	Hypertrophy of adrenal cortex	0.0023		HTT,NR3C1	2
379	,Organismal Development,Organismal Injury and Abnormalities,Skeletal and Mus	Abnormal morphology of broad rib	0.0023		HEXA,HEXB	2
380	Disorder,Hereditary Disorder,Organismal Injury and Abnormalities,Skeletal and Mu	Severe X-linked myotubular myopathy	0.0023		DNM2,MTM1	2
381	eplication, Recombination, and Repair,Embryonic Development,Organ Developm	Formation of cajal bodies	0.0023		COIL,TOE1	2
382	Cancer,Gastrointestinal Disease,Organismal Injury and Abnormalities	Adenoma of ampulla of Vater	0.0023		APC,KRAS	2
383	Neurological Disease,Organismal Injury and Abnormalities,Psychological Disorder	Abnormal putamen	0.0023		HSF1,HTT	2
384	lar Assembly and Organization,Cellular Function and Maintenance,Molecular Trar	Transcytosis of Golgi vesicles	0.0023		OSBPL1A,OSBPL2	2
385	nflammatory Disease,Inflammatory Response,Ophthalmic Disease,Organismal Inju	Hereditary keratitis	0.0023		COL17A1,PAX6	2
386	Cancer,Gastrointestinal Disease,Organismal Injury and Abnormalities	Sigmoid colon cancer	0.0023		APC,MSH2	2
387	Lipid Metabolism,Small Molecule Biochemistry	Hydrolysis of gangliotriaosylceramide II3-sulfate	0.0023		HEXA,HEXB	2
388	Carbohydrate Metabolism	Transduction of carbohydrate	0.0023		INPP5D,TRAF6	2
389	Lipid Metabolism,Small Molecule Biochemistry	Transduction of lipid	0.0023		INPP5D,TRAF6	2
390	Cell Death and Survival	ernucleosomal DNA fragmentation of lymphoma cell lin	0.0023		BAX,BCL2L1	2
391	Organ Morphology,Tissue Morphology	Permeability of skin tissue	0.0023		NR3C1,PRSS8	2
392	Organismal Functions	Healing of enterocytes	0.0023		TFF3,VANGL1	2
393	Cell Cycle	Cleavage of colorectal cancer cell lines	0.0023		DEK,LOC102724788/PR	2
394	Lipid Metabolism,Small Molecule Biochemistry	Hydrolysis of lactosylceramide II3-sulfate	0.0023		HEXA,HEXB	2
395	l Repair,Endocrine System Disorders,Organ Morphology,Organismal Injury and Ab	Abnormal morphology of cajal bodies	0.0023		COIL,ZPR1	2
396	rder,Hereditary Disorder,Metabolic Disease,Neurological Disease,Organismal Inju	Juvenile GM2 gangliosidosis	0.0023		HEXA,HEXB	2
397	Cellular Assembly and Organization,DNA Replication, Recombination, and Repair	Rearrangement of chromatin	0.0023		NR3C1,TP53BP1	2
398	ervous System Development and Function,Organ Development,Organismal Develo	Development of superior temporal gyrus	0.0023		CNTNAP2,GSK3B	2
399	rder,Hereditary Disorder,Metabolic Disease,Neurological Disease,Organismal Inju	Adult GM2 gangliosidosis	0.0023		HEXA,HEXB	2
400	Behavior	Limb clasping of forelimb	0.0023		DICER1,HTT	2
401	r,Hereditary Disorder,Neurological Disease,Organismal Injury and Abnormalities,F	Familial primary progressive aphasia	0.0023		GRN,PRNP	2
402	Cell Morphology	Permeabilization of phospholipid vesicles	0.0023		BAX,BID	2
403	Neurological Disease,Ophthalmic Disease	Demyelination of optic nerve	0.0023		HSPA5,MYRF	2
404	Cellular Assembly and Organization,DNA Replication, Recombination, and Repair	Quantity of meiotic spindles	0.0023		DICER1,SACM1L	2
405	Hereditary Disorder,Neurological Disease,Organismal Injury and Abnormalities	Amish infantile epilepsy syndrome	0.0023		ATXN2,ST3GAL5	2
406	Compromise,Ophthalmic Disease,Organismal Injury and Abnormalities,Tissue Mo	Degeneration of retinal cells	0.00233	2.229	BACE1,CHM,DICER1,ER	12
407	Cancer,Gastrointestinal Disease,Organismal Injury and Abnormalities	Gastro-esophageal carcinoma	0.00234		AATK,ABCA8,ABCB1,AB	157
408	Hereditary Disorder,Neurological Disease,Organismal Injury and Abnormalities	Hereditary motor neuropathy	0.00235		ALDH18A1,ASAH1,ATL1	17
409	lopment,Skeletal and Muscular Disorders,Skeletal and Muscular System Developr	Abnormal morphology of limb	0.0024		AMER1,ATL1,CCP110,CI	26
410	Cancer,Neurological Disease,Organismal Injury and Abnormalities	Primitive neuroectodermal tumor	0.00252	1.964	APC,BAX,BCL2L1,BMI1,	28
411	RNA Post-Transcriptional Modification	Processing of mRNA	0.00258	0.115	BCAS2,CDC5L,CDK13,C	30
412	Cell Morphology,Cellular Function and Maintenance	Length of cells	0.00262		BAX,BMPR1A,FMR1,GRN	15
413	d Function,Neurological Disease,Organ Morphology,Organismal Development,Or	Abnormal morphology of cerebellum	0.00264		ARSA,ATXN2,BICD2,CLN	18
414	Cell Morphology	Morphology of cervical cancer cell lines	0.00265		BCL2L1,CAP1,CRYAB,C	6
415	Cellular Assembly and Organization	Transport of mitochondria	0.00265	0	APC,GSK3B,HTT,RANBP	6
416	gy,Cellular Function and Maintenance,Renal and Urological System Developmen	Autophagy of kidney cell lines	0.00265	0.277	GSK3B,HTT,RASD2,TBC	6
417	vous System Development and Function,Organ Morphology,Organismal Developn	Size of striatum	0.00266		CPEB4,DICER1,HSF2,HT	4
418	DNA Replication, Recombination, and Repair	Instability of DNA	0.00266		MSH2,RB1,RBL2,TP53B	4
419	Hereditary Disorder,Neurological Disease,Organismal Injury and Abnormalities	Hereditary sensory neuropathy	0.00266		ALDH18A1,ATL1,BICD2,	16
420	DNA Replication, Recombination, and Repair	DNA damage response of cells	0.00267		APC,ATF2,BAX,BAZ1B,B	29
421	Hereditary Disorder,Neurological Disease,Organismal Injury and Abnormalities	Hereditary polyneuropathy	0.00267		ALDH18A1,ATL1,BICD2,	17
422	Lipid Metabolism,Small Molecule Biochemistry	Catabolism of phospholipid	0.00267	-1.949	ABHD6,GDE1,INPP5D,L	7
423	sorders,Developmental Disorder,Organismal Injury and Abnormalities,Skeletal an	Congenital microcephaly	0.00269		DCPS,KAT6A,NUP133,T	5
424	Cell Death and Survival	Apoptosis of progenitor cells	0.00269	0.314	DICER1,ERBB3,HTT,PA	5
425	Cell Death and Survival,Neurological Disease,Organismal Injury and Abnormalitie	Apoptosis of cerebellar granule cell	0.00269	-0.686	ATF2,BAX,EP300,GSK3B	5
426	Cycle,Cellular Assembly and Organization,DNA Replication, Recombination, and F	Duplication of centriole	0.00269	-1.231	CCP110,CEP57,CUL1,M	5
427	Cell Morphology,Cellular Assembly and Organization	Morphology of nucleus	0.00272		APC,CHMP2A,COIL,DEK	22
428	Developmental Disorder,Neurological Disease	Intellectual disability and developmental delay	0.00276		CYFIP1,KAT6A,KDM5B,L	12
429	nology,Nervous System Development and Function,Neurological Disease,Tissue M	Abnormal morphology of neurons	0.00277		AATK,ARSA,ATXN2,BAC	52
430	Cellular Response to Therapeutics	Radiosensitivity	0.00278	1.297	ATG3,BAX,EP300,ERBB	8
431	Neurological Disease	Demyelination of nerves	0.00278	2.804	ARSA,BACE1,DAG1,HSF	8
432	Nutritional Disease,Organismal Injury and Abnormalities	Wasting	0.00281	2.535	ABCB1,ATF6,BACE1,ED	15
433	Ophthalmic Disease,Organismal Injury and Abnormalities	Retinal degeneration	0.00282	2.728	ACBD5,ARL6,BACE1,CH	32
434	Cancer,Cardiovascular Disease,Organismal Injury and Abnormalities	Hemangioma	0.00284		ABCB1,AMER1,BCL2L1,	17
435	Cell Cycle,DNA Replication, Recombination, and Repair	Checkpoint control	0.003	-1.937	BCL2L1,BID,ERCC3,ES	13
436	Organismal Development	Size of body	0.003	-7.785	AAAS,ACOT13,AP1M2,A	81
437	Embryonic Development,Organismal Development	Gastrulation of embryo	0.00309		ACVR1,CLASP1,DAG1,D	7
438	Cell Cycle	Quiescence of cells	0.00316		BID,DICER1,DYRK1A,EP	6
439	sorders,Developmental Disorder,Organismal Injury and Abnormalities,Skeletal an	Microcephaly	0.00322	2.778	BMPR1A,DCPS,DICER1,	15
440	Cell Death and Survival	Cell death of carcinoma cell lines	0.00324	1.142	ATF6,BAX,BCL2L1,BID,B	39
441	Organismal Development	Size of animal	0.0033	-4.197	BACE1,CAPRIN1,CCP11	20
442	y and Organization,Cellular Function and Maintenance,Nervous System Developn	Release of synaptic vesicles	0.00333		PPP3R1,SYT1,VAMP7	3
443	Cellular Compromise,Cellular Function and Maintenance	Endoplasmic reticulum stress response of neurons	0.00333		HTT,MMP9,SIL1	3
444	Cancer,Organismal Injury and Abnormalities	Latency of malignant tumor	0.00333		EED,KRAS,PMS2	3
445	Morphology,Organismal Development,Skeletal and Muscular System Developmen	Diameter of tibia	0.00333		CTSK,HOXA11,LRP5	3
446	Organismal Functions	Jumping	0.00333		HTT,MMP9,PRNP	3
447	Molecular Transport,RNA Trafficking	Accumulation of RNA	0.00335	0.447	ATXN2,DDX6,DIS3L,SBD	5
448	Infectious Diseases	Viral entry by Influenza virus	0.00335	-2.236	ATP6AP1,ATP6V1B2,FAM	5
449	Cellular Function and Maintenance,Molecular Transport,Protein Trafficking	Endocytosis of protein	0.00335		CAP1,DNM2,HTT,KRAS,	5
450	Cancer,Organismal Injury and Abnormalities	Mixed cell carcinoma	0.00337		ABCB9,ACADS,ACTR2,A	86
451	Organismal Functions	Hypoactivity of mice	0.00343		AAAS,ACOT13,ARID4A,C	19
452	DNA Replication, Recombination, and Repair	Chromosomal aberration	0.0035	2.824	ABRAXAS2,APC,BACE1,	22
453	Cellular Assembly and Organization,DNA Replication, Recombination, and Repair	Formation of chromatin	0.00351		BAZ1B,CTCF,GPX4,HDA	11
454	opment,Digestive System Development and Function,Hepatic System Developmer	Differentiation of liver	0.00356	-1.951	FGF1,HNF1B,HNF4A,KR	8
455	Cell Death and Survival	Cell death of striatal neurons	0.00356	0.131	BAX,BCL2L1,CASP2,CA	8
456	Protein Synthesis	Oligomerization of protein	0.00356		AK3,APC,ATL1,ATL2,BA	31
457	Cellular Assembly and Organization	Formation of nuclear speckles	0.00358	-2	HECTD3,TFCP2,XIAP,ZN	4
458	Cell Cycle,Connective Tissue Development and Function	G2/M phase transition of fibroblasts	0.00358		ATF2,JUNB,KRAS,MBD4	4
459	er,Hematological Disease,Immunological Disease,Organismal Injury and Abnorm	Follicular B-cell lymphoma in lymph node	0.00358		EP300,ERBB3,TET2,XIA	4
460	Hair and Skin Development and Function	Pallor	0.00363		ARID3A,COL5A2,FST,KA	14
461	Cellular Assembly and Organization,Cellular Function and Maintenance	Secretion of vesicles	0.00373	-1.432	CRYAB,GSK3B,HTT,PLD	6
462	,Gastrointestinal Disease,Organ Morphology,Organismal Development,Organisma	Abnormal morphology of intestinal epithelium	0.00373		ABCB1,AP1M2,MAPK8,P	6
463	d Function,Neurological Disease,Organ Morphology,Organismal Development,Or	Abnormal morphology of rhombencephalon	0.00383		ARSA,ATXN2,BICD2,CLN	19
464	onnective Tissue Disorders,Hereditary Disorder,Organismal Injury and Abnormaliti	Hereditary connective tissue disorder	0.00383	2.748	ACVR1,AGPS,ALDH18A	63
465	Cell Morphology	Morphology of cilia	0.004		APC,EPS8,FTO,INPP5E,	8
466	Endocrine System Development and Function	Glucose tolerance	0.004	0.847	ACOT13,ATF2,BAX,CER	30
467	Nutritional Disease,Organismal Injury and Abnormalities	Cachexia	0.00406	2.362	ABCB1,ATF6,BACE1,ED	13
468	Cell Cycle	M phase	0.00408	-2.043	APC,BCL2L1,CAP1,CCP	26
469	docrine System Disorders,Organismal Injury and Abnormalities,Reproductive Syste	Adenosquamous ovarian carcinoma	0.0041		ABCB9,ACADS,ACTR2,A	80
470	Cellular Assembly and Organization,Cellular Function and Maintenance	Assembly of multivesicular bodies	0.00411		CHMP2A,CHMP2B,STAM	5
471	Cell Morphology	Abnormal morphology of cilia	0.00411		EPS8,KIAA0586,KIF3A,K	5
472	Cell Death and Survival	Cell viability of epithelial cells	0.00412	-0.555	BCL2L1,COL17A1,KEAP	9
473	Cellular Function and Maintenance	Endocytosis by cervical cancer cell lines	0.00412	-2.96	ABI1,ATP6AP1,ATP6V1B	9
474	Embryonic Development,Organismal Development	Growth of embryo	0.00425	-1.456	ABCB1,ACVR1,ARHGAP	49
475	Nervous System Development and Function,Tissue Morphology	Density of neurons	0.00433	-0.589	ADAP1,BACE1,BAX,BMP	17
476	Post-Translational Modification	Acetylation of protein	0.00438		CLOCK,EP300,HTT,KAT	6
477	Cycle,Cellular Assembly and Organization,DNA Replication, Recombination, and F	Replication of centriole	0.00438	-1.231	CCP110,CEP57,CUL1,M	6
478	Lipid Metabolism,Small Molecule Biochemistry	Hydrolysis of sphingolipid	0.00438	-2.403	ARSA,ASAH1,GBA2,HEX	6
479	Organismal Development	Size of head	0.00438		AATK,AMER1,ARHGAP5	25
480	Cell Death and Survival	Apoptosis of tissue	0.00443	2.63	AMER1,BCL2L1,CUL1,H	10
481	Cellular Assembly and Organization,Cellular Function and Maintenance	Formation of vesicles	0.00444	-1.112	ABI1,ATG3,CALY,CHMP2	18
482	Cell Death and Survival	Apoptosis of male germ cells	0.00446	0.342	BAX,GPX4,HOXA11,HSF	14
483	Cellular Assembly and Organization,DNA Replication, Recombination, and Repair	Formation of chromosome components	0.00448	-2	BAZ1B,BMI1,CTCF,GPX4	15
484	Cell Death and Survival,Neurological Disease,Organismal Injury and Abnormalitie	Apoptosis of granule cells	0.00449	-0.314	AATK,ATF2,BAX,BCL2L1	8
485	etabolic Disease,Organismal Injury and Abnormalities,Renal and Urological Disea	Organic aciduria	0.00449		ABCD4,D2HGDH,DLD,FL	8
486	Cancer,Gastrointestinal Disease,Organismal Injury and Abnormalities	Colorectal adenoma	0.0045		ABCB1,APC,BCL2L1,BID	21
487	Cell Death and Survival,Neurological Disease,Organismal Injury and Abnormalitie	Cell death of granule cells	0.00453	0.16	AATK,ATF2,BAX,BCL2L1	11
488	Cellular Growth and Proliferation,Tissue Development	Proliferation of epithelial cells	0.00455	-0.1	APC,ATF2,BAX,BID,BMP	51
489	RNA Post-Transcriptional Modification	Splicing of RNA	0.00468	0.651	BCAS2,CDC5L,CDK12,C	28
490	and Maintenance,Cellular Growth and Proliferation,Reproductive System Develop	Production of germ cells	0.0047		ARSA,HSF2,PUM1,UBE3	4
491	Post-Translational Modification	Sialylation	0.0047	-1.982	BACE1,CMAS,ST3GAL5,	4
492	velopment and Function,Skeletal and Muscular System Development and Functio	Formation of growth plate	0.0047		GRN,HOXA11,MMP9,TN	4
493	Cell Death and Survival	Apoptosis of neurons	0.00483	1.302	AATK,AMIGO2,ASAH1,A	45
494	nental Disorder,Hereditary Disorder,Metabolic Disease,Organismal Injury and Abn	Lysosomal storage disease	0.00485	2.216	ARSA,ASAH1,CLN3,CTS	12
495	Developmental Disorder	Syndromic developmental delay	0.005		DICER1,KAT6A,KDM5B,	5
496	y Disease,Hereditary Disorder,Neurological Disease,Organismal Injury and Abnor	Hereditary hearing loss	0.005		ATP6V1B2,CCDC50,CD1	19
497	ease,Neurological Disease,Organismal Injury and Abnormalities,Skeletal and Mus	Mitochondrial leukoencephalopathy	0.00502		COX15,DLD,ERCC8,GOL	8
498	Cellular Function and Maintenance,Nervous System Development and Function,T	Length of neurons	0.00503		BMPR1A,FMR1,GRN,HT	13
499	Cellular Compromise,DNA Replication, Recombination, and Repair	Damage of chromosomes	0.00504	2.961	BMI1,CCP110,LIG4,MCM	9
500	Hereditary Disorder,Neurological Disease,Organismal Injury and Abnormalities	Autosomal recessive progressive encephalopathy	0.00504		ASAH1,BCL2L1,FBXO7,	9

Table S5 (Related to STAR Method) Primer sequences

**Primer Name**	**Gene Targeted**	**Application**	**Sequence (5’-3’)**
hBRACHYURY-F	*BRACHYURY*	qRT-PCR	5’-GCTGTGACAGGTACCCAACC-3’
hBRACHYURY-R	*BRACHYURY*	qRT-PCR	5’-CATGCAGGTGAGTTGTCAGAA-3’
hBRCA1-Full-F	*BRCA1*	qRT-PCR	5’-TAGCAAGGAGCCAACATAACAGAT-3’
hBRCA1-Full-R	*BRCA1*	qRT-PCR	5’-CTGTGTGAGAGAAAAGAATGGAATAAG-3’
hBRCA1-DeltaEXON1-F	*BRCA1*	qRT-PCR	5’-GATTCTGCAAAAAAGGCTGCT-3’
hBRCA1-DeltaEXON1-R	*BRCA1*	qRT-PCR	5’-CAGATGCTGCTTCACCCTGA-3’
hGAPDH-F	*GAPDH*	qRT-PCR	5’-GTGGACCTGACCTGCCGTCT-3’
hGAPDH-R	*GAPDH*	qRT-PCR	5’-GGAGGAGTGGGTGTCGCTGT-3’
hGATA3-F	*GATA3*	qRT-PCR	5’-CTCATTAAGCCCAAGCGAAG-3’
hGATA3-R	*GATA3*	qRT-PCR	5’-GTCTGACAGTTCGCACAGGA-3’
hMIXL1-F	*MIXL1*	qRT-PCR	5’-GGTACCCCGACATCCACTT-3’
hMIXL1-R	*MIXL1*	qRT-PCR	5’-GCCTGTTCTGGAACCATACCT-3’
hOCT4-F	POU5F1	qRT-PCR	5’-ACCCACACTGCAGCAGATCA-3’
hOCT4-R	POU5F1	qRT-PCR	5’-CCACACTCGGACCACATCC-3’
hOSR1-F	*OSR1*	qRT-PCR	5’-GGACCTCTGCGGAACAAG-3’
hOSR1-R	*OSR1*	qRT-PCR	5’-TGCAGGGAAGGGTGGATA-3’
hOVGP1-F	*OVGP1*	qRT-PCR	5’-AATTCTCTACCCAGAGTTCAACAAA-3’
hOVGP1-R	*OVGP1*	qRT-PCR	5’-CCGATGGACAGTAGTGTTTTCA-3’
hPAX2-F	*PAX2*	qRT-PCR	5’-GAAGTGCCCCCTTGTGTG-3’

hPAX2-R	*PAX2*	qRT-PCR	5’-TCGTTGTAGGCCGTGTACTG-3’
hSALL1-F	*SALL1*	qRT-PCR	5’-ATTGCAGCCTAGCCAAAAAG-3’
hSALL1-R	*SALL1*	qRT-PCR	5’-ACCAGCTGAGCAGAAAGGTC-3’
hSOX17-F	*SOX17*	qRT-PCR	5’-ACGCCGAGTTGAGCAAGA-3’
hSOX17-R	*SOX17*	qRT-PCR	5’-TCTGCCTCCTCCACGAAG-3’
hSOX2-F	*SOX2*	qRT-PCR	5’-GGGGGAATGGACCTTGTATAG-3’
hSOX2-R	*SOX2*	qRT-PCR	5’-GCAAAGCTCCTACCGTACCA-3’
hWT1-F	WT1	qRT-PCR	5’-GAATGCATGACCTGGAATCA-3’
hWT1-R	WT1	qRT-PCR	5’-TCTGCCCTTCTGTCCATTTC-3’
